# ANCF-based dynamic modeling and control of an active tethered space robot in approaching phase

**DOI:** 10.1038/s41598-026-51564-1

**Published:** 2026-05-14

**Authors:** Ali Kasiri, Farhad Fani Saberi

**Affiliations:** 1 Department of Aerospace Engineering, AMIRKABIR University of Technology (Tehran Polytechnic), Tehran, Iran; 2 Aerospace Sciences and Technology Institute, AMIRKABIR University of Technology (Tehran Polytechnic), Tehran, Iran

**Keywords:** Tethered space robot, ANCF model, Pose motion control, Multi-body dynamics, On-orbit servicing, Engineering, Mathematics and computing, Physics

## Abstract

Herein we analyze the dynamic behavior of a tethered space robot (TSR) for on-orbit capturing, considering large deformation of the tether. The robot is deployed from the mother satellite by using a long, flexible tether and moving along the target through the desired trajectory. The TSR is modeled as a flexible multibody system consisting of a point mass, a rigid body, and a flexible viscoelastic string. In this regard, the mother spacecraft is modeled as a point mass orbiting in a circular Keplerian orbit. The active robot, which is responsible for docking with the target, is modeled with a three-degree-of-freedom equation of motion that reflects its rigid body and free-floating characteristics. The flexible towing tether, which can experience large displacements and deformations, is modeled using an absolute nodal coordinate formulation (ANCF). The equations of motion of the whole system are derived by using Lagrange’s equation. The overall equations of motion and parameters are described in the natural coordinate frame (NCF). Since the point where the tether is attached to the robot is displaced from its center of mass, there is highly nonlinear coupling between tether deformation/tension and robot motion. The robot’s position and attitude are controlled actively to sync with the target based on relative pose motion equations, while the tether is dragged behind it. A numerical simulation based on the final approach scenario was conducted, and the results confirm the physical plausibility of the model.

## Introduction

***The history of tethered space systems:*** the idea of creating a space elevator and a space hotel/residence may have ignited the first sparks of using space tethers^1^. Initially, space tether systems were conceived as a method for transporting heavy payloads over very long distances (e.g., from Earth to GEO) with minimal fuel consumption, known as a ‘green road’. In the early stages (nineteenth century), a space tether was assumed to be very long (hundreds of kilometers) and have very little thickness compared to its length, made by interweaving a series of thin and strong fibers (multiwall carbon nanotubes). The mass of this tether—which could be a way to fulfill all human ambitions in the field of space exploration—reached hundreds of kilograms^2^. It is evident that constructing such a structure requires detailed planning, significant resources, materials, funding, and time. But as time went on and the concept of space tethers grew more developed, a wide range of new fascinating applications including the study of plasma physics and electrical generation in the upper atmosphere^3^, orbiting or deorbiting of space vehicles^4,5^, spacecraft formation flying^6,7^, asteroid rendezvous^8^, interplanetary travels^9^, orbit raise and station keeping^10,11^, orbit transfer^12^, re-entry^13^, space debris removal^14–16^, space/orbital rings^17,20^, space-based solar farm/batteries^21^, orbit escalators^22^, lowering lander robots^23^, space-based microgravity laboratories^24^, etc. gradually emerged. Dealing with each of these systems presents unique challenges due to the fact that the number and configuration (parallel or series) of tethers employed in the applications mentioned above can vary, with some utilizing only one tether and others requiring multiple tethers. Following the diversification of applications, research on tethered satellite systems (TSSs) attracted significant attention and expanded rapidly, leading to the discovery of previously unknown aspects. In this regard, various methods were proposed to model and analyze tether dynamics/behavior in the space environment^25–27^. The considerations, cons and pros, constraints, and limitations of using such systems in real-world conditions became the subject of some other studies^28,29^. Even several experimental efforts were undertaken to learn more about the behavior and motion of a flexible (long and thin) space tether in orbit^30^. Beyond the application of TSSs, some researchers have focused on the tether’s mechanical properties, structure, fabrication methodology, and materials^31–34^, while some others have paid attention to reliable opening and closing (length and tension control) mechanisms of the tether in space^35–37^. A significant issue encountered with long flexible tethers pertains to their vibrations and fluctuations, which pose considerable challenges^38^. Thus, some works present solutions for suppressing^39^ and controlling both in-plane and out-of-plane vibrations^40,41^. A review of the literature suggests that space tether systems are attracting growing interest among researchers in Eastern nations (Russia, China, Japan, and South Korea). To wrap it up, research and studies related to TSSs have reached a rather good maturity in last decade. We are now witnessing the construction of engineering models of such systems and testing them in orbit, and it is not impossible that we will see the widespread use of these systems during the coming decade.

Among the myriad applications of tethered space systems, the provision of in-orbit services represents one of the most promising and immediate opportunities. This application not only holds significant potential for enhancing operational success but also offers robust commercial prospects, making it a focal point for future advancements in space technology.

***The need for on-orbit servicing:*** large spacecraft are extremely valuable assets to any government. However, finite fuel limits their lifespans. Furthermore, a crack or leakage, battery degradation, or failure and malfunction of a hardware/component can lead to the loss of an entire system. Even small space debris—ranging from screws and paint flecks to small gravel—traveling at high orbital velocities poses a significant threat to the health of an active spacecraft. In this regard, while it may be justifiable to send astronauts to repair or replace pieces of unique, valuable, and expensive spacecraft, such as the Hubble Space Telescope or the International Space Station (ISS), but not for all spacecraft. Consequently, in recent years, there has been an increasing demand for autonomous servicer spacecraft/robots. As a result, many companies and space agencies are developing on-orbit servicing (OOS) technologies to enhance the lifespan and ensure the operation of orbiting spacecraft. In this way, OOS missions—including active debris removal, inspection, refueling, in-orbit assembly, in-orbit delivery, on-orbit maintenance, repair, relocation, etc.^42–44^—will undoubtedly play an important role in the future of the space economy^45^. As real-world evidence of this trend, programs such as “O.CUBED”, “SSL”, “MEV-1”, “e.Deorbit”, “Restore-L”, and “Phoenix” are only some of the OOS programs that are being pursued by major international space agencies and companies, including NASA, ESA, Airbus, Northrop Grumman, JAXA, and CSA. The story doesn’t end here, as humanity advances toward ambitious endeavors such as orbital hotels, space elevators, sustained lunar exploration, the establishment of lunar surface bases and habitats, and crewed missions to Mars, the role of in-orbit servicing will assume far greater significance than we currently anticipate.

The central question, however, remains: What essential capabilities and technical specifications must a true multi-role orbital servicing system possess to effectively support this diverse and demanding array of future operations? In the following, the competence of different systems proposed for OOS will be discussed/evaluated to elucidate the position of tethered satellite systems in this domain.

***OOS potential space vehicles:*** close-range rendezvous and target capturing are known as the most challenging part of OOS^45^. While the experience with conventional robots has yielded successes, it has also revealed critical shortcomings^46–49^. Conventional space robots (consisting of a satellite as a base body and one or more robotic arms/manipulators) were suffering from joint backlash, lubricant degradation, time-varying friction, link/arm bending, work-space limitation of the end effector, and motion delay. The effectiveness/performance of robots in assembling very large space structures in orbit is hindered due to their restricted accessibility. Also, precise control of the end effector’s position is particularly challenging due to nonlinearities and the coupling effects between a free-floating base (the satellite) and multiple flexible links. All these factors make it risky to use robotic manipulators for OOS, especially in the face of a non-cooperative or unstable target.

However, the imagination, scientific acumen, courage, and technological expertise of engineers have transcended conventional boundaries, proposing innovative systems that are partially capable of compensating for the limitations inherent in traditional space robots. Tethered space systems represented a seminal concept that paved the way for the realization of various on-orbit servicing missions with optimal cost-efficiency and operational considerations.

***The role of tethered space systems in OOS:*** many researchers have attempted to utilize tethered space systems to achieve the objectives of OOS missions while addressing the shortcomings of conventional space robots. In this context, the tethered space harpoon (TSH) was the first concept proposed as a means of capturing debris and asteroids (for space mining and asteroid analysis). There are valuable studies that have investigated TSH dynamics, shape design, and benefits^50–53^. The tethered space net (TSN) was the second interesting concept for safely capturing tumbling debris or any non-cooperative object. Several exciting papers have reviewed the arrangements, structure, and texture of the net^55^, its dynamic modeling during expansion^56,57^ and also after contact with the target^54,58–60^, and the effect of the initial conditions of the release/launch phase on the behavior and pattern of the net^61,62^, and even bold ideas based on a reusable net to enhance mission cost-effectiveness^63^. The most important difference between TSH/TSN and previous TSSs is the short length of the tether (about 300 m), which mitigates the issues associated with vibrations, fluctuations, unexpected motion, perturbation, and heavy mass. TSH and TSN systems demonstrate exceptional efficacy in capturing space debris and inactive objects from considerable and safe distances^64^. While these systems offer several advantages, they also exhibit notable limitations and weaknesses:

- For example, they cannot be used for some on-orbit servicing missions such as refueling, maintenance, and inspection.
- There is a possibility that the structure or appendages of the target spacecraft may incur damage during the capturing phase.
- In the case of using a space harpoon, the possibility of generating extra space debris will increase.
- The space net launch mechanism is a single-shot mechanism and it does not retrieve; thus, there is only one chance for successful capturing.
- The success of the capture process significantly depends on initial conditions (such as launch velocity, launch direction, and relative distance to the target) and the target’s specifications (such as size, configuration, and motion).

**Fig. 1:**
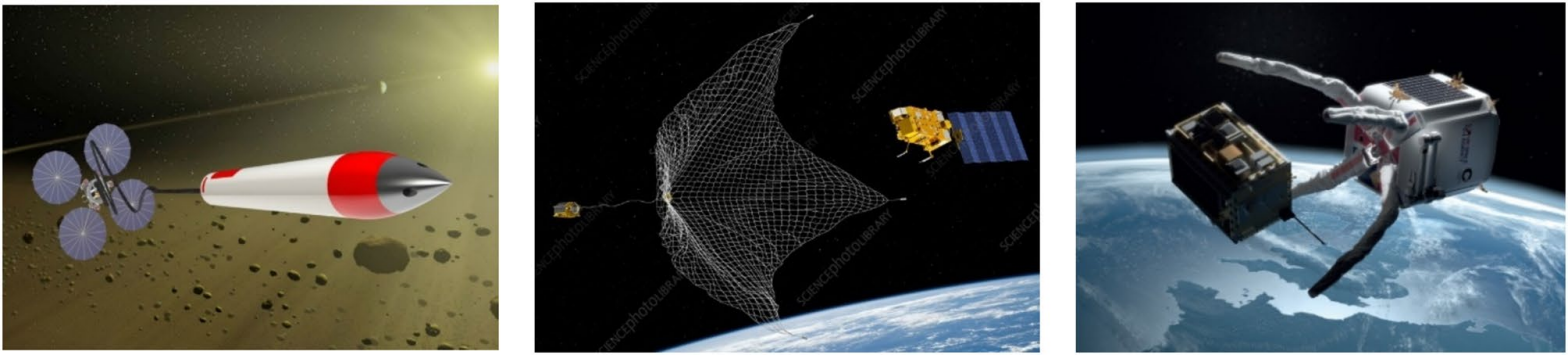
Schematics of a TSH^65^, a TSN^66^, and a conventional space robot^67^.

Figure 1 shows the concept of a TSH, a TSN, and a conventional space robot.

Following the development of TSHs and TSNs, the TSR emerges as the latest advancement in tethered space systems for on-orbit servicing. This innovative tether system demonstrates several promising attributes that effectively address and mitigate the limitations previously associated with TSH and TSN, thereby assuring mission-planners of their reliability.

**Fig. 2:**
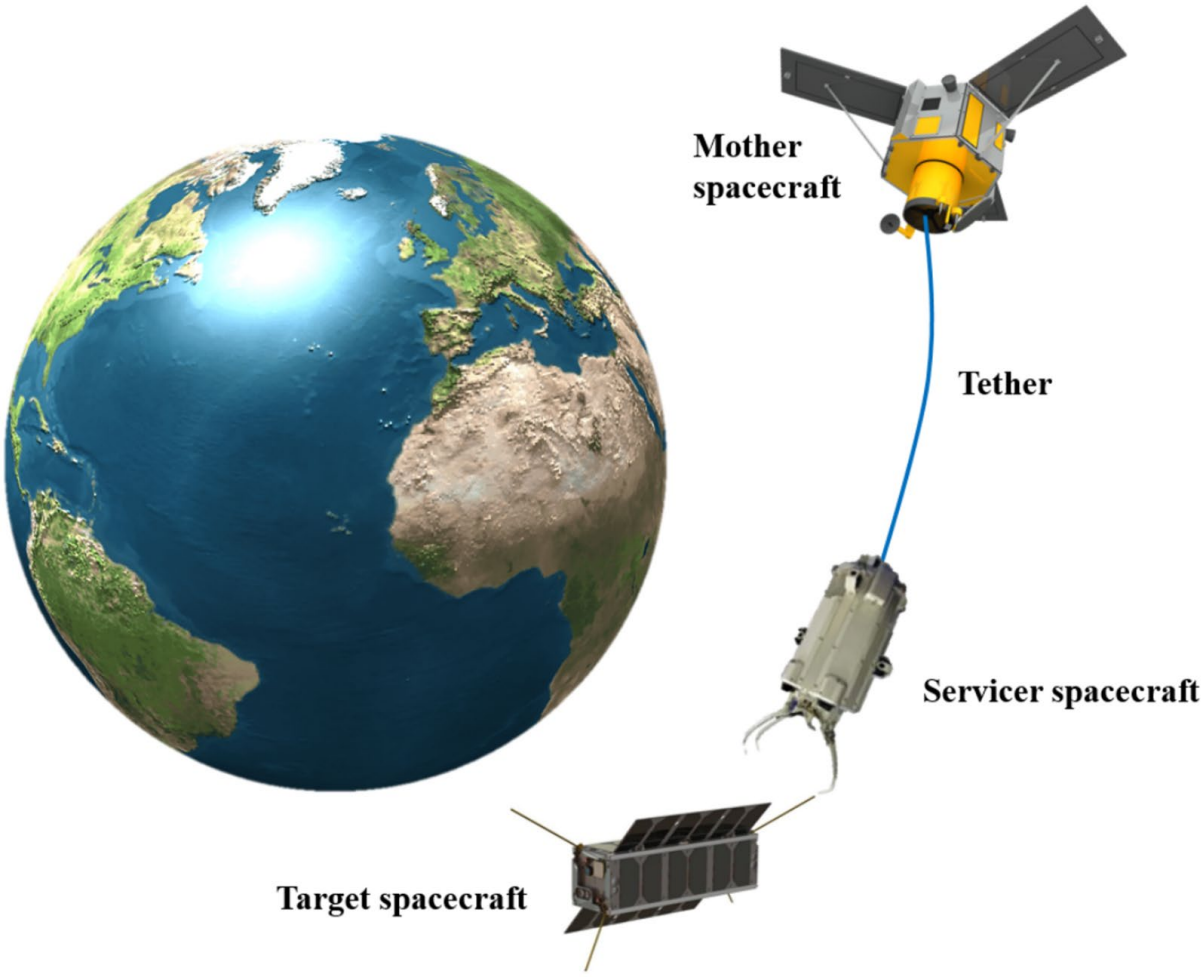
Schematic of a tethered space robot.

***Introduction to the tethered space robot:*** TSRs are the newest application of TSSs, which offer a comprehensive solution for various OOS. TSRs compensate for all the weaknesses of the previous systems (space robot, TSH, and TSN), with lower fuel consumption, enhanced access to more remote points, the capability to undertake various OOS missions due to their high flexibility, and reduced risk of harsh impacts. Indeed, TSRs can be defined as an optimal intersection of TSSs and OOS missions. A typical TSR is a multibody system composed of three parts: a mother spacecraft (also called the platform or main spacecraft), a thin, nonconducting, flexible, and lightweight space tether (tethers can also be used to transmit data and power between the robot and mother spacecraft, if needed), and finally a robot (also called the servicer or daughter spacecraft). The mother spacecraft is a heavy wide-body platform equipped with a big fuel tank (that can act like a space tanker) and all subsystems. The mother spacecraft can control its own position and attitude motion independently. Conversely, the robot is a rigid, agile, and small spacecraft that only consists of essential subsystems. The robot can control its pose after being released from the mother spacecraft. The multi-layer viscoelastic tether has a high length-to-diameter (L/D) ratio and is constructed from strong composite materials (such as Kevlar, carbon nanotubes, graphene, and Nomex) in the inner layers and a taphole in the outer layer (for impact protection). Maybe the greatest advantage of the TSRs, compared to the TSNs and the TSHs, is the robot’s controllability after launch. The tether tension and length are controlled through the mechanism/reel embedded inside the mother spacecraft. Figure 2 provides a schematic representation of a TSR.

**Fig. 3:**
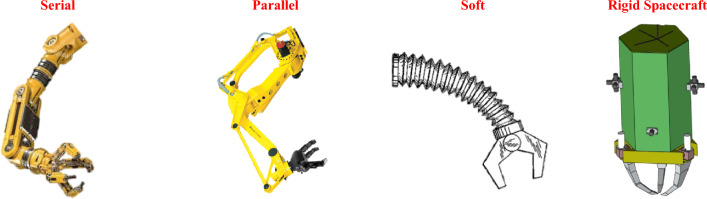
Different robot types.

It should be noted that the robots themselves can have different structures (as shown in Fig. 3). The robot can be a rigid body equipped with only (i) a docking port/interface or (ii) robotic fingers, (iii) a multi-link parallel or serial robotic-arm/manipulator (including several prismatic and revolute joints), (iiii) a soft robot, and so on. Each of these robots has its distinct cons and pros, as well as specific mathematical model. The point is that any small change or innovation can significantly enhance the capabilities of the TSR. For instance, controlling the connection point of the tether to the robot’s body can increase flexibility in its motion, thereby improving maneuverability and operational efficiency.

An OOS utilizing a TSR is divided into five main operational stages:

1. Mother spacecraft maneuvering and station-keeping phase: in this initial stage, the mother spacecraft is positioned at an appropriate distance from the target during an orbit transfer. In this stage, the mother spacecraft and robot are stuck together, and the tether is fully retracted. The mother spacecraft employs its navigation, guidance, and control systems to manage its six degrees of free motion during this phase.
2. Robot deployment phase: in the second stage, the mother spacecraft points the robot towards the target and launches it with a designated/certain initial velocity. Using the initial velocity, the robot/gripper consequently proceeds toward the target while dragging the tether behind it.
3. The third stage focuses on the robot’s approach to the target, which is crucial for minimizing fuel consumption. In this phase, the robot autonomously controls its pose to transition from an initial distance of approximately 100 m to about 15 cm from the target. It should be noted that the robot is equipped with relative navigation systems (such as LIDAR, RADAR, and visual measurement). In this stage, the tether’s deployment rate is controlled by the reel. The tether length control system (reel) ensures that the tensile stress of the tether remains (ignorable) positive during this phase, thereby reducing vibrations and twisting^68,69,71,72^. The tether aids in maintaining the robot’s distance relative to the target and prevent it from getting lost, which is vital for tasks such as inspection, repair, and assembly.
4. Capturing phase: the fourth stage involves capturing the target, a critical phase due to the high risk of impact and potential damage. Various impedance control methods have been proposed for the safe capture of the target^19,73^. Also, coordinated control of tether tension and the robot’s attitude is essential to meet the station-keeping requirements of the robot–target combination.
5. Post-capture and retrieval phase: the final stage pertains to the objectives of the OOS mission which vary based on the target or client demand/need. After servicing, the target is released, and the robot returns to its original position, reattaching to the mother spacecraft via the tether only, which conserves fuel^18,70,74,76^. In a refueling mission, once the target is captured by the robot, the tether is retrieved, and the mother satellite moves and maneuvers towards the target to replenish the target’s fuel tank.

**Fig. 4:**
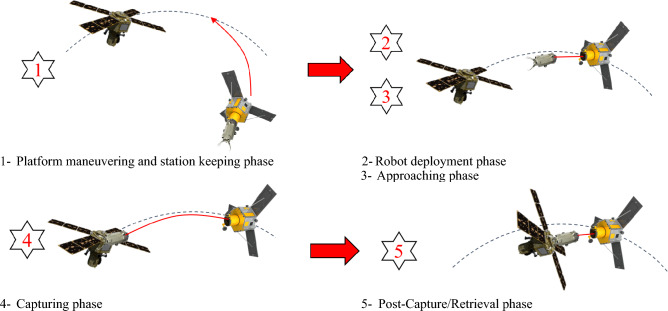
Task flow of a tethered space robot system.

It is worth mentioning that each of the mentioned phases have their distinct sub-phases. Different stages of OOS missions using TSRs are conceptually depicted in Fig. 4.

In summary, it can be concluded that, unlike TSHs and TSNs, TSRs can be used as multi-role OOS platforms that can also safely serve live targets, removing space debris and capturing dead targets.

***TSR dynamic modeling***: as it is clear, a TSR has both rigid and flexible bodies, and this complexity complicates its modeling. Almost all studies have modeled the TSR’s equations of motion using a Lagrange approach. In this area, the tether modeling method is the main difference among studies that have worked on TSR as an integrated dynamic system. The tether is an $N$-degree-of-freedom (DOF) body, and several methods such as the dumbbell model, mass-spring model, multi-element (bead) model, lumped mass model, and absolute nodal coordinate formulation (ANCF) model have been proposed to model such a system. The traditional dumbbell model is the simplest model that converts the TSR into two point-masses connected by a straight, rigid, massless rod. The elasticity and flexibility of the tether are neglected in this method, and the tether length is assumed to be constant. The main advantage of this method is the simplicity of controller design and optimization^77–80^, but it does not express the physical properties and behavior of the tether accurately. In comparison with the dumbbell model, the mass-spring model is a bit more accurate and complex. In this method, both the mother spacecraft and robot are assumed to be point masses, and the tether is replaced by a linear spring that only experiences linear motion^81–83^. The spring can go slack or stretch when the tether length changes from its natural length. This method is also not ideal for modeling a flexible, curved, elastic, and variable-length tether. Low memory occupation, low computational cost, and easy implementation are the main advantages of this method. According to the bead model, the space tether is divided into a series of discrete elements, and each element is composed of elastic rigid string that is bounded by massless revolute joints. In this model, the mass, elasticity, variable length, and flexibility of the tether all can be considered^82^. A solid theoretical foundation and more realistic results are the main advantages of this method. The equivalent continuous model is governed by partial differential equations (PDEs) whose analytical (closed form) solution, if it exists, is quite complex. On the other hand, well-known numerical methods such as the Ritz and Galerkin method are time-consuming and sometimes show low efficiency^84^. The idea of a lumped mass model is to consider the continuous tether as a series of discrete mass points connected by nonlinear spring–dampers^85^. The spring–damper element nonlinearity is due to the ability of the springs to withstand tensile force and not compression. Fortunately, the end body can be modeled as both a point mass and a rigid body. High accuracy and real results are the main advantages of this method. It is clear that increasing the element numbers will result in a more realistic tether, but the computation burden and time will increase subsequently^86^. Also, the system will turn to a multibody system with large degrees of freedom (DoF) that is too complex for analyzing. (Please see^87^ for more information about different modeling methods.)

**Fig. 5:**
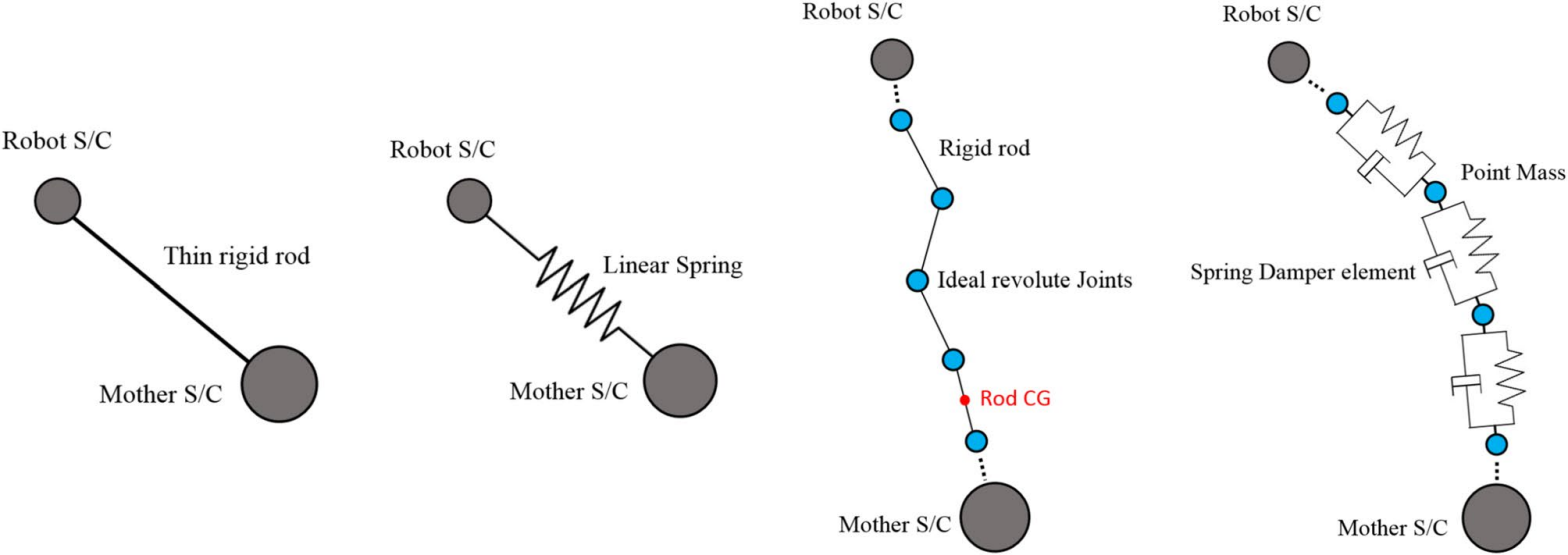
Different modeling method concepts.

Figure 5 shows a schematic of elements and different modeling concepts of a tether.

***ANCF method:*** another interesting modeling method for a flexible tether is the absolute nodal coordinate formulation (ANCF) method presented by Shabana in 1998^88^ for the first time. ANCF originates from the finite element method that brings some advantages such as high precision (specially for highly curved beams or tethers), low computational cost, and simplicity^89^ which makes it more suitable for expressing tether motion behavior in various environments, including terrestrial^75^, subsurface^90,91^, aerial^92,93^, and space. Furthermore, produces accurate results with fewer cable segments compared to the bead model or lumped mass model^94^. Only a few studies have applied this method to TSS dynamics. Tang et al.^95^ investigated the dynamics of variable-length tethers in a satellite deployment mission under zero gravity based on the ANCF method. They presented an anti-slack control strategy during tether deployment that uses (i) tether length and tension, (ii) satellite thrusters, and (iii) reaction wheels together. Shan et al.^96^ investigated the deployment dynamics of the tethered space net and analyzed the sensitivity of critical parameters in a debris-capturing mission. They concluded that an ANCF model is capable of describing the flexibility and deformation between two consecutive nodes on the net precisely. Reference^89^ analyzed the dynamic behavior of a TSS for space debris capture in 2D. The tether’s mass, elasticity, and deformation have been modeled accurately, but the end bodies and the target were treated as point masses. In this study, it was assumed that the tether is fully opened/deployed and the length does not change, resulting in a fixed mass matrix in the motion equations. The comparison of the ANCF and dumbbell modeling methods in describing the tether’s motion is another valuable contribution of their work. The dynamic responses of the proposed tethered space system after debris capture have been analyzed under different impact angles, impact velocities, and tether lengths. A significant contribution was made by Luo et al.^97^, who investigated the dynamics of a spinning three-body tethered satellite formation in 2D. Both large displacements and large deformations of the variable-length tethers were described using the ANCF method in the arbitrary Lagrange–Euler (ANCF-ALE) framework. ANCF-ALE allows for mesh movement independent of material motion, which is beneficial for problems involving moving boundaries or variable mass. In this work it was assumed that the tether is initially completely wrapped/closed and is then gradually deployed. Thus, the deployed tether length and mass are time-varying parameters. In this case the mass matrix is not fixed/constant with a known size, and the attitude motion of the end bodies is ignored, and also there is no coupling between tether dynamics and rigid sub-satellite motion. Zhang^98^ analyzed the tether deployment dynamics under a gravity gradient and Coriolis forces. In this paper, the deployment velocity has been controlled to prevent rebound and minimize tether deflection/towing in the station-keeping phase. The aforementioned studies have only dealt with the problem of analyzing the behavior and motion of the tether itself using the ANCF modeling method, and the navigation, guidance, and control of the robot were not issues for them. On the other hand, in the references that deal with rendezvous and docking of the servicer spacecraft with the target^18,70,72,77^, the effects of the tether’s dynamics—curvature, fluctuations, elasticity, and flexibility—on the servicer’s pose motion have not been properly investigated. Indeed, the tether is considered only as a brake force vector in those studies.

As additional information, it is appropriate to introduce the attractive method called Nodal Position Finite Element Method (NPFEM), which is known as a subset of finite element methods and is especially suitable for modeling flexible bodies, but has been utilized less frequently. NPFEM focuses on the positions of nodes in the mesh to improve accuracy and convergence in simulations. NPFEM enhances the representation of complex geometries and material behaviors, leading to more accurate results, especially in nonlinear analyses^99,100^. In contrast to traditional FEMs that rely on nodal displacements, the new NPFEM uses nodal positions as basic variables to eliminate the need to decouple elastic deformation from rigid-body motion. As a result, it can avoid the accumulated errors arising from the existing FEM over a long period of time by comparing the deformed element with its undeformed status directly. This approach is particularly useful in the dynamic modeling of mechanical systems, where the current positions of components are more relevant to designers than displacements. As a review of the relevant references related to NPFEM that have been used in the space engineering field, Ref.^101^ can be mentioned. Li and Zhu used the nodal position finite element method for dynamic analysis of an electrodynamic tether system, while aerodynamic drag force, gravity force, and electrodynamic force were considered. The simulation results indicate that NPFEM is suitable for long-term simulations, demonstrating stability, accuracy, and the absence of divergence or singularity^101^. Additionally, they explored this method for the long-term deorbiting process of tethered spacecraft in other work^102^. This study proposes a globally stable numerical approach that integrates NPFEM with implicit, Symplicit, two-stage, and fourth-order Gaussian–Legendre Runge–Kutta time integration methods. Dynamic modeling of a cable-towed body using NPFEM is presented in^103^. The cable has been modeled and analyzed using a new nodal position finite element method, which calculates the position of the cable directly rather than the displacement by the existing finite element method. The new NPFEM designed in^103^ eliminates the need to decouple rigid-body motion from total motion, addressing numerical errors and the limitations of small rotations at each time step present in existing nonlinear finite element methods. *Zhu* and *Pour* developed a new nodal position finite element method (NPFEM) as an alternative to the existing finite element method (FEM) for plane (2D) elastic problems^104^. To analyze dynamic problems involving significant changes in mass and tether length, NPFEM should be employed within the Arbitrary Lagrangian–Eulerian (ALE) framework. Li and Zhu^105^ utilized NPFEM within an ALE framework to develop a high-fidelity and high-accuracy model of a new partial space elevator equipped with parallel tethers and multiple climbers. They also investigated the liberation suppression of a partial space elevator caused by a moving climber through tether deployment and/or retrieval at main and sub-satellites. The analysis was conducted based on the equations of motion achieved based on NPFEF-ALE method. In this work, the variable-length approach was used to describe the movement of the climber along the tether and the deployment and/or retrieval of the tether at the end satellites^106^. This paper proposes a modeling method for non-equatorial space elevators utilizing a nodal-position finite element method (NPFEM) extended to a rotational coordinate system. Indeed, we can see a new format of NPFEM in a non-inertial coordinate system in this work, facilitating three-dimensional analysis of a non-equatorial space elevator. After determining the equilibrium position of the NPFEM, Kuzuno et al. analyzed the dynamic response of the tether during climber ascent^107^. Li et al. investigated the stability and control of radial deployment of an electric solar wind sail, a multibody dynamic system, while considering high-order modes of elastic tethers^108^. In this work, the tether deployment process was modeled by the NPFEF-ALE.

***Research gap and contribution:*** to the best of the authors’ knowledge, among the articles that chose the Lagrange and ANCF method for modeling tethered space robots, no research has considered the robot as an individual active and controllable spacecraft. Thus, no research has been found that deals with the design of the controller for the tethered robot, while also considering the effects of forces and moments arising from the tether’s interaction with the robot. Also, no references are found that have considered both the rotational and translational motion of the robot with the tether in the equations of motion of the whole integrated system. Therefore, modeling a TSR using the ANCF technique, where the robot acts as an active spacecraft capable of pose motion control, is a valuable research area.

In this paper we present the TSR as an exceptionally efficient and highly viable system for an on-orbit refueling mission. The main goal of this paper is to model a TSR using the ANCF method in close-range rendezvous (approaching phase) with a target orbiting in a Keplerian circular orbit, while the robot itself is an active spacecraft. In this regard, the coupled relative pose motion equations of the robot and target spacecraft will serve to generate the desired paths. Then, the robot’s attitude and position controllers will produce enough torque and force to meet the rendezvous requirements. The robot will sync with the target and move towards it while towing the tether. In other words, the dynamic coupling of the robot and tether motion (tether tensile force) is taken into account.

This paper should be compared only with works dealing with modeling and control of TSRs based on the combination of the Lagrange equation and ANCF method in the approaching phase, and the main contributions of this paper are summarized as follows:

1. The TSR is considered and modeled as a combination of a flexible, rigid body and a point mass based on the Lagrange equation and ANCF technique, while the robot actively controls its attitude and position simultaneously based on relative navigation data and towing the tether behind itself.
2. The robot’s and tether’s motions (both translational and orientational) are coupled, and the interaction of their motion is taken into account in the equations of motion. Indeed, the relative distance between the attachment point of the tether and the center of mass of the robot is accounted for (see Fig. 7). As a result, the tension force exerted by the tether generates torque around the center of mass of the robot. Conversely, the rotation of the robot around its center of mass induces a tension force in the tether.
3. Design of a sliding mode controller based on the tethered space robot dynamics expressed in an ANCF framework.

***Paper structure:*** Section "Dynamic modelling of a tethered space robot" presents a detailed description of the mission scenario, coordinate systems, essential definitions, assumptions, and the dynamic modeling of the TSR. This section concludes with the derivation of the relative pose motion between the chaser robot and the target spacecraft, which serves as a key prerequisite for generating the robot’s trajectory. Section "Numerical simulation" introduces the input data used for the numerical simulations, followed by the validation of the developed models and codes, and provides a comprehensive analysis of the simulation results. Finally, Section "Conclusions" summarizes the main conclusions of the study.

## Dynamic modelling of a tethered space robot

### Mission description

One of the most meaningful and economic OOS missions is related to fuel supply. For this purpose, first, a fuel tank bank should be created in the orbit (for example, a warehouse consisting of 50 fuel tanks with a certain capacity) that then fuels the client’s satellites with the help of these stored fuel tanks.

**Fig. 6:**
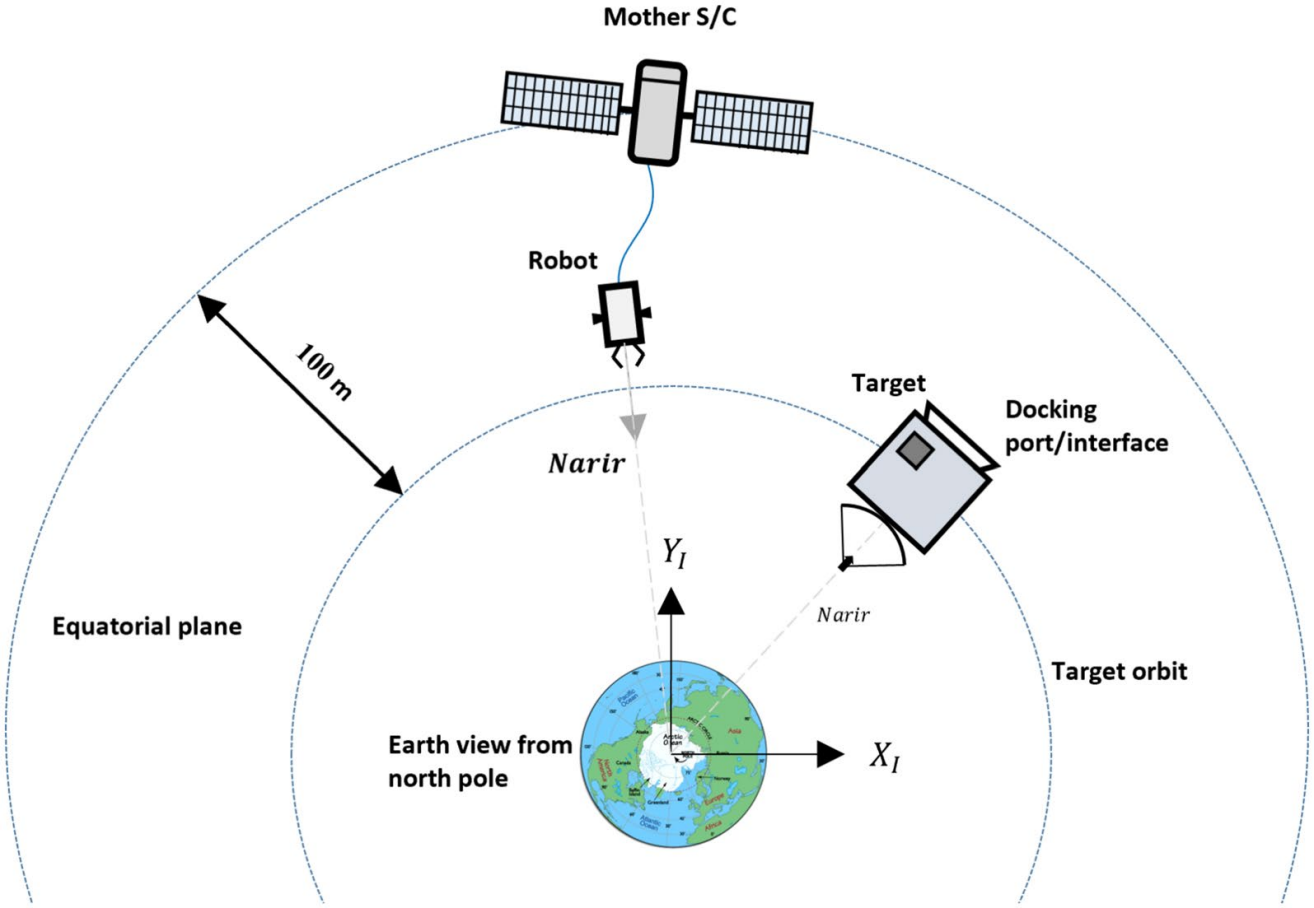
Mission scenario.

Thus, we focused on the approaching phase of a TSR to a telecommunication spacecraft, which is about to run out of fuel. The robot can freely translate and rotate in space as a rigid spacecraft, while the tether is slightly extensible. The point where the tether is attached to the robot is displaced from its center of mass. Thus, the robot’s dynamics is coupled with the tether’s deformations and displacements. We assumed that the tether is completely deployed, and the robot needs to move towards a target that is a few meters (about 10 m) away from it. The target is assumed to be cooperative, allowing it to maintain its attitude during the docking phase. The target is equipped with a docking port/interface, and nadir pointing is the suitable condition/attitude for safe docking. The target and robot are orbiting in LEO with a height difference of about 100 m. The robot is equipped with reaction thrusters for both attitude and position control, whereas the target relies solely on reaction wheels for attitude control. As shown in Fig. 6, the target and robot have different (but close) true anomalies, and the robot is responsible for nullifying the relative distance error. Figure 6 illustrates the mission scenario.

**Fig. 7:**
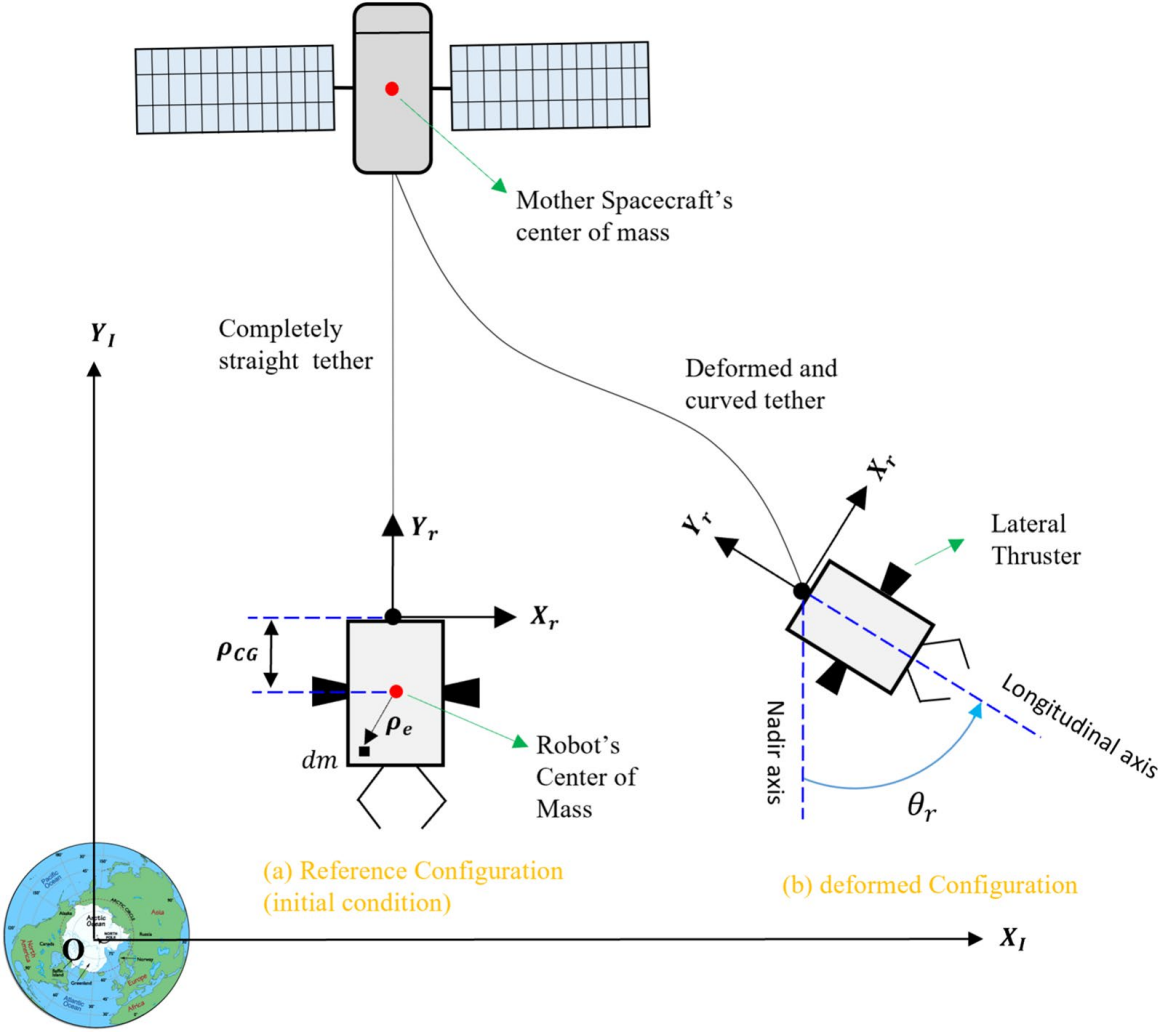
Mission concept.

As shown in Fig. 7, the tether is entirely straight and aligned with the local gravity gradient vector in an initial condition (this alignment can be achieved by finely tuning the negligible positive tension within the tether and controlling its deployment rate in practice). The robot can move freely and tow the tether; consequently, the tether may undergo deformation during the approaching phase. As demonstrated in Fig. 7, the robot’s center of mass is positioned at a distance $\left|{{\boldsymbol{\rho}}}_{{\boldsymbol{C}}{\boldsymbol{G}}}\right|$ below the connection point of the tether to the robot structure. Figure 7 shows the TSR in an initial condition and after deformation. ${{\boldsymbol{\rho}}}_{{\boldsymbol{e}}}\epsilon {\mathfrak{R}}^{2}$ in Fig. 7 refers to the vector from the mass center of the robot to a mass element represented in the robot body fixed frame.

Some essential coordinate frames should be defined for mathematical modeling. We choose an inertial global reference frame (located on the Earth), body-fixed frame, and RSW frame to explain the motion of the target and TSR in an OOS mission.

- The origin of the global reference frame is on the Earth’s center of mass, ${{\boldsymbol{X}}}_{{\boldsymbol{I}}}$ is pointing along the vernal equinox, and ${{\boldsymbol{Y}}}_{{\boldsymbol{I}}}$ is normal to ${{\boldsymbol{X}}}_{{\boldsymbol{I}}}$ (counter clockwise orientation).
- The origin of the robot body-fixed frame is located at the end of the tether, where the tether is attached to the robot. ${{\boldsymbol{Y}}}_{{\boldsymbol{r}}}$ is along the longitudinal axis, but against its direction. ${{\boldsymbol{X}}}_{{\boldsymbol{r}}}$ is normal to the robot’s longitudinal axis and points to the left side. Thus, ${\theta}_{r}=-{90}^{^\circ }$ denotes the nadir pointing attitude for the robot.
- The origin of the target body-fixed frame is located on the target’s center of mass. ${{\boldsymbol{X}}}_{{\boldsymbol{t}}}$ is normal and toward the docking port (along the target’s longitudinal axis but against its direction), and ${{\boldsymbol{Y}}}_{{\boldsymbol{t}}}$ is normal to ${{\boldsymbol{X}}}_{{\boldsymbol{t}}}$ and points to the right side (counter clockwise). Thus, ${\theta}_{t}={0}^{^\circ }$ denotes the nadir pointing attitude for the target spacecraft.
- The origin of the RSW frame is located on target spacecraft, ${{\boldsymbol{X}}}_{{\boldsymbol{r}}{\boldsymbol{s}}{\boldsymbol{w}}}$ is always pointing from Earth along the radius vector toward the spacecraft, and ${{\boldsymbol{Y}}}_{{\boldsymbol{r}}{\boldsymbol{s}}{\boldsymbol{w}}}$ is perpendicular to ${{\boldsymbol{X}}}_{{\boldsymbol{r}}{\boldsymbol{s}}{\boldsymbol{w}}}$ and is in the direction of orbital velocity.

**Fig. 8:**
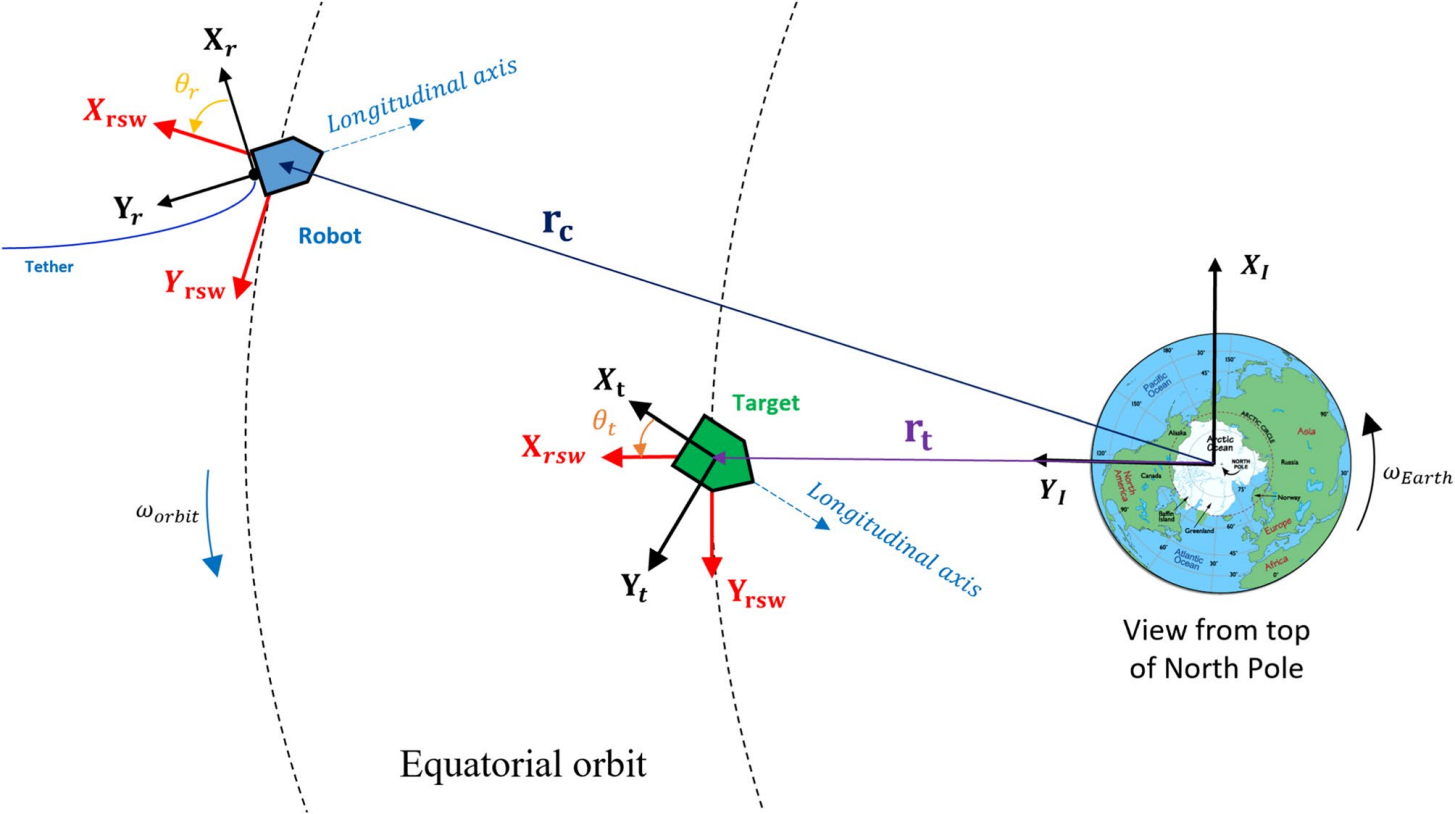
Schematic of different frames.

Figure 8 shows different introduced frames.

According to Fig. 8, the rotation matrix from target and robot body-fixed frames to RSW frame is:

1 $${{\boldsymbol{R}}}_{{\boldsymbol{r}}}^{{\boldsymbol{r}}{\boldsymbol{s}}{\boldsymbol{w}}}=\left[\begin{array}{cc}\mathrm{cos}{\theta}_{r}& -\mathrm{sin}{\theta}_{r}\\ \mathrm{sin}{\theta}_{r}& \mathrm{cos}{\theta}_{r}\end{array}\right],\quad {{\boldsymbol{R}}}_{{\boldsymbol{t}}}^{{\boldsymbol{r}}{\boldsymbol{s}}{\boldsymbol{w}}}=\left[\begin{array}{cc}\mathrm{cos}{\theta}_{t}& -\mathrm{sin}{\theta}_{t}\\ \mathrm{sin}{\theta}_{t}& \mathrm{cos}{\theta}_{t}\end{array}\right]$$

where ${\theta}_{t}$ and ${\theta}_{r}$ are the attitude angles of the target and the robot, respectively.

According to what is shown in Fig. 8, for the target and robot satellites, “${\theta}_{t}=0^\circ$” and “${\theta}_{r}=-90^\circ$” are nadir situations.

### Assumptions

The dynamics of a multibody rigid–flexible combinative system are too complex to allow for the presentation of a complete and accurate model with all details. To model and analyze such systems, we must consider the following simplifying assumptions:

1. As mentioned earlier, the mother spacecraft is much heavier and bigger than the tether and the robot. Therefore, it can be assumed that the motions of the robot and tether have no effect on the position and motion of the mother spacecraft. Thus, the mother spacecraft can be treated as a point mass that is orbiting in a LEO circular orbit.
2. The mass center of the whole system is assumed to coincide with the mass center of the mother spacecraft, which moves in a circular Keplerian orbit.
3. The mother spacecraft, robot, and target are assumed to be in the same orbit plane. Thus, the robot only needs some on-orbit-plane maneuvers/motion, and the out-of-plane motion of the tether can be neglected.
4. The cross-section of the slender tether is circular and totally rigid/stiff.
5. The mass distribution of the tether along its length is homogeneous and uniform.
6. Since the length of the space tether (about 100 m) is far greater than the cross-sectional diameter (1.6 mm), its torsion and associated inertia can be neglected.
7. The tether is a flexible cable that does not tolerate any pressure force or stress. The circular cross-section of the slender tether is stiff (it does not undergo shape change) and is assumed to be always perpendicular to the deformed axis of the tether during the motion. Thus, we assumed that the tether’s “lateral stretch” is also negligible.

### Dynamic modeling

**Fig. 9:**
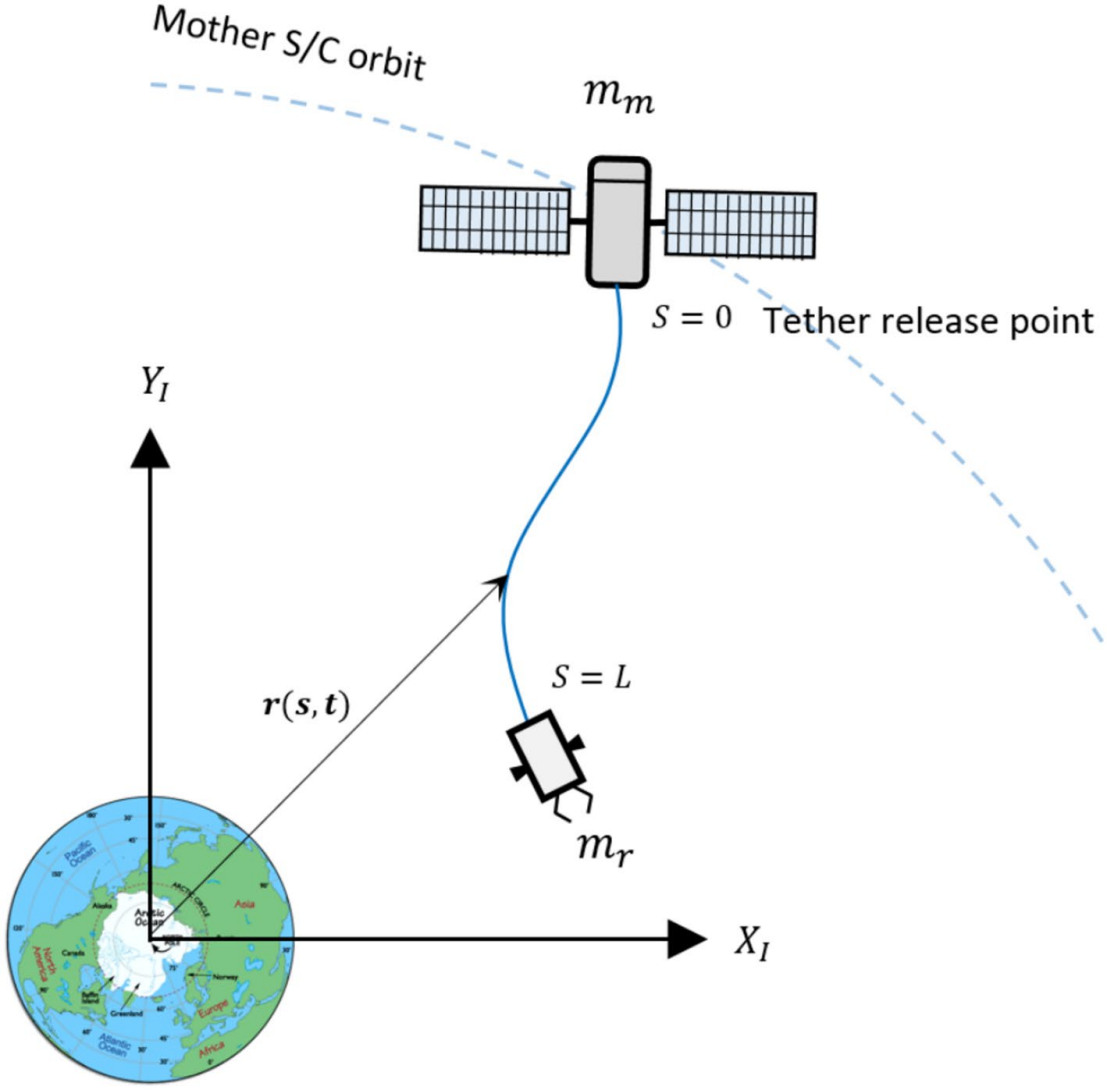
Continuous model of the TSR.

In this section, we will present a dynamic model of the TSR based on the ANCF method considering a large deformation of the tether. As shown in Fig. 9, ${m}_{m}$ is considered as the mother spacecraft’s mass, ${m}_{r}$ as the robot’s mass, and $L$ as the total length of un-stretched tether. The orbital motion of the TSR is occurring in the $X-Y$ plane, and $O$ denotes the Earth’s center of mass. The position of any point of the TSR can be described by vector ${\boldsymbol{r}}\left(s,t\right)$ based on the tether’s arc length $\left(s\right)$ and time $\left(t\right)$ as follows:

2 $${\boldsymbol{r}}=x\left(s,t\right){\boldsymbol{i}}+y\left(s,t\right){\boldsymbol{j}}$$

where ${\boldsymbol{i}}$ and ${\boldsymbol{j}}$ are the unit vectors of the global reference frame in the $X$ and $Y$ directions, respectively. $x\left(s,t\right)$ and $y\left(s,t\right)$ represent the $X$ and $Y$ coordinates of the TSR, respectively. Consequently, the vector ${\boldsymbol{r}}\left(0,t\right)$ indicates the position of the mother spacecraft ($s=0$) that we will show it by symbol ${{\boldsymbol{r}}}_{{\boldsymbol{m}}}$. Similarly, ${\boldsymbol{r}}\left(L,t\right)$ indicates the position vector of the robot ($s=L$), that we will show it by symbol ${{\boldsymbol{r}}}_{{\boldsymbol{r}}}$, at time $t$.

Note that $S$ is calculating from tether’s release point towards to the robot.

**Fig. 10:**
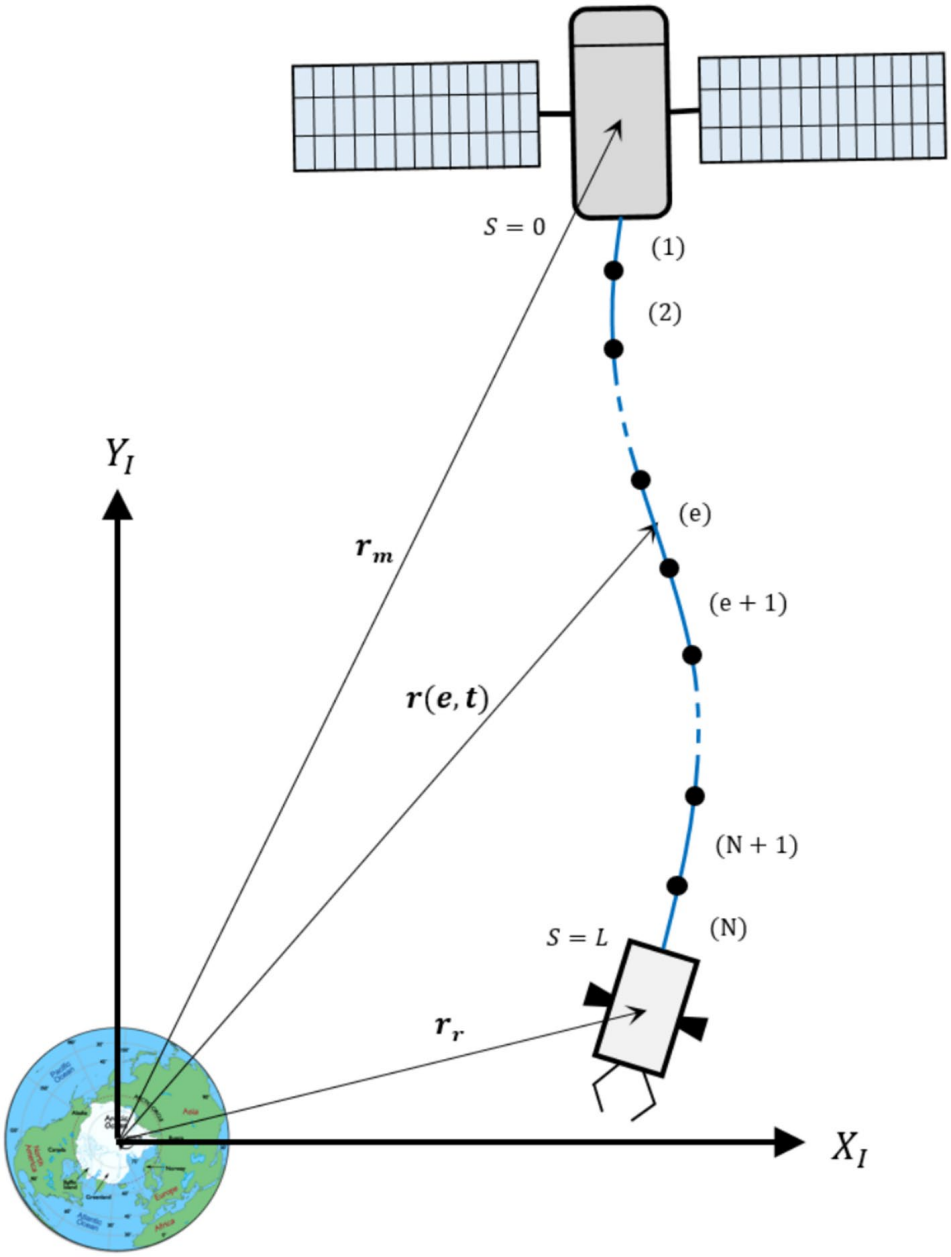
Discretized model of the TSR.

As shown in Fig. 10, the tether consists of $N$ discrete elements and $n=N+1$ nodes. Thus, element $\left(e\right)$ is between node $e$ and $e+1$.

In the case of three elements (see Appendix E), the system involves a total of nine states, with eight of them pertaining to translational motion. These include the mother satellite (first node), the nodes at the start and end of the second element (two nodes on the tether), and the robot (last node). The remaining state is associated with the rotational motion of the robot (one attitude angle).

Translational motion modeling will be covered first, followed by rotational motion modeling. Finally, the problem can be numerically solved by integrating the equations into a matrix-based system of equations.

The tethered satellite system’s equations of motion are determined using Lagrange’s equations. In this regard we need to calculate the kinetic and potential energy of the whole system. The kinetic energy $K$ of the TSR is defined as the sum of the kinetic energies of the mother spacecraft, robot, and tether.

Note that $N$ represents the total number of elements, and $n$ represents the number of nodes. For this case study, in which all nodes experience in-plane motion and only the robot has attitude motion capability, the total degrees of freedom of the system is equal to $2(N + 1) + 1$ (see Appendix E).

The kinetic energy of the mother spacecraft and tether are given as follows^64,109^:

3 $${K}_{m}=\frac{1}{2}{\dot{{\boldsymbol{r}}}}_{{\boldsymbol{m}}}^{{\boldsymbol{T}}}{m}_{m}{\dot{{\boldsymbol{r}}}}_{{\boldsymbol{m}}}$$

4 $${K}_{th}=\frac{1}{2}\rho {\int}_{0}^{L}\left({\dot{{\boldsymbol{r}}}}^{{\boldsymbol{T}}}\dot{{\boldsymbol{r}}}\right)ds$$

where $\rho$ denotes the mass per unit length of the tether’s elements. ${\dot{{\boldsymbol{r}}}}_{{\boldsymbol{m}}}$ and $\dot{{\boldsymbol{r}}}$ are the velocity vector of the mother spacecraft and tether’s elements, expressed in global coordinate.

${\boldsymbol{r}}\left(L,t\right)+{{\boldsymbol{R}}}_{{\boldsymbol{r}}}^{{\boldsymbol{g}}}\left({{\boldsymbol{\rho}}}_{{\boldsymbol{C}}{\boldsymbol{G}}}+{{\boldsymbol{\rho}}}_{{\boldsymbol{e}}}\right)$ denotes the location of a distinct mass element of the robot in the global reference frame (see Fig. 7). ${{\boldsymbol{R}}}_{{\boldsymbol{r}}}^{{\boldsymbol{g}}}$ is the rotation matrix from the robot body-fixed frame to the global reference frame. Using the fact that ${\dot{{\boldsymbol{R}}}}_{{\boldsymbol{r}}}^{{\boldsymbol{g}}}={{\boldsymbol{R}}}_{{\boldsymbol{r}}}^{{\boldsymbol{g}}}S\left({\omega}_{r}\right)$, the kinetic energy of the robot can be written as:

5 $${K}_{r}=\frac{1}{2}\oint {\Vert \dot{{\boldsymbol{r}}}\left(L,t\right)+{{\boldsymbol{R}}}_{{\boldsymbol{r}}}^{{\boldsymbol{g}}}S\left({\omega}_{r}\right)\left({{\boldsymbol{\rho}}}_{{\boldsymbol{C}}{\boldsymbol{G}}}+{{\boldsymbol{\rho}}}_{{\boldsymbol{e}}}\right)\Vert }^{2}dm$$

6 $${K}_{r}=\frac{1}{2}{\dot{{\boldsymbol{r}}}}_{{\boldsymbol{r}}}^{{\boldsymbol{T}}}\left(L,t\right){m}_{r}{\dot{{\boldsymbol{r}}}}_{{\boldsymbol{r}}}\left(L,t\right)+{m}_{r}\dot{{\boldsymbol{r}}}\left(L,t\right){{\boldsymbol{R}}}_{{\boldsymbol{r}}}^{{\boldsymbol{g}}}S\left({\omega}_{r}\right){{\boldsymbol{\rho}}}_{{\boldsymbol{C}}{\boldsymbol{G}}}+\frac{1}{2}{{\boldsymbol{\omega}}}_{{\boldsymbol{r}}}^{{\boldsymbol{T}}}{I}_{{r}_{1}}{{\boldsymbol{\omega}}}_{{\boldsymbol{r}}}$$

where ${\dot{{\boldsymbol{r}}}}_{{\boldsymbol{r}}}$, ${I}_{{r}_{1}},$ and ${{\boldsymbol{\omega}}}_{{\boldsymbol{r}}}$ are the robot’s velocity, moment of inertia with respect to the robot’s body fixed frame, and angular velocity respectively.

Note that ${\oint}_{B}{{\boldsymbol{\rho}}}_{{\boldsymbol{e}}}dm=0$ and ${I}_{{r}_{1}}=-{\oint}_{B}{\left(S\left({{\boldsymbol{\rho}}}_{{\boldsymbol{C}}{\boldsymbol{G}}}+{{\boldsymbol{\rho}}}_{{\boldsymbol{e}}}\right)\right)}^{2}dm$.

The total kinetic energy of the TSR is:

7 $${K}_{tot}=\frac{1}{2}{\dot{{\boldsymbol{r}}}}_{{\boldsymbol{m}}}^{{\boldsymbol{T}}}{m}_{m}{\dot{{\boldsymbol{r}}}}_{{\boldsymbol{m}}}+\frac{1}{2}\rho {\int}_{0}^{L}\left({\dot{{\boldsymbol{r}}}}^{{\boldsymbol{T}}}\dot{{\boldsymbol{r}}}\right)ds+\frac{1}{2}{\dot{{\boldsymbol{r}}}}_{{\boldsymbol{r}}}^{{\boldsymbol{T}}}\left(L,t\right){m}_{r}{\dot{{\boldsymbol{r}}}}_{{\boldsymbol{r}}}\left(L,t\right)+{m}_{r}{\dot{{\boldsymbol{r}}}}_{{\boldsymbol{r}}}^{{\boldsymbol{T}}}\left(L,t\right){{\boldsymbol{R}}}_{{\boldsymbol{r}}}^{{\boldsymbol{g}}}S\left({\omega}_{r}\right){{\boldsymbol{\rho}}}_{{\boldsymbol{C}}{\boldsymbol{G}}}+\frac{1}{2}{{\boldsymbol{\omega}}}_{{\boldsymbol{r}}}^{{\boldsymbol{T}}}{I}_{{r}_{1}}{{\boldsymbol{\omega}}}_{{\boldsymbol{r}}}$$

The TSR’s potential energy $U$ consists of the gravitational potential energy ${U}_{g}$ and the elastic strain energy of the tether ${U}_{E}$ that can be calculated as shown in Eqs. (8) and (9), respectively:

8 $${U}_{g}=-\mu \frac{{m}_{m}}{\Vert {{\boldsymbol{r}}}_{{\boldsymbol{m}}}\Vert }-\mu \rho {\int}_{0}^{L}\frac{1}{\Vert {\boldsymbol{r}}\Vert }ds-\mu \frac{{m}_{r}}{\Vert {{\boldsymbol{r}}}_{{\boldsymbol{r}}}\Vert }$$

where $\mu =\mathrm{398,601.2} \left[{\mathrm{k}\mathrm{m}}^{3}{\mathrm{s}}^{-2}\right]$ denotes the standard gravitational parameter.

**Fig. 11:**
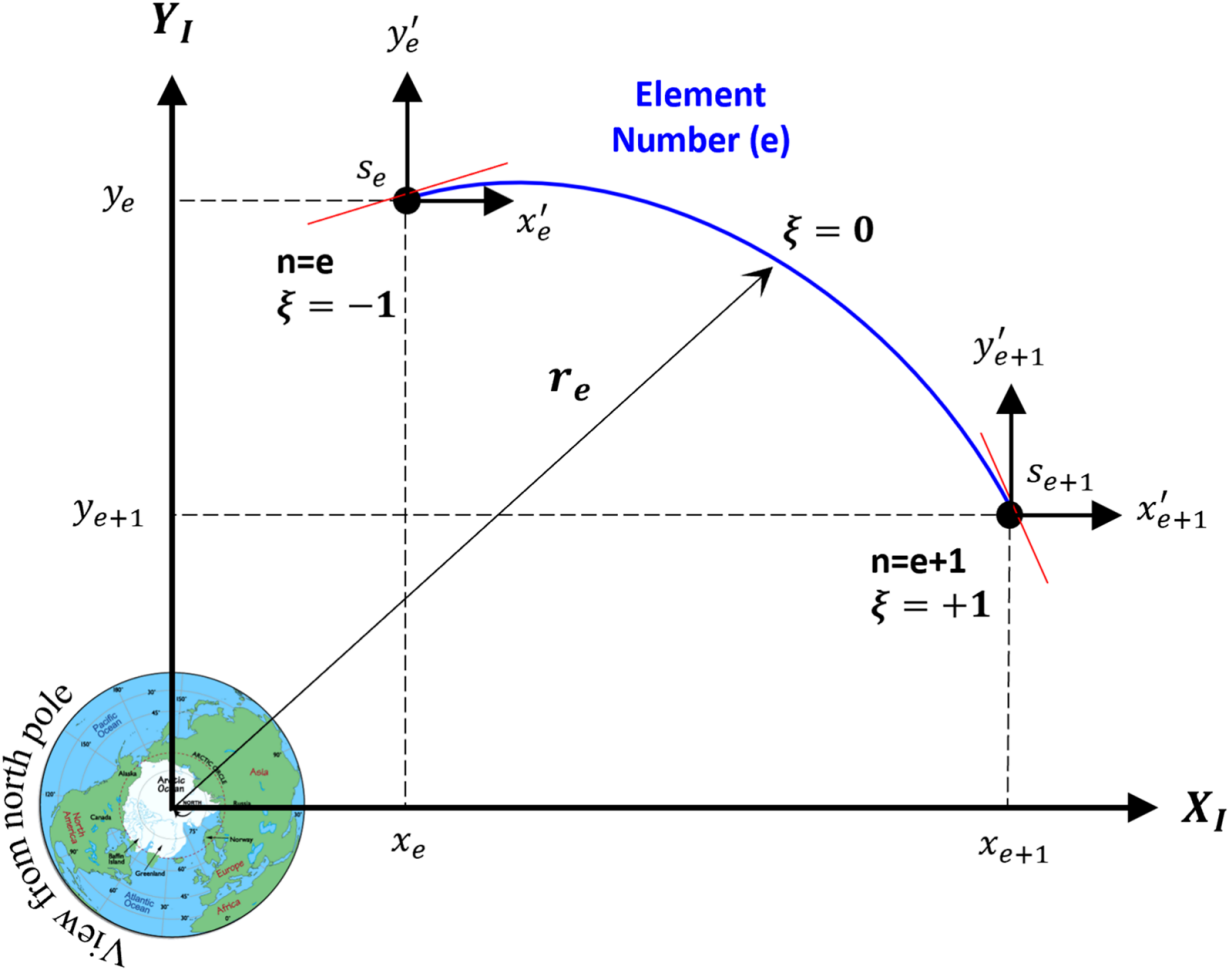
Single element of the tether.

There are two sources of energy for a deformed beam: the strain energy due to longitudinal and bending deformation. Since the tether is very thin and flexible, it does not show strength in response to bending. Thus, only the elastic forces due to longitudinal stretch are considered in this paper. The elastic strain energy ${U}_{E}$ can be calculated as follows:

9 $${U}_{E}=\frac{1}{2}{\int}_{0}^{L}EA{\varepsilon }^{2}ds$$

where $A$ is the tether’s cross-section area and $\varepsilon$ is the axial strain that can be expressed based on “Cauchy–Green” longitudinal strain as follows^110^ (see Appendix D):

10 $$\varepsilon =\frac{1}{2}\left({{\boldsymbol{r}}}^{\prime\boldsymbol{T}}{{\boldsymbol{r}}}^{\prime}-1\right)$$

where ${{\boldsymbol{r}}}^{{\prime}}$ is the derivative of $r$ with respect to $s$. Indeed, ${{\boldsymbol{r}}}^{{\prime}}$ denotes the slope of the tangent line to the tether curve at certain point (see Fig. 11).

Note that $\varepsilon >0$ denotes the tensile stress and $\varepsilon <0$ denotes the pressure stress. Since a thin flexible tether does not withstand compressive stress, therefore, negative values of $\varepsilon$ are replaced by zero.

The stress of an elastic body under longitudinal deformation is given as:

11 $$\sigma =E\left(\varepsilon +\alpha \dot{\varepsilon }\right)$$

where $E$ is known as Young’s modulus (related to the tethers material) and $\alpha$ is the internal damping coefficient of viscoelastic materials.

An element of the discretized tether model (element number $e$ for example) is shown in Fig. 11. This figure will be useful for justification of deriving the tether’s discretized equations of motion.

In the ANCF method, the boundary nodal points of the tether element in 2D space is defining by (i) the position vector (for example $\left({x}_{e} , {y}_{e}\right)$ and $\left({x}_{e+1} , {y}_{e+1}\right)$ in Fig. 11) and (ii) the slope vector (for example $\left({x}_{e}^{{\prime}} , {y}_{e}^{{\prime}}\right)$ and $\left({x}_{e+1}^{{\prime}} , { y}_{e+1}^{{\prime}}\right)$ in Fig. 11). The positions and slopes of the two boundary nodes in Fig. 11 are used as the generalized coordinates of the tether element. Based on the Hermite interpolation method, the coordinates of any arbitrary point on the element (e) can be calculated by ${{\boldsymbol{r}}}_{{\boldsymbol{e}}}, {{\boldsymbol{r}}}_{{\boldsymbol{e}}+1}, {{\boldsymbol{r}}}_{{\boldsymbol{e}}}^{{\prime}}, \mathrm{a}\mathrm{n}\mathrm{d} {{\boldsymbol{r}}}_{{\boldsymbol{e}}+1}^{{\prime}}$ as follows:

12 $${\boldsymbol{r}}\left(s,t\right)={\boldsymbol{N}}\left(s\right){{\boldsymbol{X}}}_{{\boldsymbol{e}}}\left(t\right) \to for\quad {S}_{e}\le S\le {S}_{e+1}$$

where ${\boldsymbol{N}}\left(s\right)$ and ${{\boldsymbol{X}}}_{{\boldsymbol{e}}}\left(t\right)$ are the shape function (that is extracted based on a cubic spline interpolation function and also is an isoparametric function), and the nodal coordinate vector is given as follows:

13 $${\boldsymbol{N}}\left(s\right)=\left[{N}_{1}{\boldsymbol{I}},{N}_{2}{\boldsymbol{I}},{N}_{3}{\boldsymbol{I}},{N}_{4}{\boldsymbol{I}}\right]$$

14 $${{\boldsymbol{X}}}_{{\boldsymbol{e}}}\left(t\right)={\left[{x}_{e}, {y}_{e}, {x}_{e}^{\prime}, {y}_{e}^{\prime} ,{x}_{e+1}, {y}_{e+1}, {x}_{e+1}^{\prime}, {y}_{e+1}^{\prime}\right]}^{T}$$

where ${\boldsymbol{I}}$ is an identity matrix of order 2, and ${\boldsymbol{N}}\left(s\right)$ is a $2\times 8$ matrix.

Substituting ${\boldsymbol{N}}\left(s\right)$ and ${{\boldsymbol{X}}}_{{\boldsymbol{e}}}\left(t\right)$ into Eq. (12) gives:

15 $${\boldsymbol{r}}\left(s,t\right)=\left[\begin{array}{c}{N}_{1}{x}_{e}+{N}_{2}{x}_{e}^{\prime}+{N}_{3}{x}_{e+1}+{N}_{4}{x}_{e+1}^{\prime}\\ {N}_{1}{y}_{e}+{N}_{2}{y}_{e}^{\prime}+{N}_{3}{y}_{e+1}+{N}_{4}{y}_{e+1}^{\prime}\end{array}\right]$$

The suitable ${N}_{i} \left(i=1,\dots , 4\right)$ for a thin tether are polynomials of order 3:

16 $$\begin{aligned} N_{1} & = \frac{1}{4}\left( {\xi - 1} \right)^{2} \left( {\xi + 2} \right)\quad N_{2} = \frac{l}{8}\left( {\xi - 1} \right)^{2} \left( {\xi + 1} \right) \\ N_{3} & = \frac{1}{4}\left( {\xi + 1} \right)^{2} \left( {2 - \xi } \right)\quad N_{4} = \frac{l}{8}\left( {\xi + 1} \right)^{2} \left( {\xi - 1} \right) \\ \end{aligned}$$

where the $\xi$ non-dimensional quantity is given as $\frac{\left(2s-{S}_{e}-{S}_{e+1}\right)}{l}$, and $l={S}_{e+1}-{S}_{e}$ is the element length. Clearly, the shape function is only a function of $\xi$ but not of $t$.

**Fig. 12:**
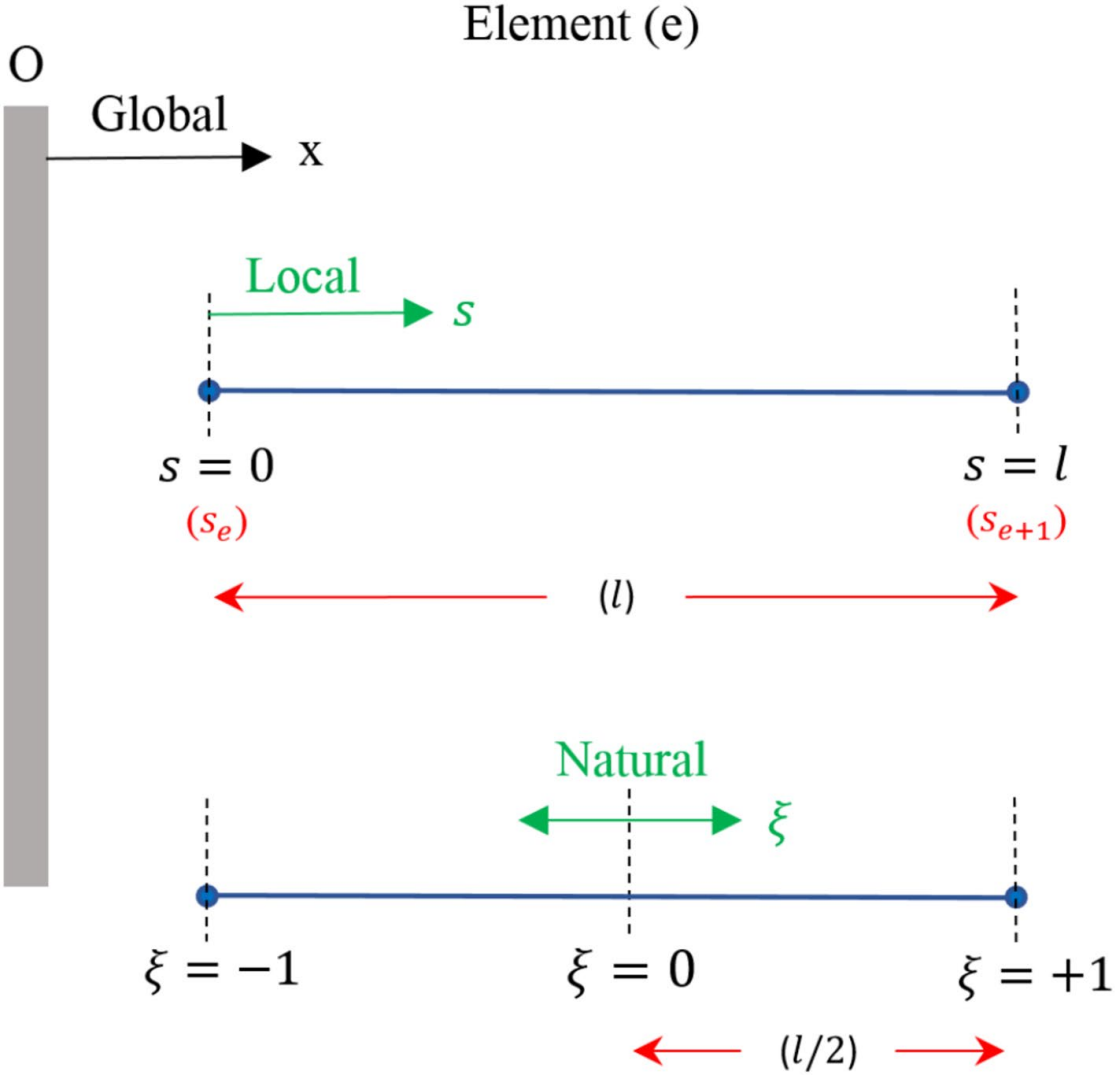
Schematic of global, local, and natural coordinates.

Note that the value of the $\xi$ ranges between $-1$ (for the beginning of the element) and $+1$ (for the end of the element) in the natural coordinate domain. Figure 12 shows the global, local, and natural coordinates. The origin of the global coordinate ($O$) is on the center of the inertia frame. The origins of the (i) local and (ii) natural coordinates are located on the initial node of each element and the middle of the element, respectively.

All the mentioned coordinates are related to each other by a mathematical relationship. Depending on the problem, the designer decides what coordinates to use.

Here, we will use the natural coordinate (which is suitable for a tether with constant length). Thus, for the first element ($e=1$ in Fig. 10), $\xi =-1$ corresponds to the mother spacecraft, and similarly for the last element ($e=N$ in Fig. 10), $\xi =+1$ denotes the robot.

**Fig. 13:**
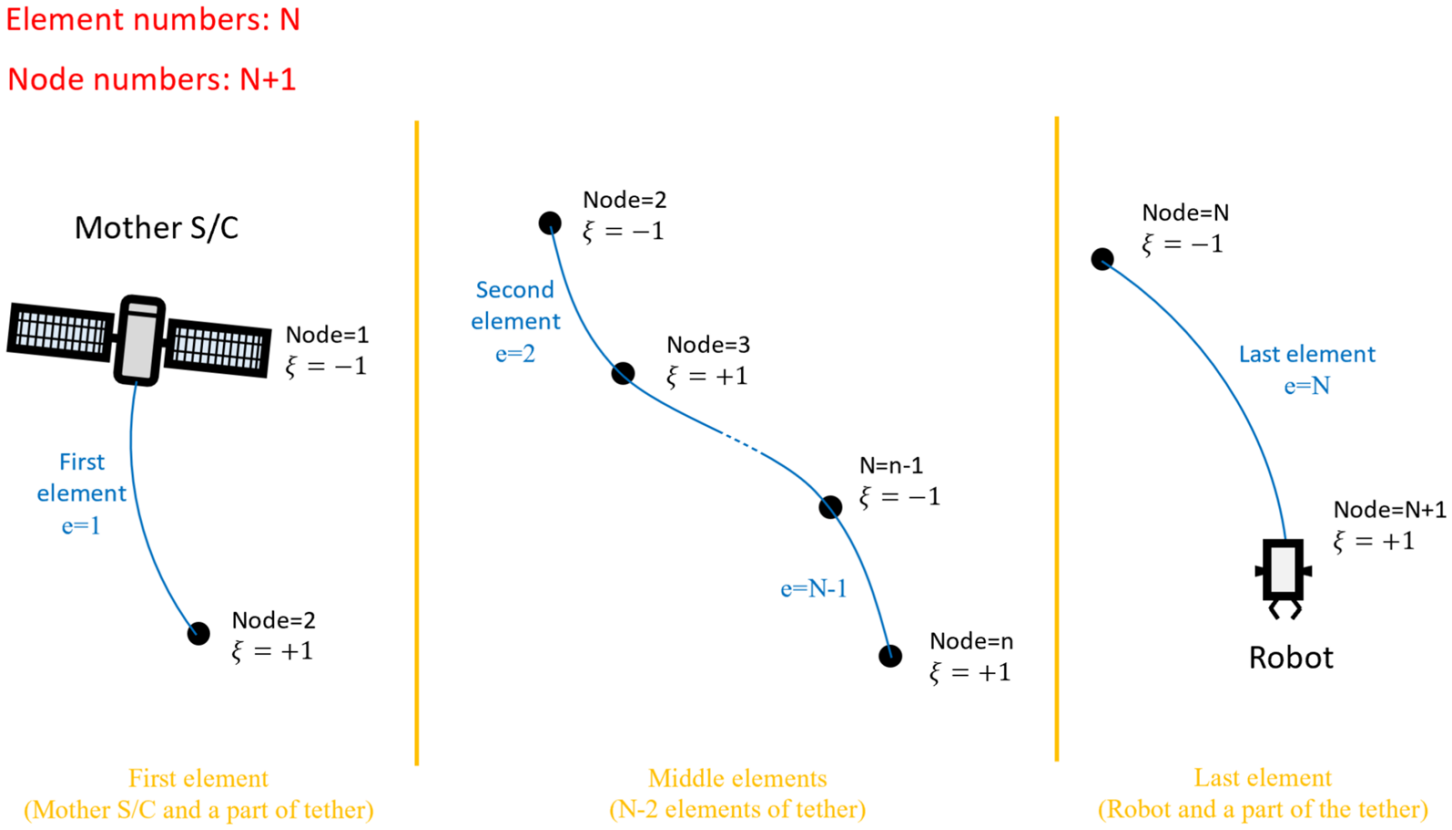
Schematic of nodes and elements of the system.

Figure 13 is presented for a better understanding of the natural coordinates.

The velocity vector of every arbitrary point on the tether elements can be calculated from a time derivative of Eq. (17):

17 $$\dot{{\boldsymbol{r}}}\left(s,t\right)={\boldsymbol{N}}\left(s\right){\dot{{\boldsymbol{X}}}}_{{\boldsymbol{e}}}\left(t\right)$$

Now, the discretized kinematic and potential energy equations can be rewritten based on the shape function and nodal coordinates as follows:

18 $$\begin{aligned}{K}_{tot}&=\sum_{e=1}^{N}\left({\delta}_{e1}\frac{{m}_{m}}{2}{\left[{\dot{{\boldsymbol{X}}}}_{{\boldsymbol{e}}}^{{\boldsymbol{T}}}\left({{\boldsymbol{N}}}^{{\boldsymbol{T}}}{\boldsymbol{N}}\right){\dot{{\boldsymbol{X}}}}_{{\boldsymbol{e}}}\right]}_{@{\boldsymbol{\xi}}=-1}+\frac{l}{4}\rho {\dot{{\boldsymbol{X}}}}_{{\boldsymbol{e}}}^{{\boldsymbol{T}}}\left({\int}_{-1}^{+1}\left({{\boldsymbol{N}}}^{{\boldsymbol{T}}}{\boldsymbol{N}}\right)d\xi \right){\dot{{\boldsymbol{X}}}}_{{\boldsymbol{e}}}^{{\boldsymbol{T}}}+{\delta}_{eN}\frac{{m}_{r}}{2}{\left[{\dot{{\boldsymbol{X}}}}_{{\boldsymbol{e}}}^{{\boldsymbol{T}}}\left({{\boldsymbol{N}}}^{{\boldsymbol{T}}}{\boldsymbol{N}}\right){\dot{{\boldsymbol{X}}}}_{{\boldsymbol{e}}}\right]}_{@{\boldsymbol{\xi}}=+1}+{\delta}_{eN}{m}_{r}{\left[{\boldsymbol{N}}{\dot{{\boldsymbol{X}}}}_{{\boldsymbol{e}}}\right]}_{@{\boldsymbol{\xi}}=+1}^{T}{{\boldsymbol{R}}}_{{\boldsymbol{r}}}^{{\boldsymbol{g}}}S\left({\omega}_{r}\right){{\boldsymbol{\rho}}}_{{\boldsymbol{C}}{\boldsymbol{G}}}\right)\\ &\quad+\frac{1}{2}{{\boldsymbol{\omega}}}_{{\boldsymbol{r}}}^{{\boldsymbol{T}}}{I}_{{r}_{1}}{{\boldsymbol{\omega}}}_{{\boldsymbol{r}}}\end{aligned}$$

19 $$U_{tot} = \sum\limits_{e = 1}^{N} {\left[ {\int\limits_{ - 1}^{ + 1} {\left( { - \frac{1}{2}\frac{\mu l\rho }{{\left( {{\boldsymbol{X}}_{{\boldsymbol{e}}}^{{\boldsymbol{T}}} {\boldsymbol{N}}^{{\boldsymbol{T}}} {\boldsymbol{NX}}_{{\boldsymbol{e}}} } \right)^{1/2} }} + \frac{1}{4}EAl\varepsilon^{2} |_{{@\user2{ \xi } = - 1}} - \delta_{eN} \frac{{\mu m_{r} }}{{\left( {{\boldsymbol{X}}_{{\boldsymbol{e}}}^{{\boldsymbol{T}}} {\boldsymbol{N}}^{{\boldsymbol{T}}} {\boldsymbol{NX}}_{{\boldsymbol{e}}} } \right)^{1/2} }}|_{{@\user2{ \xi } = + 1}} } \right)d\xi - \delta_{e1} \frac{{\mu m_{m} }}{{\left( {{\boldsymbol{X}}_{{\boldsymbol{e}}}^{{\boldsymbol{T}}} {\boldsymbol{N}}^{{\boldsymbol{T}}} {\boldsymbol{NX}}_{{\boldsymbol{e}}} } \right)^{1/2} }}} } \right]}$$

where ${U}_{tot}$ is the summation of ${U}_{E}$ and ${U}_{g}$, and ${\delta}_{ij}$ is defined as:

20 $${\delta}_{ij}=\left\{\begin{array}{ll}1& if\quad i=j\\ 0& if\quad i\ne j\end{array}\right.$$

The tether is too thin, and it is reasonable to assume that it is not affected by drag force. Thus, the external forces acting on cable during mission are the gravity force and force due to robot motion. The virtual work principle serves as the foundation for calculating the generalized forces, and the virtual work of the external force (due to robot thrusters or tether ${{\boldsymbol{F}}}_{ext}$) and the tether’s viscoelastic/damping force (as an internal force) can be formulated as follows:

21 $$\delta W= {{\boldsymbol{F}}}_{ext}\cdot \delta {\boldsymbol{r}}-{\int}_{0}^{L}EA\alpha \dot{\varepsilon }\delta \varepsilon ds$$

22 $$\delta {\boldsymbol{r}}={\boldsymbol{N}}\delta {{\boldsymbol{X}}}_{{\boldsymbol{e}}}$$

where $\delta W$ is the virtual work done by the generalized forces with the virtual displacement of the corresponding generalized coordinate $\delta {\boldsymbol{r}}$.

The virtual work can be expressed using the nodal coordinate as follows:

23 $$\delta W= {{\boldsymbol{N}}}^{T}{{\boldsymbol{F}}}_{ext}\delta {{\boldsymbol{X}}}_{{\boldsymbol{e}}}-\sum_{e=1}^{N}\delta {{\boldsymbol{X}}}_{e}^{T}\left[\frac{EA\alpha l}{2}{\int}_{-1}^{+1}\dot{\varepsilon }{\left(\frac{\partial \varepsilon }{\partial {{\boldsymbol{X}}}_{{\boldsymbol{e}}}}\right)}^{T}d\xi \right]$$

We need two coordinates (${{\boldsymbol{X}}}_{{\boldsymbol{e}}}\, and\, \theta$) to define/explain the motion of TSR. In this case we will have two sets of equations of motion that are coupled (Eqs. (29) and (47)).

Using the Lagrange equation, the dynamic equations of translational motion of the TSR can be obtained as:

24 $$\frac{d}{dt}{\left(\frac{\partial {K}_{tot}}{\partial {\dot{{\boldsymbol{X}}}}_{{\boldsymbol{e}}}}\right)}^{T}-{\left(\frac{\partial {K}_{tot}}{\partial {{\boldsymbol{X}}}_{{\boldsymbol{e}}}}\right)}^{T}+{\left(\frac{\partial {U}_{tot}}{\partial {{\boldsymbol{X}}}_{{\boldsymbol{e}}}}\right)}^{T}={\left(\frac{\delta W}{\delta {{\boldsymbol{X}}}_{{\boldsymbol{e}}}}\right)}^{T}\quad for\quad e=1, 2, \ldots , N$$

25 $$\begin{aligned} \frac{d}{dt}\left( {\frac{{\partial K_{tot} }}{{\partial \dot{\user2{X}}_{{\boldsymbol{e}}} }}} \right)^{T} &= \sum\limits_{e = 1}^{N} {\left( {\delta_{e1} m_{m} {\boldsymbol{N}}^{{\boldsymbol{T}}} {\boldsymbol{N}}|_{{@\user2{ \xi } = - 1}} + \frac{l}{2}\rho \int\limits_{ - 1}^{ + 1} {{\boldsymbol{N}}^{{\boldsymbol{T}}} {\boldsymbol{N}}d\xi + \delta_{eN} m_{r} {\boldsymbol{N}}^{{\boldsymbol{T}}} {\boldsymbol{N}}|_{{@\user2{ \xi } = + 1}} } } \right)} \user2{\ddot{X}}_{e} \\ &\quad+ \delta_{eN} m_{r} \left[ {\boldsymbol{N}} \right]_{{@\user2{ \xi } = + 1}}^{T} {\boldsymbol{R}}_{{\boldsymbol{r}}}^{{\boldsymbol{g}}} S\left( {\dot{\omega}_{r} } \right){\boldsymbol{\rho}}_{{{\boldsymbol{CG}}}} \end{aligned}$$

26 $$\frac{\partial {K}_{tot}}{\partial {{\boldsymbol{X}}}_{{\boldsymbol{e}}}}=0$$

27 $${\left(\frac{\partial {U}_{tot}}{\partial {{\boldsymbol{X}}}_{{\boldsymbol{e}}}}\right)}^{T}=\sum_{e=1}^{N}\left({\int}_{-1}^{1}\frac{\mu \rho l\left({{\boldsymbol{N}}}^{{\boldsymbol{T}}}{\boldsymbol{N}}{{\boldsymbol{X}}}_{{\boldsymbol{e}}}\right)}{{2\left({{\boldsymbol{X}}}_{{\boldsymbol{e}}}^{{\boldsymbol{T}}}{{\boldsymbol{N}}}^{{\boldsymbol{T}}}{\boldsymbol{N}}{{\boldsymbol{X}}}_{{\boldsymbol{e}}}\right)}^\frac{3}{2}}d\xi +{\left.\frac{{\delta}_{e1}\mu {m}_{m}\left({{\boldsymbol{N}}}^{{\boldsymbol{T}}}{\boldsymbol{N}}{{\boldsymbol{X}}}_{{\boldsymbol{e}}}\right)}{{\left({{\boldsymbol{X}}}_{{\boldsymbol{e}}}^{{\boldsymbol{T}}}{{\boldsymbol{N}}}^{{\boldsymbol{T}}}{\boldsymbol{N}}{{\boldsymbol{X}}}_{{\boldsymbol{e}}}\right)}^\frac{3}{2}}\right|}_{\xi =-1}+{\left.\frac{{\delta}_{eN}\mu {m}_{r}\left({{\boldsymbol{N}}}^{{\boldsymbol{T}}}{\boldsymbol{N}}{{\boldsymbol{X}}}_{{\boldsymbol{e}}}\right)}{{\left({{\boldsymbol{X}}}_{{\boldsymbol{e}}}^{{\boldsymbol{T}}}{{\boldsymbol{N}}}^{{\boldsymbol{T}}}{\boldsymbol{N}}{{\boldsymbol{X}}}_{{\boldsymbol{e}}}\right)}^\frac{3}{2}}\right|}_{\xi =1}+\frac{EAl}{2}{\int}_{-1}^{1}\varepsilon \frac{\partial \varepsilon }{\partial {{\boldsymbol{X}}}_{e}}d\xi \right)$$

28 $$\frac{\delta W}{\delta {{\boldsymbol{X}}}_{{\boldsymbol{e}}}}={{\boldsymbol{N}}}^{T}{{\boldsymbol{F}}}_{ext}-\sum_{e=1}^{N}\left[\frac{EA\alpha l}{2}{\int}_{-1}^{+1}\dot{\varepsilon }{\left(\frac{\partial \varepsilon }{\partial {{\boldsymbol{X}}}_{{\boldsymbol{e}}}}\right)}^{T}d\xi \right]$$

where ${{\boldsymbol{X}}}_{{\boldsymbol{e}}}$ is a generalized coordinate defined in Eq. (14).

Substituting Eqs. (25)–(28) into (24) will lead to the following set of differential equations:

29 $$\sum_{e=1}^{N}\left({{\boldsymbol{m}}}_{e}{\ddot{{\boldsymbol{X}}}}_{e}+ {{\boldsymbol{q}}}_{e}^{r}+{{\boldsymbol{q}}}_{e}^{l}+{{\boldsymbol{q}}}_{e}^{d}+{{\boldsymbol{q}}}_{e}^{g}\right)=\sum_{e=1}^{N}{{\boldsymbol{Q}}}_{e}$$

where ${{\boldsymbol{m}}}_{e}$, ${{\boldsymbol{q}}}_{e}^{l}$, ${{\boldsymbol{q}}}_{e}^{d}$, and ${{\boldsymbol{q}}}_{e}^{g}$ are the mass matrix, longitudinal elastic force vector, internal damping force vector, and gravitational force vector, respectively. In fact, $\sum_{1}^{N}{{\boldsymbol{m}}}_{e}$ represents the total mass matrix that includes the mass of the first node (${m}_{m}$), the masses of the intermediate elements (tether’s mass), and the mass of the final node (${m}_{r}$).

30 $${\boldsymbol{m}}_{e} = \delta_{e1} m_{m} {\boldsymbol{N}}^{{\boldsymbol{T}}} {\boldsymbol{N}}|_{{@\user2{ \xi } = - 1}} + \frac{l}{2}\rho \int\limits_{ - 1}^{ + 1} {{\boldsymbol{N}}^{{\boldsymbol{T}}} {\boldsymbol{N}}d\xi + \delta_{eN} m_{r} {\boldsymbol{N}}^{{\boldsymbol{T}}} {\boldsymbol{N}}|_{{@\user2{ \xi } = + 1}} }$$

31 $${{\boldsymbol{q}}}_{e}^{r}={\delta}_{eN}{m}_{r}{\left[{\boldsymbol{N}}\right]}_{@{\boldsymbol{\xi}}=+1}^{T}{{\boldsymbol{R}}}_{{\boldsymbol{r}}}^{{\boldsymbol{g}}}S\left({\dot{\omega }}_{r}\right){{\boldsymbol{\rho}}}_{{\boldsymbol{C}}{\boldsymbol{G}}}$$

32 $${{\boldsymbol{q}}}_{e}^{l}=\frac{EAl}{2}{\int}_{-1}^{+1}\varepsilon \frac{\partial \varepsilon }{\partial {{\boldsymbol{X}}}_{{\boldsymbol{e}}}}d\xi$$

33 $${{\boldsymbol{q}}}_{e}^{d}=\frac{EA\alpha l}{2}{\int}_{-1}^{+1}\left(\frac{\partial \varepsilon }{\partial {{\boldsymbol{X}}}_{{\boldsymbol{e}}}}\right)\dot{\varepsilon }d\xi$$

34 $${{\boldsymbol{q}}}_{e}^{g}=\left[\frac{l}{2}{\int}_{-1}^{+1}\mu \rho \frac{{{\boldsymbol{N}}}^{T}{\boldsymbol{N}}}{{\left[{{\boldsymbol{X}}}_{e}^{T}{{\boldsymbol{N}}}^{T}{\boldsymbol{N}}{{\boldsymbol{X}}}_{e}\right]}^{\raisebox{1ex}{$3$}\!\left/ \!\raisebox{-1ex}{$2$}\right.}}d\xi +{\delta}_{e1}\mu {m}_{m}{\left.\frac{{{\boldsymbol{N}}}^{T}{\boldsymbol{N}}}{{\left[{{\boldsymbol{X}}}_{e}^{T}{{\boldsymbol{N}}}^{T}{\boldsymbol{N}}{{\boldsymbol{X}}}_{e}\right]}^{\raisebox{1ex}{$3$}\!\left/ \!\raisebox{-1ex}{$2$}\right.}}\right|}_{\xi =-1}+{\delta}_{eN}\mu {m}_{r}{\left.\frac{{{\boldsymbol{N}}}^{T}{\boldsymbol{N}}}{{\left[{{\boldsymbol{X}}}_{e}^{T}{{\boldsymbol{N}}}^{T}{\boldsymbol{N}}{{\boldsymbol{X}}}_{e}\right]}^{\raisebox{1ex}{$3$}\!\left/ \!\raisebox{-1ex}{$2$}\right.}}\right|}_{\xi =+1}\right]{{\boldsymbol{X}}}_{{\boldsymbol{e}}}$$

Note that, in the simplest possible case, the tether consists of only one element. The mass matrix is a $\left[9\times 9\right]$ constant matrix (because the tether length and density are constant; please check Appendix B), but ${{\boldsymbol{q}}}_{e}^{l}$, ${{\boldsymbol{q}}}_{e}^{d}$, ${{\boldsymbol{q}}}_{e}^{r}$, and ${{\boldsymbol{q}}}_{e}^{g}$ are $\left[9\times 1\right]$ nonlinear force vectors that are a function of ${\boldsymbol{N}}\left({\boldsymbol{s}}\right)$ and vary with the position (${{\boldsymbol{r}}}_{{\boldsymbol{e}}}$) and the slope (${{\boldsymbol{r}}}_{{\boldsymbol{e}}}^{{\prime}}$). Please see Appendix A for calculating ${{\boldsymbol{N}}}^{{\boldsymbol{T}}}{\boldsymbol{N}}$.

${{\boldsymbol{Q}}}_{e}$ denotes the external force vector, given as follows:

35 $${{\boldsymbol{Q}}}_{e}={{\boldsymbol{N}}}^{T}{{\boldsymbol{F}}}_{ext}$$

According to Figs. 7 and 13, and assuming that in an initial condition all the elements of the tether are under very small tension and the tether is completely straight, then the tensile force vector of the tether exists only in the ${{\boldsymbol{Y}}}_{I}$ direction. But after deformation, the tether’s tension and the robot’s thruster force will have components in both directions (${{\boldsymbol{X}}}_{I}, {{\boldsymbol{Y}}}_{I}$).

**Fig. 14:**
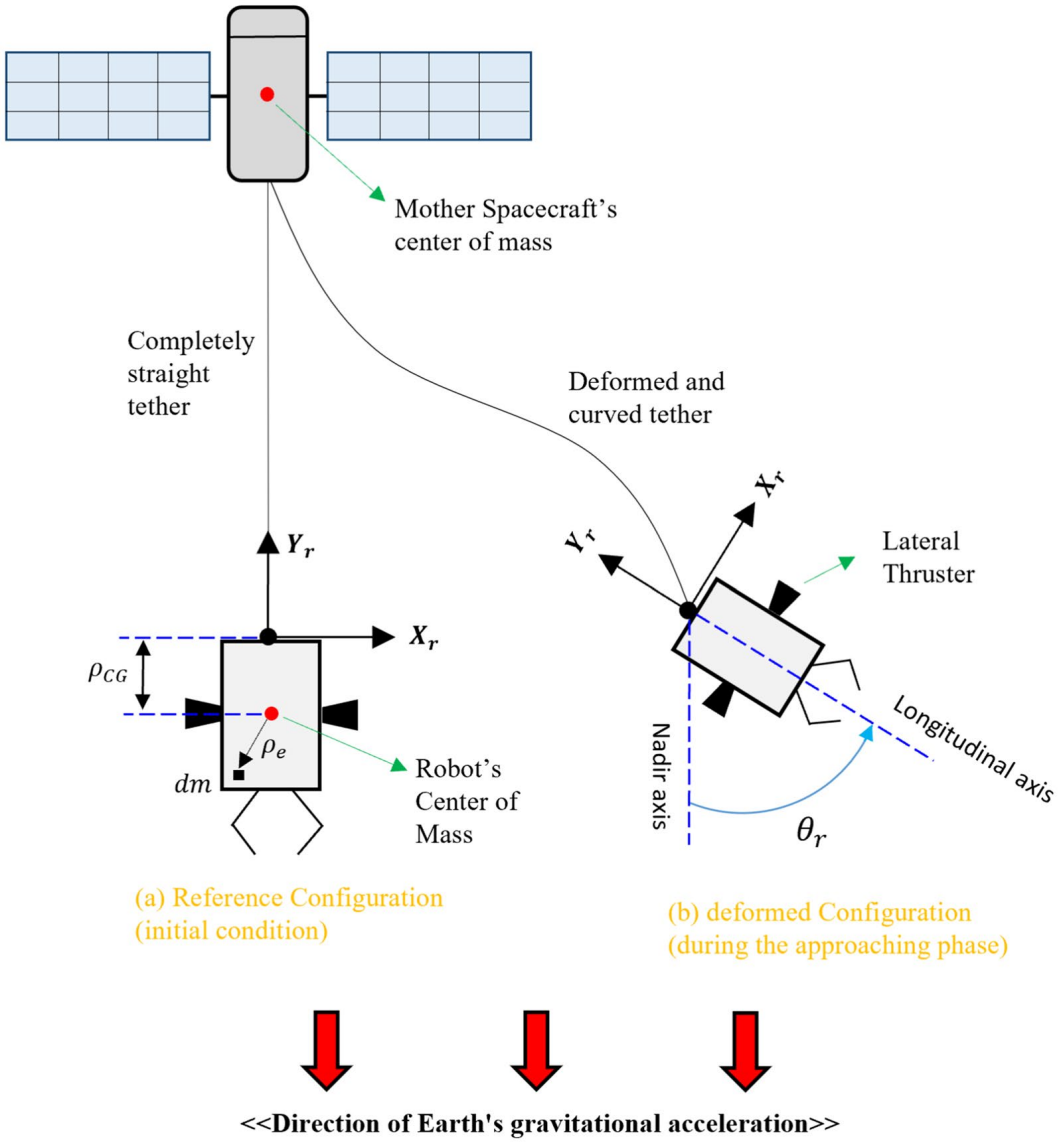
Force vector acting on tether in different conditions.

This initial situation can be expressed in the ANCF method as follows (please see Appendix C):

36 $${{\boldsymbol{F}}}_{ext}={{\boldsymbol{R}}}_{{\boldsymbol{r}}}^{{\boldsymbol{g}}}{\left[\left({f}_{{t}_{x}}+{f}_{{th}_{x}}\right),\left({f}_{{t}_{y}}+{f}_{{th}_{y}}\right)\right]}^{T}$$

where ${f}_{{th}_{x}}$ and ${f}_{{th}_{y}}$ are the reaction thrusters’ force in the ${{\boldsymbol{X}}}_{r}$ and ${{\boldsymbol{Y}}}_{r}$ directions, respectively. ${f}_{{t}_{x}}$ and ${f}_{{t}_{y}}$ are the tether tensile forces in the ${{\boldsymbol{X}}}_{r}$ and ${{\boldsymbol{Y}}}_{r}$ directions, respectively. Note that ${f}_{{t}_{x}}$ is equal to zero in an initial condition (as shown in Fig. 14). It is worth stating that we assumed the tether to be normal to the robot’s surface (${{\boldsymbol{X}}}_{r}$) at the connection point. Thus, in this situation, ${{\boldsymbol{Q}}}_{N}$ can be written for the last element (between node number $N$ and $N+1$ as shown in Fig. 13) as follows:

37 $${\left.{{\boldsymbol{Q}}}_{N}\right|}_{t=0}=\left[\begin{array}{c}{N}_{1}\\ 0\\ {N}_{2}\\ 0\\ {N}_{3}\\ 0\\ {N}_{4}\\ 0\end{array} \begin{array}{c}0\\ {N}_{1}\\ 0\\ {N}_{2}\\ 0\\ {N}_{3}\\ 0\\ {N}_{4}\end{array}\right]{{\boldsymbol{R}}}_{{\boldsymbol{r}}}^{{\boldsymbol{g}}}\left[\begin{array}{c}0\\ {f}_{{t}_{y}}\end{array}\right]=\left[\begin{array}{c}{{\boldsymbol{R}}}_{{\boldsymbol{r}}}^{{\boldsymbol{g}}}\left(1,2\right){f}_{{t}_{y}}\\ 0\\ 0\\ 0\\ 0\\ 0\\ 0\\ 0\end{array}\right]\quad for\quad \xi =-1$$

Note that for $\xi =-1$ the values for ${N}_{2}, {N}_{3}$, and ${N}_{4}$ are equal to zero (based on Eq. (16)), but ${N}_{1}$ is equal to one. According to the above equation, node number $N$ is only affected by the tether’s tensile force.

Similarly, for the last node (N + 1) we have:

38 $${\left.{{\boldsymbol{Q}}}_{N}\right|}_{t=0}=\left[\begin{array}{c}{N}_{1}\\ 0\\ {N}_{2}\\ 0\\ {N}_{3}\\ 0\\ {N}_{4}\\ 0\end{array} \begin{array}{c}0\\ {N}_{1}\\ 0\\ {N}_{2}\\ 0\\ {N}_{3}\\ 0\\ {N}_{4}\end{array}\right]{{\boldsymbol{R}}}_{{\boldsymbol{r}}}^{{\boldsymbol{g}}}\left[\begin{array}{c}{f}_{{th}_{x}}\\ {f}_{{t}_{y}}+{f}_{{th}_{y}}\end{array}\right]=\left[\begin{array}{c}0\\ 0\\ 0\\ 0\\ {{\boldsymbol{R}}}_{{\boldsymbol{r}}}^{{\boldsymbol{g}}}\left(1,2\right)\left({f}_{{t}_{y}}+{f}_{{th}_{y}}\right)\\ {{\boldsymbol{R}}}_{{\boldsymbol{r}}}^{{\boldsymbol{g}}}\left(2,1\right){f}_{{th}_{x}}\\ 0\\ 0\end{array}\right]\quad for\quad \xi =+1$$

Note that for $\xi =+1$ the values for ${N}_{1}, {N}_{2}$, and ${N}_{4}$ are equal to zero, but ${N}_{3}$ is equal to one (based on Eq. (16)). According to the above equation the thruster force is only acting on the last node: node number $N+1$.

For the initial condition considered in Fig. 14 the robot body fixed frame coincides with the inertial global reference frame and $${{\boldsymbol{R}}}_{{\boldsymbol{r}}}^{{\boldsymbol{g}}}=\left[\begin{array}{cc}0& 1\\ -1& 0\end{array}\right]$$, but at other times (after tether deformation) ${{\boldsymbol{R}}}_{{\boldsymbol{r}}}^{{\boldsymbol{g}}}$ can be any arbitrary matrix.

For the middle elements ($e=1, 2, \dots , N-1$) in the initial condition we have:

39 $${\left.{{\boldsymbol{Q}}}_{e}\right|}_{t=0}=\left[\begin{array}{c}{N}_{1}\\ 0\\ {N}_{2}\\ 0\\ {N}_{3}\\ 0\\ {N}_{4}\\ 0\end{array} \begin{array}{c}0\\ {N}_{1}\\ 0\\ {N}_{2}\\ 0\\ {N}_{3}\\ 0\\ {N}_{4}\end{array}\right]{{\boldsymbol{R}}}_{{\boldsymbol{r}}}^{{\boldsymbol{g}}}\left[\begin{array}{c}0\\ {f}_{{t}_{y}}\end{array}\right]=\left[\begin{array}{c}{f}_{{t}_{y}}\\ 0\\ 0\\ 0\\ 0\\ 0\\ 0\\ 0\end{array}\right],\quad for\quad \xi =-1$$

40 $${\left.{{\boldsymbol{Q}}}_{e}\right|}_{t=0}=\left[\begin{array}{c}{N}_{1}\\ 0\\ {N}_{2}\\ 0\\ {N}_{3}\\ 0\\ {N}_{4}\\ 0\end{array} \begin{array}{c}0\\ {N}_{1}\\ 0\\ {N}_{2}\\ 0\\ {N}_{3}\\ 0\\ {N}_{4}\end{array}\right]{{\boldsymbol{R}}}_{{\boldsymbol{r}}}^{{\boldsymbol{g}}}\left[\begin{array}{c}0\\ {f}_{{t}_{y}}\end{array}\right]=\left[\begin{array}{c}0\\ 0\\ 0\\ 0\\ {f}_{{t}_{y}}\\ 0\\ 0\\ 0\end{array}\right],\quad for\quad \xi =+1$$

The symbol ${{\boldsymbol{R}}}_{{\boldsymbol{r}}}^{{\boldsymbol{g}}}\left({\boldsymbol{i}},{\boldsymbol{j}}\right)$ denotes the element located in the i-th row and j-th column of matrix ${{\boldsymbol{R}}}_{{\boldsymbol{r}}}^{{\boldsymbol{g}}}$.

Again, using the Lagrange equation (considering $\theta$ as the generalized coordinate for attitude motion), the dynamic equations of orientational motion of the TSR can be obtained as:

41 $$\frac{d}{dt}{\left(\frac{\partial {K}_{tot}}{\partial {\dot{{\boldsymbol{\theta}}}}_{{\boldsymbol{r}}}}\right)}^{T}-{\left(\frac{\partial {K}_{tot}}{\partial {{\boldsymbol{\theta}}}_{{\boldsymbol{r}}}}\right)}^{T}+{\left(\frac{\partial {U}_{tot}}{\partial {{\boldsymbol{\theta}}}_{{\boldsymbol{r}}}}\right)}^{T}={\left(\frac{\delta W}{\delta {{\boldsymbol{\theta}}}_{{\boldsymbol{r}}}}\right)}^{T}$$

42 $$\frac{d}{dt}{\left(\frac{\partial {K}_{tot}}{\partial {\dot{{\boldsymbol{\theta}}}}_{{\boldsymbol{r}}}}\right)}^{T}={\delta}_{eN}{m}_{r}{\left[{\boldsymbol{N}}{\ddot{{\boldsymbol{X}}}}_{e}\right]}_{@{\boldsymbol{\xi}}=+1}^{T}{{\boldsymbol{R}}}_{{\boldsymbol{r}}}^{{\boldsymbol{g}}}{{\boldsymbol{\rho}}}_{{\boldsymbol{C}}{\boldsymbol{G}}}+{I}_{{r}_{1}}{\dot{{\boldsymbol{\omega}}}}_{{\boldsymbol{r}}}$$

43 $$\left(\frac{\partial {K}_{tot}}{\partial{\boldsymbol{\theta}}}\right)=0$$

44 $$\left(\frac{\partial {U}_{tot}}{\partial{\boldsymbol{\theta}}}\right)=0$$

45 $$\left(\frac{\delta W}{\delta{\boldsymbol{\theta}}}\right)={{\boldsymbol{\tau}}}_{{\boldsymbol{r}}}$$

46 $${I}_{{r}_{1}}{\dot{{\boldsymbol{\omega}}}}_{{\boldsymbol{r}}}+{\delta}_{eN}{m}_{r}{\left[{\boldsymbol{N}}{\ddot{{\boldsymbol{X}}}}_{e}\right]}_{@{\boldsymbol{\xi}}=+1}^{T}{{\boldsymbol{R}}}_{{\boldsymbol{r}}}^{{\boldsymbol{g}}}{{\boldsymbol{\rho}}}_{{\boldsymbol{C}}{\boldsymbol{G}}}={{\boldsymbol{\tau}}}_{{\boldsymbol{r}}}$$

where ${{\boldsymbol{\tau}}}_{{\boldsymbol{r}}}$ denotes the control torque acting on the robot (by attitude control thrusters).

Solving Eqs. (29) and (46) together at the same time led to the simulation results presented in Section "Numerical simulation".

### Relative equations of motion

Many valuable papers have been published in the field of the relative motions of two spacecraft during rendezvous and docking^45,110,111^. Hill equations are useful for explaining the relative translational motion for long-range rendezvous^112^ but are not accurate enough for the proximity phase. For this reason, is they are not used in this paper, and since the target is assumed to be a cooperative, the information about its position and velocity is available to the robot at all times. Therefore, the information related to the position and motion of the tethered robot is directly used in extraction of the control signal.

**Fig. 15:**
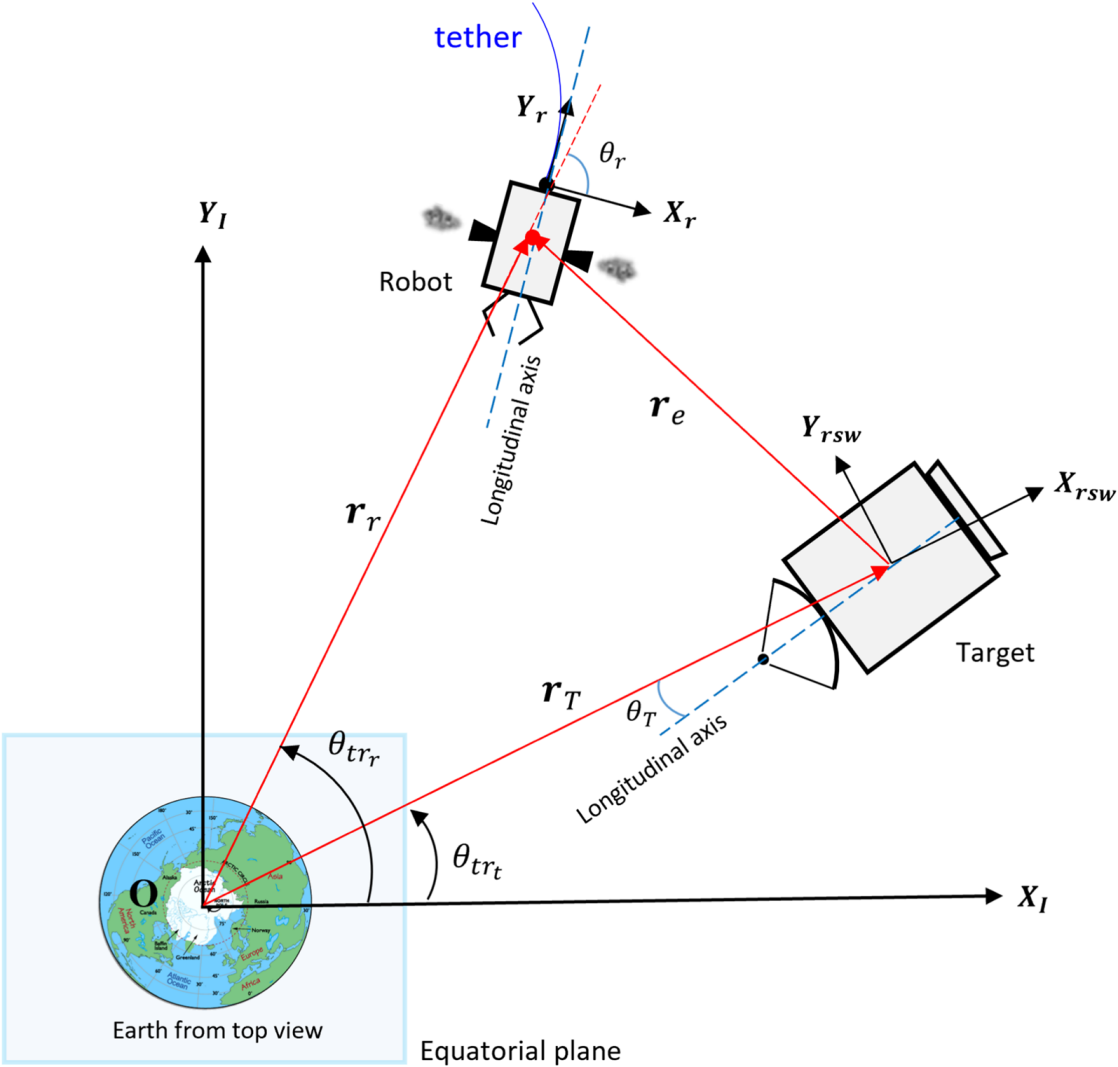
Relative position vector.

Figure 15 shows the relative position vectors between the target and robot spacecraft.

The position (${{\boldsymbol{r}}}_{t}$) and velocity (${{\boldsymbol{v}}}_{t}$) of the target can be expressed in a global reference frame as follows:

47 $${{\boldsymbol{r}}}_{t}=\left|{{\boldsymbol{r}}}_{{\boldsymbol{t}}}\right|{\left[\mathrm{cos}{\theta}_{{tr}_{t}} , \mathrm{sin}{\theta}_{{tr}_{t}}\right]}^{T}$$

48 $${{\boldsymbol{v}}}_{{\boldsymbol{t}}}={\left[{\dot{x}}_{t}, {\dot{y}}_{t}\right]}^{T}=\sqrt{\frac{\mu }{{r}_{t}}}{\left[-\mathrm{sin}{\theta}_{{tr}_{t}} , \mathrm{cos}{\theta}_{{tr}_{t}}\right]}^{T}$$

${\theta}_{{tr}_{t}}$ is the true anomaly of the target spacecraft that can be calculated as ${\omega}_{{0}_{t}}t$, where $t$ is the simulation time. ${\omega}_{{0}_{t}}$ denotes the target spacecraft angular velocity. $\mu =\mathrm{398,601.2} [{\mathrm{k}\mathrm{m}}^{3}{\mathrm{s}}^{-2}]$ is the Earth’s standard gravitational parameter.

49 $$x_{e} = x_{r} - x_{t} ,\quad y_{e} = y_{r} - y_{t}$$

50 $$u_{e} = u_{r} - u_{t} ,\quad v_{e} = v_{r} - v_{t}$$

${x}_{e}$ and ${y}_{e}$ are the relative/error position of the robot and target, and ${\dot{x}}_{e}={u}_{e}$ and ${\dot{y}}_{e}={v}_{e}$ are their relative velocities expressed in a global frame. ${x}_{r}$ and ${y}_{r}$ are the robot positions along x and y axis that can be calculated using Eq. (29) (row number $4N+1$ and $4N+2$, respectively). ${\dot{x}}_{r}={u}_{r}$ and ${\dot{y}}_{r}={v}_{r}$ are the robot velocities along x and y axis.

Using Eq. (46), the relative attitude dynamics equation can be simplified for 2D motion as follows^109^:

51 $$\left[\begin{array}{c}{\dot{\theta }}_{e}\\ {\dot{\omega }}_{e}\end{array}\right]=\left[\begin{array}{cc}0& 1\\ 0& 0\end{array}\right]\left[\begin{array}{c}{\theta}_{e}\\ {\omega}_{e}\end{array}\right]+\left[\begin{array}{c}0\\ {I}_{{r}_{1}}^{-1}\end{array}\right]\left({\tau}_{r}-{\delta}_{eN}{m}_{r}{\left[{\boldsymbol{N}}{\ddot{{\boldsymbol{X}}}}_{e}\right]}_{@{\boldsymbol{\xi}}=+1}^{T}{{\boldsymbol{R}}}_{{\boldsymbol{r}}}^{{\boldsymbol{g}}}{{\boldsymbol{\rho}}}_{{\boldsymbol{C}}{\boldsymbol{G}}}\right)-\left[\begin{array}{c}0\\ {\dot{\omega }}_{t}\end{array}\right]$$

where ${\theta}_{e}={\theta}_{r}-{\theta}_{t}$ and ${\omega}_{e}={\omega}_{r}-{\omega}_{{0}_{t}}$ are the error attitude angle and angular velocity (expressed in robot body-fixed frame).

The rotation matrix from the RSW frame to a global reference frame is given as follows:

52 $${{\boldsymbol{R}}}_{{\boldsymbol{r}}{\boldsymbol{s}}{\boldsymbol{w}}-{\boldsymbol{t}}}^{{\boldsymbol{g}}}=\left[\begin{array}{cc}\mathrm{cos}{\theta}_{{tr}_{t}}& -\mathrm{sin}{\theta}_{{tr}_{t}}\\ \mathrm{sin}{\theta}_{{tr}_{t}}& \mathrm{cos}{\theta}_{{tr}_{t}}\end{array}\right] \to for\, target$$

53 $${{\boldsymbol{R}}}_{{\boldsymbol{r}}{\boldsymbol{s}}{\boldsymbol{w}}-{\boldsymbol{r}}}^{{\boldsymbol{g}}}=\left[\begin{array}{cc}\mathrm{cos}{\theta}_{{tr}_{r}}& -\mathrm{sin}{\theta}_{{tr}_{r}}\\ \mathrm{sin}{\theta}_{{tr}_{r}}& \mathrm{cos}{\theta}_{{tr}_{r}}\end{array}\right] \to for\, robot$$

where ${\theta}_{{tr}_{r}}$ and ${\theta}_{{tr}_{t}}$ are the true anomalies of the robot and target spacecraft, respectively. ${\theta}_{{tr}_{r}}$ and ${\theta}_{{tr}_{t}}$ are very close but not equal in an initial condition.

The rotation matrix from the robot body-fixed frame to the global reference frame can be calculated as ${{\boldsymbol{R}}}_{{\boldsymbol{r}}}^{{\boldsymbol{g}}}={{\boldsymbol{R}}}_{{\boldsymbol{r}}{\boldsymbol{s}}{\boldsymbol{w}}-{\boldsymbol{r}}}^{{\boldsymbol{g}}}{{\boldsymbol{R}}}_{{\boldsymbol{r}}}^{{\boldsymbol{r}}{\boldsymbol{s}}{\boldsymbol{w}}-{\boldsymbol{r}}}$. Similarly, the rotation matrix from a target body-fixed frame to a global reference frame is given as ${{\boldsymbol{R}}}_{{\boldsymbol{t}}}^{{\boldsymbol{g}}}={{\boldsymbol{R}}}_{{\boldsymbol{r}}{\boldsymbol{s}}{\boldsymbol{w}}-{\boldsymbol{t}}}^{{\boldsymbol{g}}}{{\boldsymbol{R}}}_{{\boldsymbol{t}}}^{{\boldsymbol{r}}{\boldsymbol{s}}{\boldsymbol{w}}-{\boldsymbol{t}}}$. Since the thrust forces are applied to the robot’s body, the error position must also be calculated in the tethered robot body frame.

### Controller design

The general format of the controller is written as follows:

54 $${U}_{x}={U}_{{eq}_{x}}+{U}_{{s}_{x}}$$

where ${U}_{{eq}_{x}}$ is known as equivalent control and represents a continuous component (that is responsible for linearizing the equations). ${U}_{{s}_{x}}$ represents a discontinuous component of the controller (that is responsible for providing robustness), which is defined as follows:

55 $${U}_{{s}_{x}}=-{d}_{x}sign\left({S}_{x}\right)$$

where ${d}_{x}$ is positive gain, and ${S}_{x}$ is known as a sliding surface, which is presented in Eq. (56).

56 $${S}_{x}={\dot{x}}_{e}+{D}_{{p}_{x}}{x}_{e}$$

where ${D}_{{p}_{x}}$ is a constant positive gain.

To calculate the term ${U}_{{eq}_{x}}$, it is necessary to calculate the ${\dot{S}}_{x}=0$:

57 $${\dot{S}}_{x}={\ddot{x}}_{e}+{D}_{{p}_{x}}{\dot{x}}_{e}=0 \to \left({\ddot{x}}_{r}-{\ddot{x}}_{t}\right)+{D}_{{p}_{x}}\left({\dot{x}}_{r}-{\dot{x}}_{t}\right)=0$$

The position, velocity, and acceleration of the translational motion of the target satellite in the global frame (for X-axis) are equal to:

58 $${x}_{t}={r}_{t}\mathrm{cos}{\theta}_{t{r}_{t}} \to {\dot{x}}_{t}=-{r}_{t}{\dot{\theta }}_{t{r}_{t}}\mathrm{sin}{\theta}_{t{r}_{t}} \to {\ddot{x}}_{t}=-{r}_{t}{\dot{\theta }}_{{tr}_{t}}^{2}\mathrm{cos}{\theta}_{t{r}_{t}}$$

${\ddot{x}}_{r}$ refers to acceleration of the last node that is the robot center of mass in ${{\boldsymbol{X}}}_{{\boldsymbol{I}}}$ direction (see Eq. 57). Thus, ${\ddot{x}}_{r}$ refers to the (4N + 1)th row of Eq. (29) that can be written as:

59 $$\sum_{n=1}^{N}\left({a}_{i,j}{\ddot{x}}_{n}+{a}_{i,jj}{\ddot{x}}_{n}^{\prime}\right)+{a}_{i,jjj}{\ddot{x}}_{N+1}^{\prime}+{a}_{c,c}{\ddot{x}}_{N+1}=-{\left({{\boldsymbol{q}}}_{e}^{r}+{{\boldsymbol{q}}}_{e}^{l}+{{\boldsymbol{q}}}_{e}^{d}+{{\boldsymbol{q}}}_{e}^{g}\right)}_{c}+{\left({{\boldsymbol{Q}}}_{e}\right)}_{cx}$$

where ${a}_{i,j}$, ${a}_{i,jj}$, ${a}_{i,jjj}$, and ${a}_{c,c}$ are elements of the mass matrix, and $i=c=4N+1$, $j=\left(4n-3\right)$, $jj=\left(4n-1\right)$, and $jjj=\left(4\left(N+1\right)-1\right)$ denote the rows and columns of the elements, and ${\left({{\boldsymbol{Q}}}_{e}\right)}_{cx}$ is equal to: ${{\boldsymbol{R}}}_{{\boldsymbol{r}}}^{{\boldsymbol{g}}}\left(1,1\right)\left({f}_{{t}_{x}}+{f}_{{th}_{x}}\right)+{{\boldsymbol{R}}}_{{\boldsymbol{r}}}^{{\boldsymbol{g}}}\left(1,2\right)\left({f}_{{t}_{y}}+{f}_{{th}_{y}}\right)$.

According to Eq. (59), ${\ddot{x}}_{N+1}$ will be as follows:

60 $${\ddot{x}}_{N+1}=\frac{1}{{a}_{c,c}}\left(-\sum_{n=1}^{N}\left({a}_{i,j}{\ddot{x}}_{n}+{a}_{i,jj}{\ddot{x}}_{n}^{\prime}\right)-{a}_{i,jjj}{\ddot{x}}_{N+1}^{\prime}-{\left({{\boldsymbol{q}}}_{e}^{r}+{{\boldsymbol{q}}}_{e}^{l}+{{\boldsymbol{q}}}_{e}^{d}+{{\boldsymbol{q}}}_{e}^{g}\right)}_{c}+{\left({{\boldsymbol{Q}}}_{e}\right)}_{cx}\right)$$

For simplicity, we use variable transformation ${\left({{\boldsymbol{q}}}_{e}^{r}+{{\boldsymbol{q}}}_{e}^{l}+{{\boldsymbol{q}}}_{e}^{d}+{{\boldsymbol{q}}}_{e}^{g}\right)}_{c}={qq}_{x}$. Also, we know in the *x*-axis of the body frame the term $\left({f}_{{t}_{y}}+{f}_{{th}_{y}}\right)$ is zero.

${\ddot{x}}_{N+1}$ can be expressed in the body frame as follows:

61 $${\ddot{x}}_{N+1}={R}_{r}^{{g}^{-1}}\left(\frac{1}{{a}_{c,c}}\left(-\sum_{n=1}^{N}\left({a}_{i,j}{\ddot{x}}_{n}+{a}_{i,jj}{\ddot{x}}_{n}^{\prime}\right)-{a}_{i,jjj}{\ddot{x}}_{N+1}^{\prime}-{qq}_{x}\right)\right)+\frac{1}{{a}_{c,c}}\left({f}_{t{h}_{x}}+{f}_{{t}_{x}}\right)$$

Note that, in the proximity phase, accuracy is increasingly important, and a catastrophic disaster can occur in the presence of delay or error. This is why the tether force is not considered a control force in this phase, so the tether’s tension force is treated as a disturbance force, although after capturing the target, it can be counted as the control force.

Substituting Eq. (61) in (57) gives:

62 $$\begin{aligned}{\dot{S}}_{x}&=0 \to {R}_{r}^{{g}^{-1}}\left(\frac{1}{{a}_{c,c}}\left(-\sum_{n=1}^{N}\left({a}_{i,j}{\ddot{x}}_{n}+{a}_{i,jj}{\ddot{x}}_{n}^{\prime}\right)-{a}_{i,jjj}{\ddot{x}}_{N+1}^{\prime}-{qq}_{x}\right)\right)\\ &\quad+\frac{1}{{a}_{c,c}}{f}_{t{h}_{x}}+{r}_{t}{\dot{\theta }}_{{tr}_{t}}^{2}\mathrm{cos}{\theta}_{t{r}_{t}}+{D}_{{p}_{x}}\left({\dot{x}}_{r}-{\dot{x}}_{t}\right)=0\end{aligned}$$

Now, ${U}_{{eq}_{x}}$ can be calculated so that the relation ${\dot{S}}_{x}=0$ is realized, regardless of disturbances and uncertainties.

63 $$\begin{aligned}{U}_{{eq}_{x}}&={a}_{c,c}\left(-{D}_{{p}_{x}}\left({\dot{x}}_{r}-{\dot{x}}_{t}\right)-{r}_{t}{\dot{\theta }}_{{tr}_{t}}^{2}\mathrm{cos}{\theta}_{t{r}_{t}}\right)\\ &\quad+{R}_{r}^{{g}^{-1}}\left(\left(\sum_{n=1}^{N}\left({a}_{i,j}{\ddot{x}}_{n}+{a}_{i,jj}{\ddot{x}}_{n}^{\prime}\right)+{a}_{i,jjj}{\ddot{x}}_{N+1}^{\prime}+{qq}_{x}\right)\right)\end{aligned}$$

Now it is time to calculate the ${d}_{x}$ in order to ensure the robustness of the controller against disturbance forces. For this purpose, Lyapunov stability theory is used. Lyapunov candidate function is defined as follows:

64 $${V}_{x}=\frac{1}{2}{S}_{x}^{2}$$

where ${V}_{x}$ is the Lyapunov function that is a positive definite function.

Now we only need to prove that the derivative of the Lyapunov function is negative definite.

65 $${\dot{V}}_{x}={S}_{x}{\dot{S}}_{x}\le -{\mu}_{x}\left|{S}_{x}\right|$$

where ${\mu}_{x}$ is a positive constant.

Using Eqs. (54) and (61) for ${\dot{S}}_{x}$, Eq. (65) will turn to:

66 $${S}_{x}\frac{1}{{a}_{c,c}}\left({f}_{{t}_{x}}-{d}_{x}sign\left({S}_{x}\right)\right)\le -{\mu}_{x}\left|{S}_{x}\right|$$

67 $${S}_{x}{f}_{{t}_{x}}-\left|{S}_{x}\right|{d}_{x}\le -{a}_{c,c}{\mu}_{x}\left|{S}_{x}\right|$$

${d}_{x}$ should compensate for the maximum disturbance force. Assuming that ${f}_{{t}_{x-max}}$ is the upper bound of tether’s disturbance force, then we have $\left|{f}_{{t}_{x}}\right|\le {f}_{{t}_{x-max}}$. Also, it is clear that ${S}_{x}{f}_{{t}_{x}}\le \left|{S}_{x}{f}_{{t}_{x}}\right|$ and ${S}_{x}{f}_{{t}_{x}}\le \left|{S}_{x}\right|\left|{f}_{{t}_{x}}\right|$. Thus, it can be accepted as a fact that ${S}_{x}{f}_{{t}_{x}}\le \left|{S}_{x}\right|{f}_{{t}_{x-max}}$.

68 $$\left|{S}_{x}\right|{f}_{{t}_{x-max}}-\left|{S}_{x}\right|{d}_{x}\le -{\mu}_{x}\left|{S}_{x}\right|$$

69 $${d}_{x}\ge \left({a}_{c,c}{\mu}_{x}+{f}_{{t}_{x-max}}\right)$$

Similarly, for the y-axis we have:

70 $${U}_{y}={U}_{{eq}_{y}}+{U}_{{s}_{y}}$$

71 $${U}_{{s}_{y}}=-{d}_{y}sign\left({S}_{y}\right)$$

72 $${S}_{y}={\dot{y}}_{e}+{D}_{{p}_{y}}{y}_{e}$$

where ${U}_{{eq}_{y}}$ and ${U}_{{s}_{y}}$ are the continuous and discontinuous terms of the controller. ${D}_{{p}_{y}}$ is a positive constant gain.

73 $${\dot{S}}_{y}={\ddot{y}}_{e}+{D}_{{p}_{y}}{\dot{y}}_{e}=0 \to \left({\ddot{y}}_{r}-{\ddot{y}}_{t}\right)+{D}_{{p}_{y}}\left({\dot{y}}_{r}-{\dot{y}}_{t}\right)=0$$

${\ddot{y}}_{r}$ is the acceleration of robot body in the y-axis. ${\ddot{y}}_{N}$, which is the acceleration of the last node on the tether in the y-axis, refers to the (4N + 2)th row of Eq. (29).

As expected from an orbiter in circular orbit (shown in Fig. 15), ${\dot{y}}_{t}$ and ${\ddot{y}}_{t}$ can be calculated as:

74 $${y}_{t}={r}_{t}\mathrm{sin}{\theta}_{t{r}_{t}} \to {\dot{y}}_{t}={r}_{t}{\dot{\theta }}_{t{r}_{t}}\mathrm{cos}{\theta}_{t{r}_{t}} \to {\ddot{y}}_{t}=-{r}_{t}{\dot{\theta }}_{{tr}_{t}}^{2}\mathrm{sin}{\theta}_{t{r}_{t}}$$

${\ddot{y}}_{N+1}$ can be calculated using Eqs. (29), (73), and (74) as follows:

75 $${\ddot{y}}_{N+1}=\frac{1}{{a}_{c,c}}\left(-\sum_{n=1}^{N}\left({a}_{i,j}{\ddot{y}}_{n}+{a}_{i,jj}{\ddot{y}}_{n}^{\prime}\right)-{a}_{i,jjj}{\ddot{y}}_{N+1}^{\prime}-{\left({{\boldsymbol{q}}}_{e}^{r}+{{\boldsymbol{q}}}_{e}^{l}+{{\boldsymbol{q}}}_{e}^{d}+{{\boldsymbol{q}}}_{e}^{g}\right)}_{c}+{\left({{\boldsymbol{Q}}}_{e}\right)}_{cy}\right)$$

where here there are different definitions for counters: $i=c=4n+2$, $j=\left(4n-2\right)$, $jj=4n$, and $jjj=4\left(N+1\right)$. Also, ${\left({{\boldsymbol{Q}}}_{e}\right)}_{cy}={{\boldsymbol{R}}}_{{\boldsymbol{r}}}^{{\boldsymbol{g}}}\left(2,1\right)\left({f}_{{t}_{x}}+{f}_{{th}_{x}}\right)+{{\boldsymbol{R}}}_{{\boldsymbol{r}}}^{{\boldsymbol{g}}}\left(2,2\right)\left({f}_{{t}_{y}}+{f}_{{th}_{y}}\right)$.

Here, ${f}_{{th}_{y}}$ is the unknown that should be found in robot body frame. Using the variable transformation ${\left({{\boldsymbol{q}}}_{e}^{r}+{{\boldsymbol{q}}}_{e}^{l}+{{\boldsymbol{q}}}_{e}^{d}+{{\boldsymbol{q}}}_{e}^{g}\right)}_{c}={qq}_{y}$, the ${U}_{{eq}_{y}}$ that makes the relation ${\dot{S}}_{y}=0$ come true is:

76 $${U}_{{eq}_{y}}={a}_{c,c}\left(-{D}_{{p}_{y}}\left({\dot{y}}_{r}-{\dot{y}}_{t}\right)-{r}_{t}{\dot{\theta }}_{{tr}_{t}}^{2}\mathrm{sin}{\theta}_{t{r}_{t}}\right)+{R}_{r}^{{g}^{-1}}\left(\left(\sum_{n=1}^{N}\left({a}_{i,j}{\ddot{y}}_{n}+{a}_{ij}{\ddot{y}}_{i,jj}^{\prime}\right)+{a}_{i,jjj}{\ddot{y}}_{N+1}^{\prime}+{qq}_{y}\right)\right)$$

To find the proper ${d}_{y}$ we need to stick to the Lyapunov stability theory. ${V}_{y}$, which is considered a Lyapunov candidate function, is presented as:

77 $${V}_{y}=\frac{1}{2}{S}_{y}^{2}$$

${V}_{y}$ is a positive definite function, and we have to find the ${d}_{y}$ in such a way that ${\dot{V}}_{y}$ is a negative definite function, even in the presence of disturbances:

78 $${\dot{V}}_{y}={S}_{y}{\dot{S}}_{y}\le -{\mu}_{y}\left|{S}_{y}\right|$$

Finally, the ${d}_{y}$ that assures system stability is:

79 $${d}_{y}\ge \left({a}_{c,c}{\mu}_{y}+{f}_{{t}_{y-max}}\right)$$

Without loss of generality, based on the described policy, a controller can also be designed for the rotational motion of the robot.

Again, the general format of the control signal will be as follows:

80 $${U}_{\theta }={U}_{{eq}_{\theta }}+{U}_{{s}_{\theta }}$$

81 $${U}_{{s}_{\theta }}=-{d}_{\theta }sign\left({S}_{\theta }\right)$$

The allowed range of ${d}_{\theta }$ will later be used to prove controller stability through Lyapunov theory.

First, it is necessary to define the sliding surface:

82 $${S}_{\theta }={\dot{\theta }}_{e}+{D}_{{p}_{\theta }}{\theta}_{e}$$

When the sliding level becomes zero (at the phase plane origin), the control objectives will also be met.

The time derivative of the sliding surface is:

83 $${\dot{S}}_{\theta }={\ddot{\theta }}_{e}+{D}_{{p}_{\theta }}{\dot{\theta }}_{e}$$

84 $${\dot{S}}_{\theta }=\left({\ddot{\theta }}_{r}-{\ddot{\theta }}_{t}\right)+{D}_{{p}_{\theta }}\left({\omega}_{r}-{\omega}_{t}\right)$$

where ${\omega}_{t}$ denotes target angular velocity measured by its own gyroscope.

Note that the error/relative angular velocity $\left({\omega}_{r}-{\omega}_{t}\right)$ and relative attitude (${\theta}_{e}={\theta}_{r}-{\theta}_{t}$) can be measured by relative navigation methods (RADAR, LIDAR, vision-based navigation) directly.

The target is assumed to be cooperative and stable. Thus, its angular velocity and acceleration are equal to zero $\left({\ddot{\theta }}_{t}=0 \mathrm{a}\mathrm{n}\mathrm{d} {\omega}_{t}=0\right)$.

Substituting ${\ddot{\theta }}_{r}={\dot{\omega }}_{r}$ from Eqs. (46) and (84) gives:

85 $$\left({I}_{{r}_{1}}^{-1}\left({{\boldsymbol{\tau}}}_{{\boldsymbol{r}}}-{m}_{r}{\left[{\boldsymbol{N}}{\ddot{{\boldsymbol{X}}}}_{N}\right]}_{@{\boldsymbol{\xi}}=+1}^{T}{{\boldsymbol{R}}}_{{\boldsymbol{r}}}^{{\boldsymbol{g}}}{{\boldsymbol{\rho}}}_{{\boldsymbol{C}}{\boldsymbol{G}}}\right)\right)+{D}_{{p}_{\theta }}\left({\omega}_{r}\right)=0$$

The equivalent control signal that can push ${\dot{S}}_{\theta }$ to zero is:

86 $${{\boldsymbol{\tau}}}_{{{\boldsymbol{r}}}_{{\boldsymbol{e}}{\boldsymbol{q}}}}={\delta}_{eN}{m}_{r}{\left[{\boldsymbol{N}}{\ddot{{\boldsymbol{X}}}}_{e}\right]}_{@{\boldsymbol{\xi}}=+1}^{T}{{\boldsymbol{R}}}_{{\boldsymbol{r}}}^{{\boldsymbol{g}}}{{\boldsymbol{\rho}}}_{{\boldsymbol{C}}{\boldsymbol{G}}}-{I}_{{r}_{1}}{D}_{{p}_{\theta }}\left({\omega}_{r}\right)$$

To calculate a suitable ${d}_{\theta }$ for making the controller robust to disturbance torques, the candidate Lyapunov function is defined as follows:

87 $${V}_{\theta }=\frac{1}{2}{S}_{\theta }^{2}$$

${V}_{\theta }$ is a positive definite function. To prove the controller stability, it is enough to find ${d}_{\theta }$ in such a way that ${\dot{V}}_{\theta }$ is negative definite:

88 $${\dot{V}}_{\theta }={S}_{\theta }{\dot{S}}_{\theta }\le -{\mu}_{\theta }\left|{S}_{\theta }\right|$$

Using Eqs. (80), (84), and (88) we will have:

89 $${\dot{V}}_{\theta }={I}_{{r}_{1}}^{-1}\left|{S}_{\theta }\right|\left({\tau}_{d-max}-{d}_{\theta }\right)\le -{\mu}_{\theta }\left|{S}_{\theta }\right|$$

where ${\tau}_{d-max}$ is the upper bound of the total (tether or any other) disturbance torque.

90 $${d}_{\theta }\ge \left({I}_{{r}_{1}}{\mu}_{\theta }+{\tau}_{d-max}\right)$$

As the value of ${d}_{\theta }$ increases, the stability of the controller will be guaranteed in a wider range, but the control effort will also increase.

## Numerical simulation

In this part, in order to validate the extracted equations and the correctness of the coding, first, the simulation of two scenarios that were previously performed by other researchers is performed, and the results are compared (Section "Verification of the equations and coding"). Next, by defining three simple, conventional, and well-known scenarios, the simulation results were compared with the spring–mass system.

Then, the information related to the main mission is presented, and the simulation results are analyzed (Section "Main mission scenario").

### Verification of the equations and coding

**Fig. 16:**
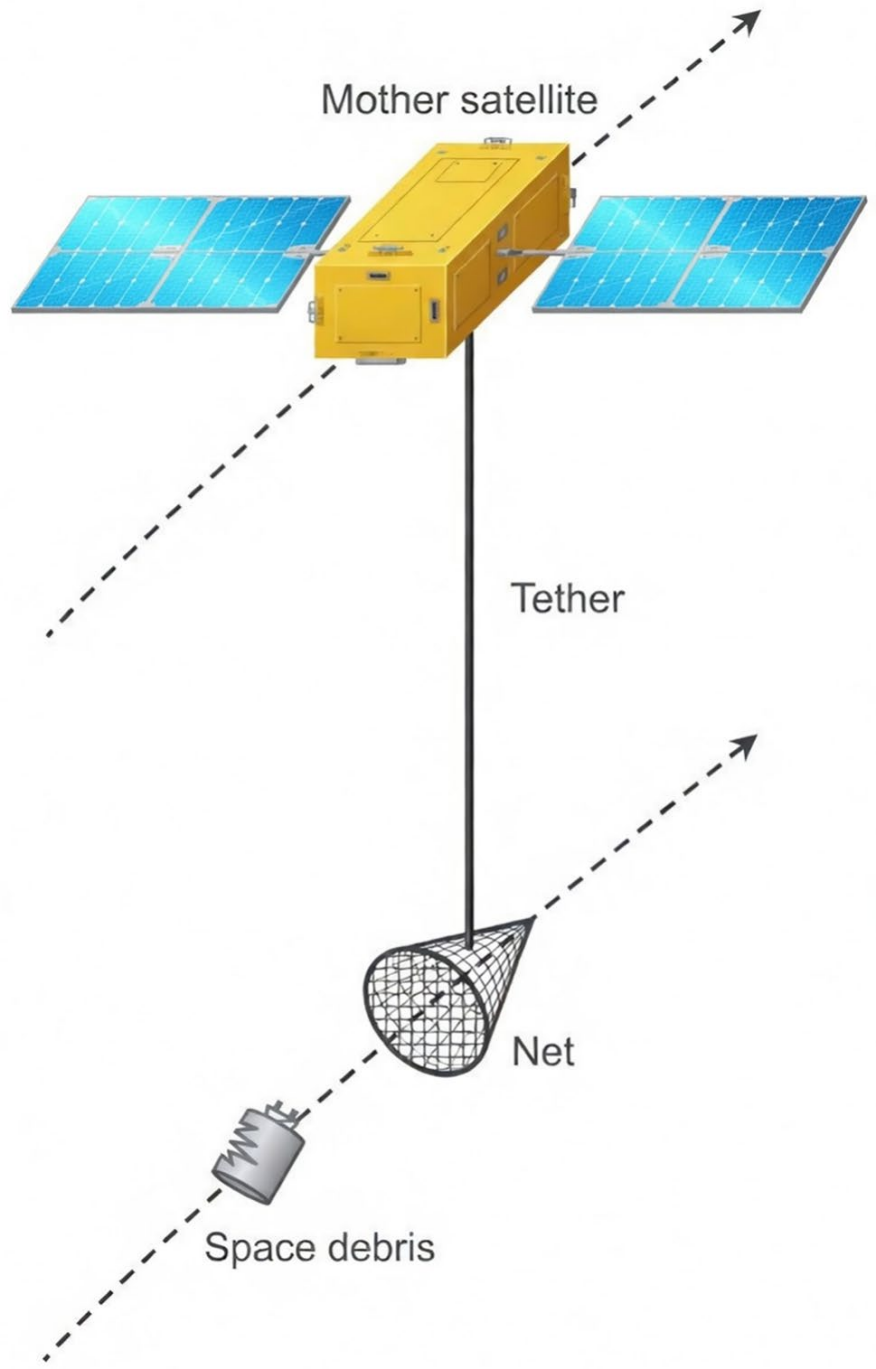
Tethered space system presented in^89^.

**Part 1:** To verify the equations of motion derived for the tether using the ANCF method, our focus lies in conducting a comparative analysis between our simulation outcomes and the findings depicted in Figs. 5 and 8 of references^89^ and^84^, respectively. Furthermore, it should be noted that the input data utilized for this simulation are identical to the data employed in the aforementioned references. Note that, in this part, both the mother and robot spacecraft are assumed to be point masses, and there is no control or disturbance torque or force acting on the system. Figure 16 shows the schematic of the TSS under discussion.

To begin, the scenario presented in^85^ will be discussed. The system parameters of the TSS are: $L=\mathrm{10,000} [\mathrm{k}\mathrm{m}],$ $EA={10}^{6} [\mathrm{N}]$, $\rho =1 \left[\mathrm{k}\mathrm{g}/\mathrm{k}\mathrm{m}\right]$, ${m}_{m}={m}_{n}=500 [\mathrm{k}\mathrm{g}]$, $\alpha =0 \left[\mathrm{s}\right],$ and $N=40$. The tether’s mass distribution is homogeneous and uniform; thus, the center of the mass of the system (${r}_{CG}$) is located at 15,000 km from the center of Earth in the initial condition. The tether is assumed to be totally straight, and the initial positions of all nodes are aligned along the $+X$ axis. For all nodes we considered ${x}_{e}^{{\prime}}=-1$ and ${y}_{e}^{{\prime}}=0$. The initial velocity of each node is in the $+Y$ direction, which can be calculated as:

91 $${\left.V(s)\right|}_{@t=0}=\Vert {\boldsymbol{r}}({\boldsymbol{s}})\Vert \sqrt{\frac{\mu }{{\Vert {{\boldsymbol{r}}}_{{\boldsymbol{C}}{\boldsymbol{G}}}\Vert }^{3}}}$$

**Fig. 17:**
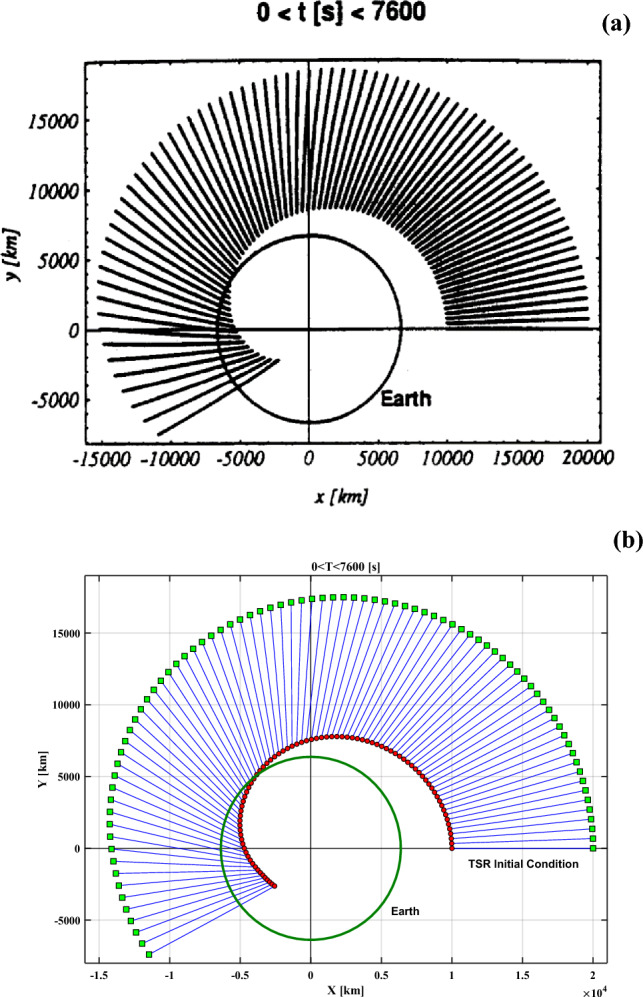
Motion of a two-satellite TSS equipped with a long tether starting from the radial relative equilibrium position: (**a**) is presented in^84^, (**b**) is from the present study.

Figure 17 shows result similar to those presented in Figs. 5 and 8 in^84,89^, respectively.

Note that ${m}_{m}$ and ${m}_{n}$ represent point-masses attached to the two ends of the tether.

**Fig. 18:**
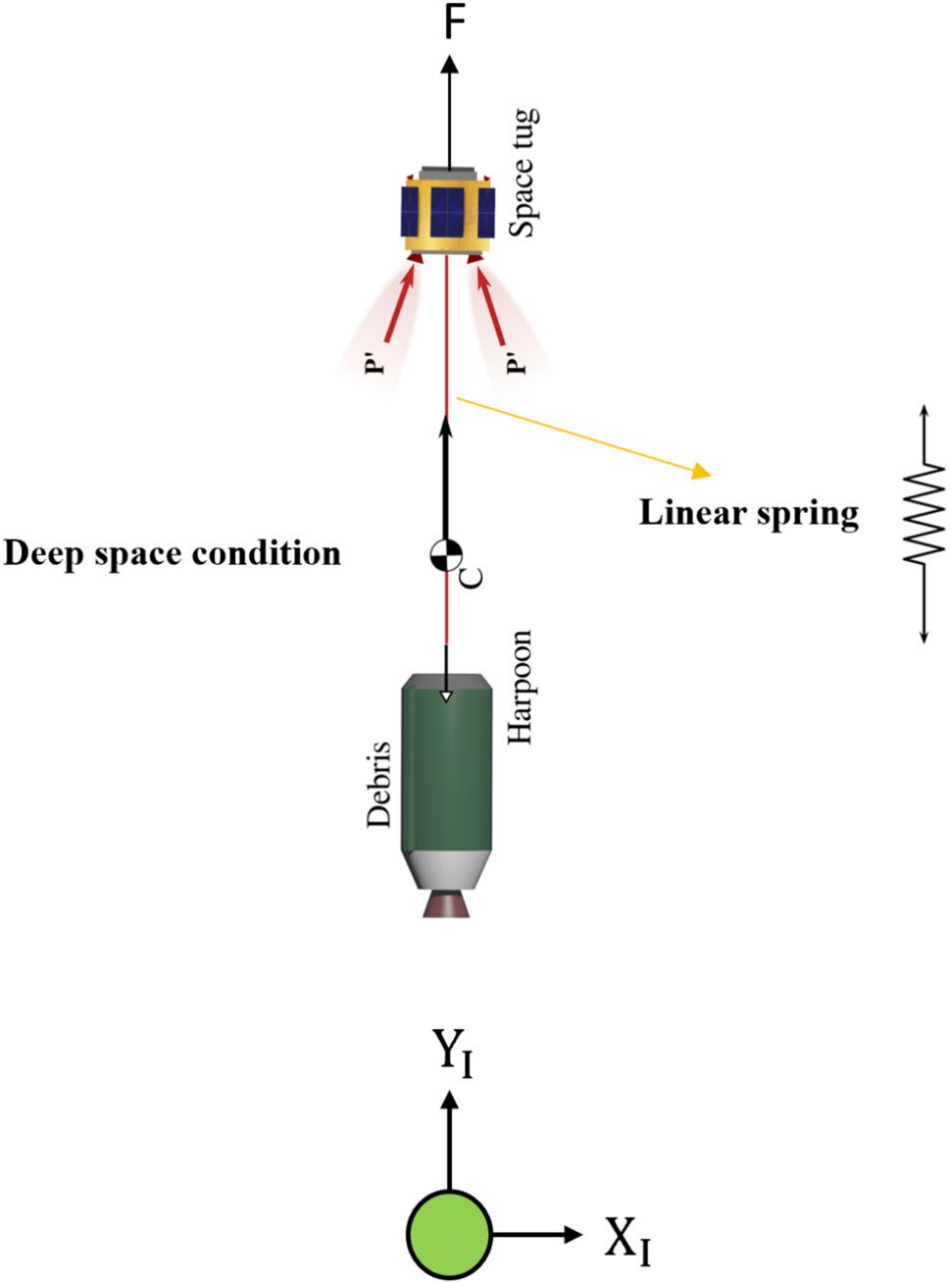
Space tug system presented in^104^.

**Part 2:** As a second check, we verified (i) the variations of the tether length and (ii) its corresponding tension force by comparing our simulation results with results reported in Figs. 7 and 9 of references^89,113^, respectively. Jasper et al.^113^ modelled the space tug system (shown in Fig. 18) as a simple mass–spring system with one degree of freedom (in the ${{\boldsymbol{Y}}}_{{\boldsymbol{I}}}$ direction) under no gravity.

Similar to reference^89^, we considered the simulation input data as $L=1 [\mathrm{k}\mathrm{m}]$, $EA=24\times {10}^{3} [\mathrm{N}]$, $\rho =0.0104 \left[\mathrm{k}\mathrm{g}/\mathrm{m}\right]$, ${m}_{m}=2717 [\mathrm{k}\mathrm{g}]$, ${m}_{n}=1500 [\mathrm{k}\mathrm{g}]$, $\alpha =0 \left[\mathrm{s}\right],$ and $N=1$. We applied a force of $F=5 [\mathrm{k}\mathrm{N}]$ in the $+Y$ direction for 101 s to the mother spacecraft. The findings in the absence of gravity are described below.

**Fig. 19:**
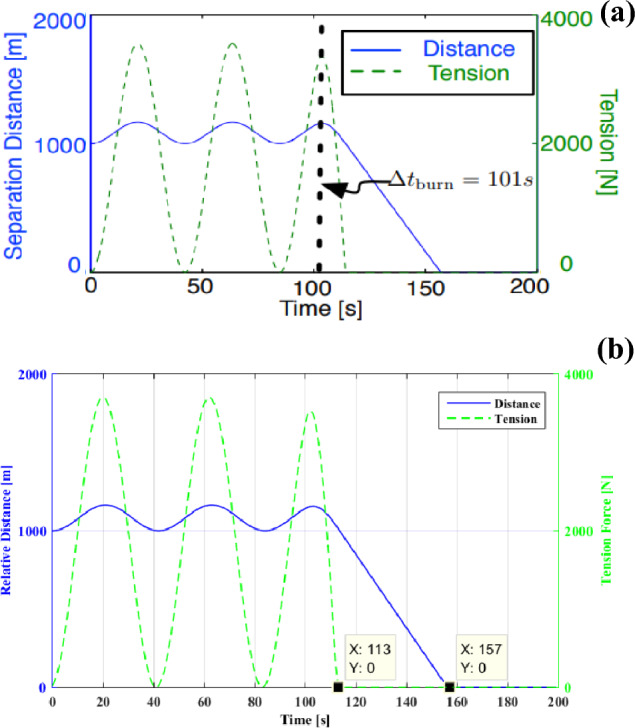
Variation of the tether’s length and tension (**a**) presented in Jasper et al.^113^ and as the (**b**) output of our simulation.

According to the Fig. 19, after 113 s, when the length of the tether became less than its initial standard length ($1000 \mathrm{m}$), its tensile force also became zero. Also, the relative distance between the two endpoint masses has reached zero after 157 s. The results of the simulations are in complete agreement with what is mentioned in^89,113^.

**Fig. 20:**
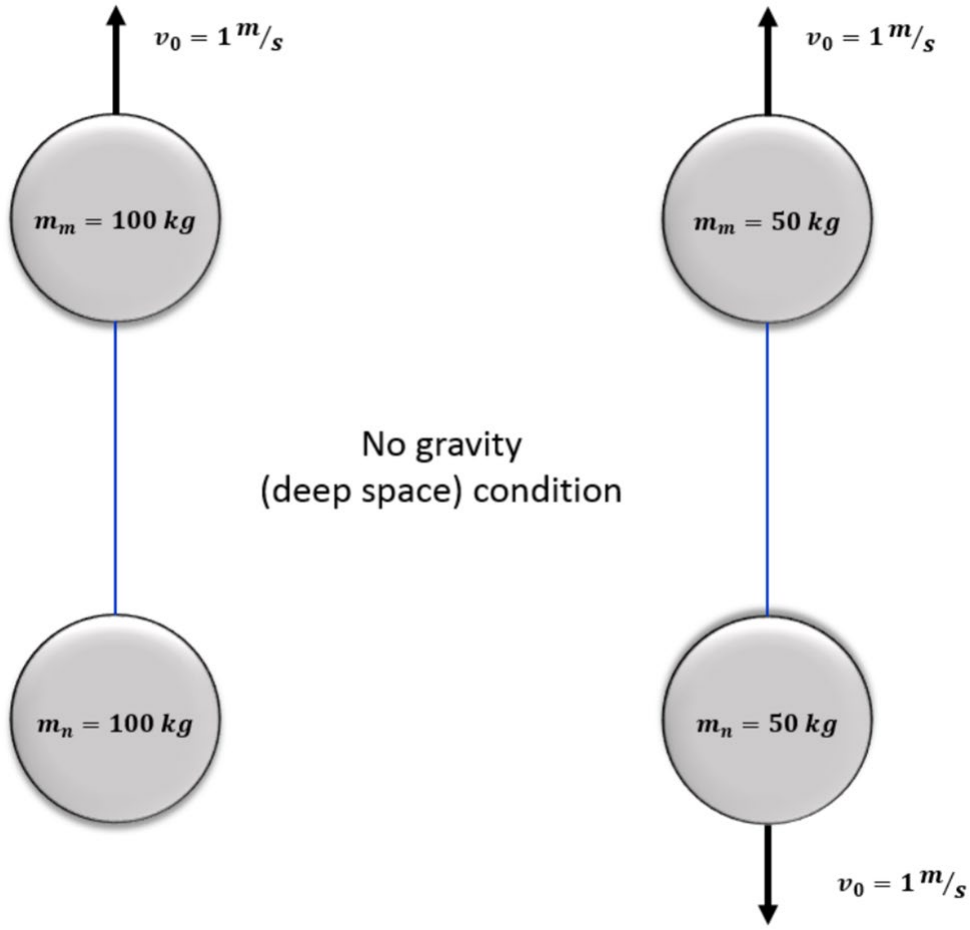
Variation of tether length and tension during simulation.

**Part 3:** In this section, we wish to treat the tether as a linear spring. In addition, in this section, it is assumed that the tether withstands compressive stress ($\varepsilon <0$ can also exist). At first, as shown on the left side of Fig. 20, the tether is between two 100 kg point masses and is straight (but not stretched). In the absence of external force, only the upper mass is thrown upward with an initial velocity of ${v}_{0}=1 \left[\mathrm{m}/\mathrm{s}\right]$. But on the right side of Fig. 20, two 50 kg masses are attached to the end of the tether, which are thrown in two opposite directions with an initial speed of ${v}_{0}=1 \left[\mathrm{m}/\mathrm{s}\right]$. In both cases, gravity is ignored, and the simulation inputs are: $L=1 \left[\mathrm{k}\mathrm{m}\right], E=270 \left[\mathrm{G}\mathrm{P}\mathrm{a}\right], A=8\times {10}^{-6} \left[{\mathrm{m}}^{2}\right], \rho =0.001 \left[\mathrm{k}\mathrm{g}/\mathrm{m}\right], \mathrm{a}\mathrm{n}\mathrm{d} N=1$.

**Fig. 21:**
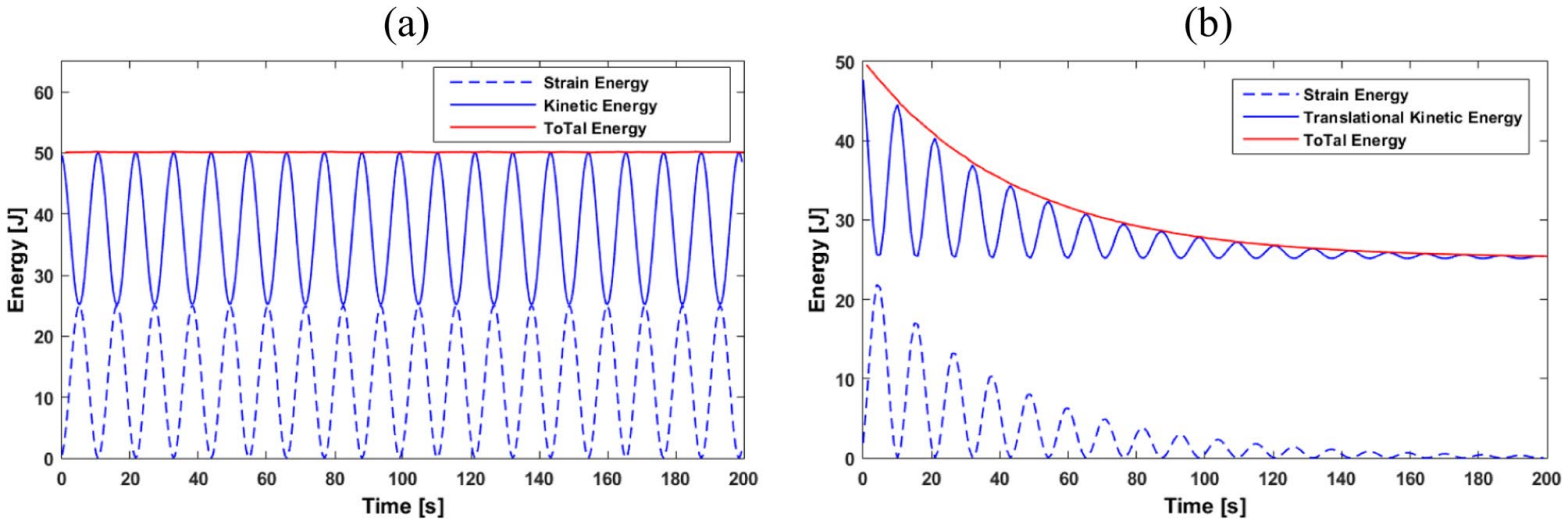
Energy time history (**a**) in conservative conditions and (**b**) under damping (plotted for the left-hand side of the Fig. 20).

**Fig. 22:**
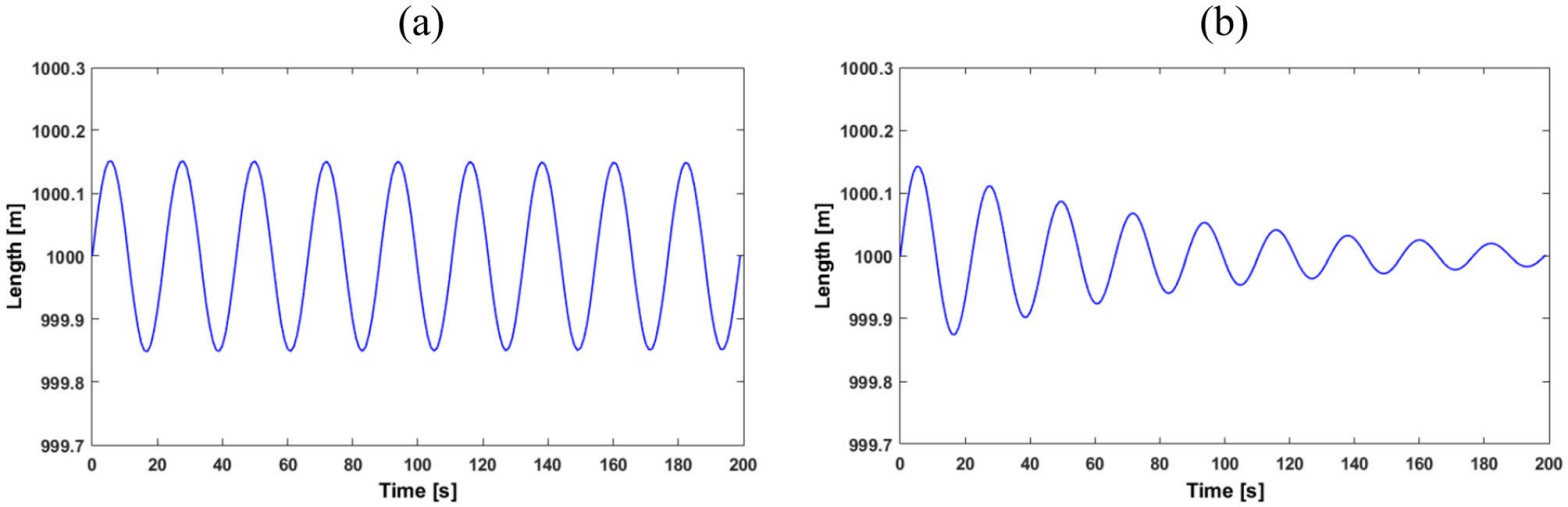
Tether length variation (**a**) under conservative conditions and (**b**) under damping (plotted for the left-hand side of the Fig. 20).

Here, analysis of the simulation results is conducted for the system depicted in Fig. 20 on the left. As depicted in Fig. 21a, in the absence of damping and under conservative conditions, the system solely undergoes conversion of kinetic energy and elastic potential energy with a constant domain. Consequently, the internal energy of the system (solid red line in Fig. 21a) remains constant. In the presence of damping (Fig. 21b), it can be observed that the elastic potential energy eventually diminishes to zero, and the whole system moves like a rigid body while the tether reaches its initial and standard length. Since then, the internal energy of the whole system remains constant (equal to kinetic energy). The corresponding changes in tether length for these two scenarios are illustrated in Figs. 22a and b, respectively.

Figures 21 and 22 show the simulation results of the system depicted on the left-hand side of Fig. 20.

**Fig. 23:**
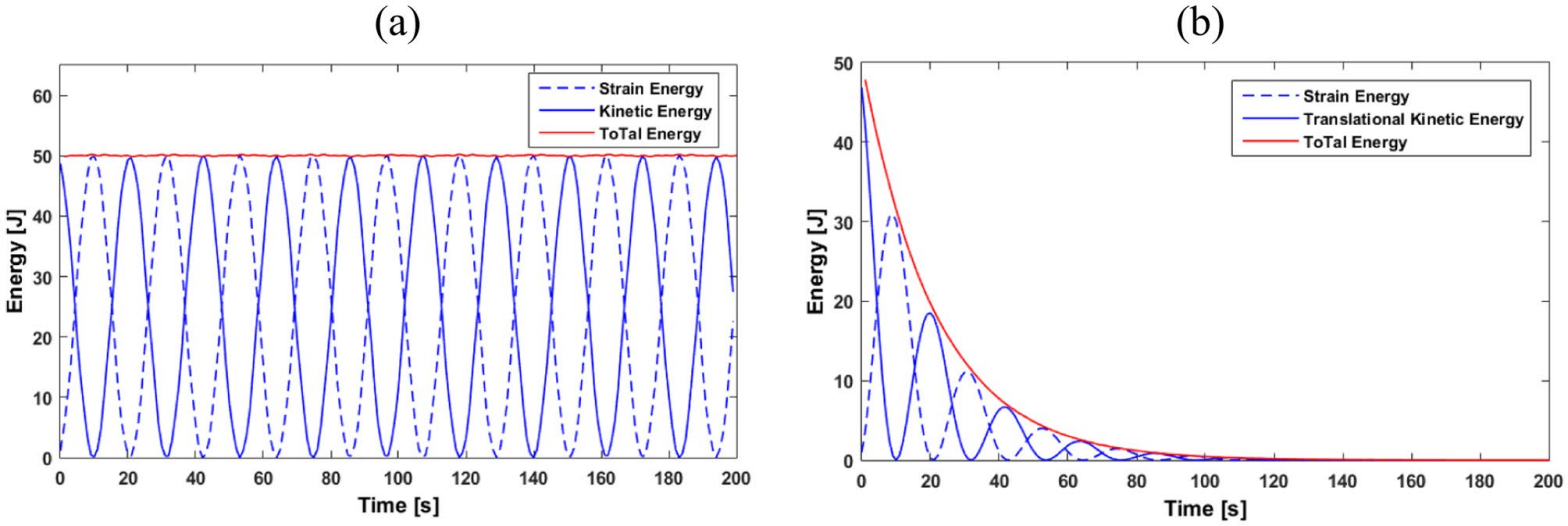
Energy time history (**a**) in conservative conditions and (**b**) under damping (plotted for the right-hand side of the Fig. 20).

**Fig. 24:**
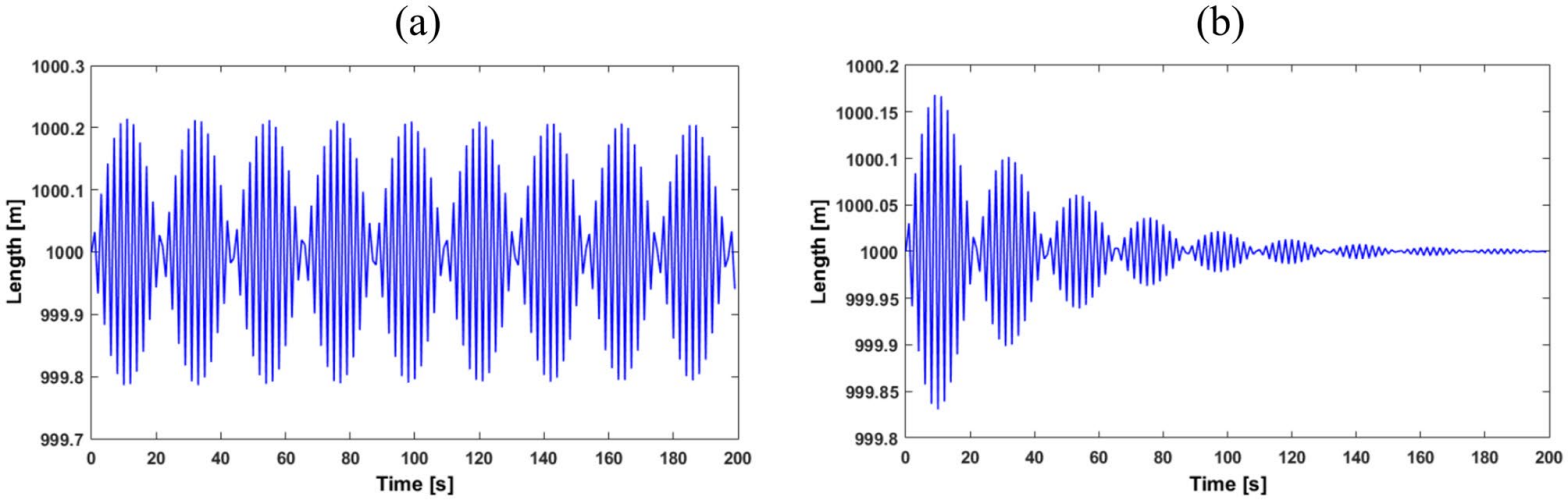
Tether length variation (**a**) under conservative conditions and (**b**) under damping (plotted for the right-hand side of the Fig. 20).

Figures 23 and 24 show the simulation results related to the system depicted on the right-hand side of Fig. 20. According to Fig. 23a, in the absence of external forces and losses (conservative conditions), the internal energy of a system remains constant despite the conversion between kinetic energy and elastic potential energy. Both the kinetic and elastic energies vary between 50 and 0 $\left[\mathrm{J}\right]$. When damper (loss) is present, the total energy of the system along with the system’s velocity eventually approaches zero, and the length of the tether remains in its standard and initial state (equilibrium point).

The *beat phenomenon* is evident in Fig. 24. The beat phenomenon occurs when two harmonic waves of slightly different frequencies are impressed on a body or multibody system. The beat phenomenon is the result of the simultaneous presence of two oscillatory signals with frequencies that are close to one another, which periodically add to or subtract from each other. In this case the vibration frequency is close to (but not equal to) the natural frequency.

**Fig. 25:**
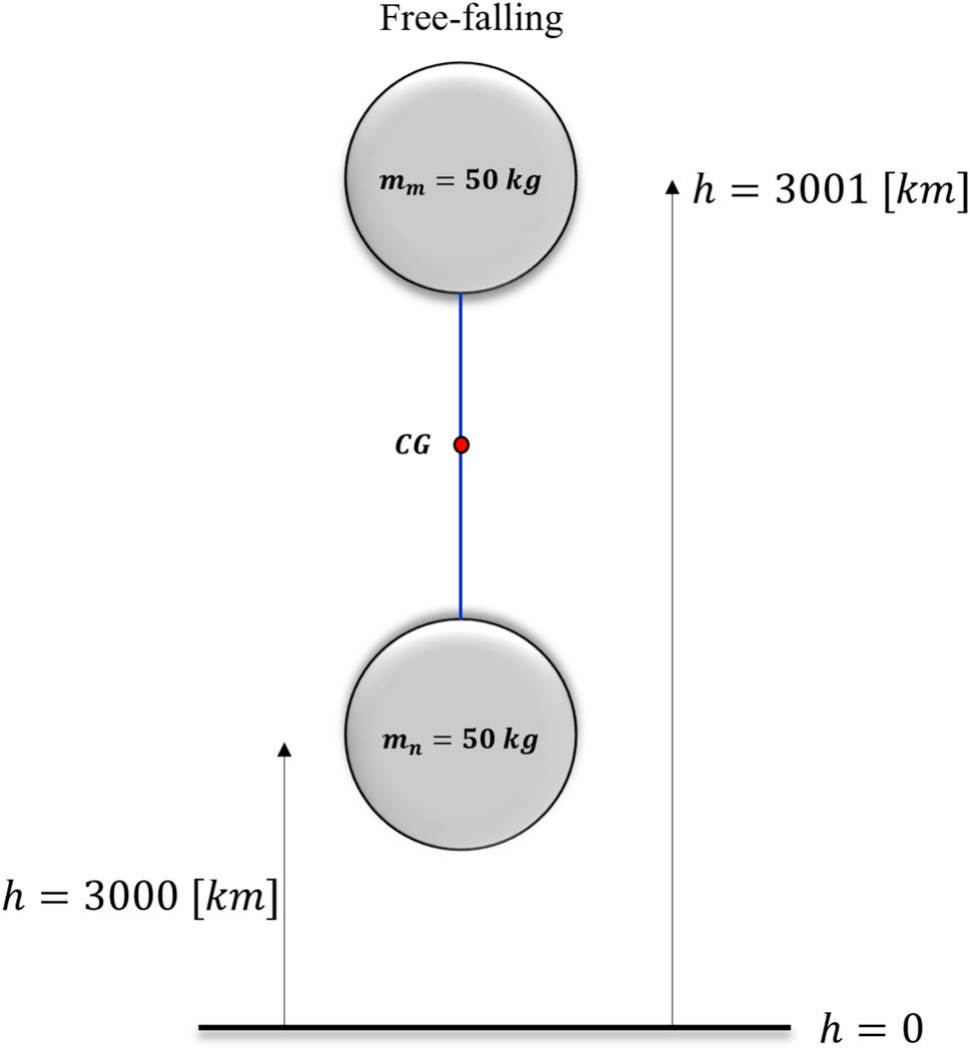
Schematic of the TSS released from an initial altitude with no initial velocity.

**Part 4:** As the last case study, we considered a tethered space system that is released from a height of $3001 \left[\mathrm{k}\mathrm{m}\right]$ from “zero potential level” with no initial velocity. The tether is straight but not stretched. The schematic of the system is shown in Fig. 25. The input data are the same as previous example, and the only difference is that gravity is also considered here.

**Fig. 26:**
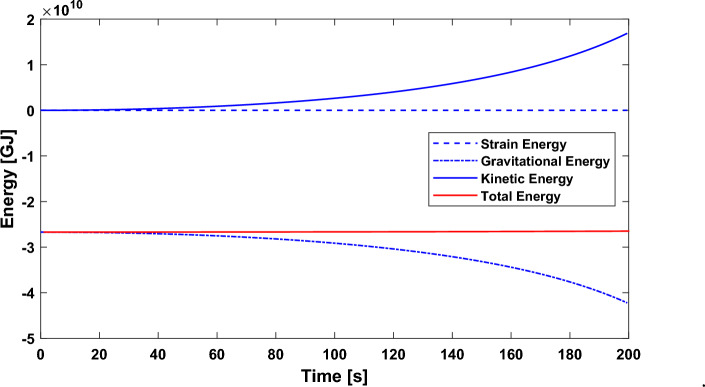
Energy time history of the free-falling system.

**Fig. 27:**
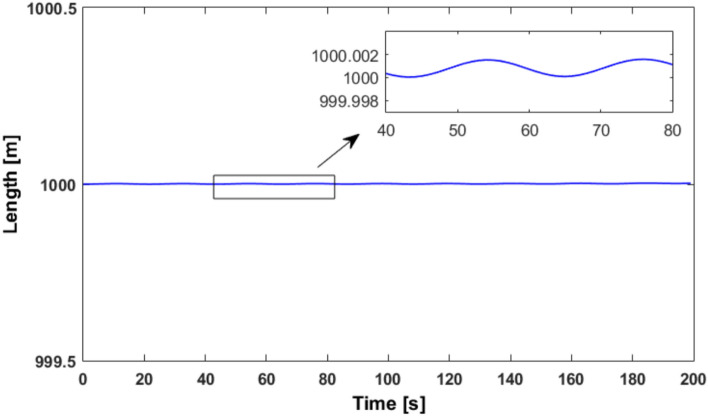
Tether length over the free-falling phase.

The energy of the TSS is drawn in Fig. 26. As can be seen in Fig. 26, the kinetic energy is initially zero and steadily grows as the system’s falling speed increases. The elastic potential energy of the tether is zero because the tether does not undergo a change in length (as is clear from Fig. 27). During fulling, gravitational potential energy has gradually increased according to the relation $-\mu m/r$. The internal energy of the system remains constant because the system is conservative.

**Fig. 28:**
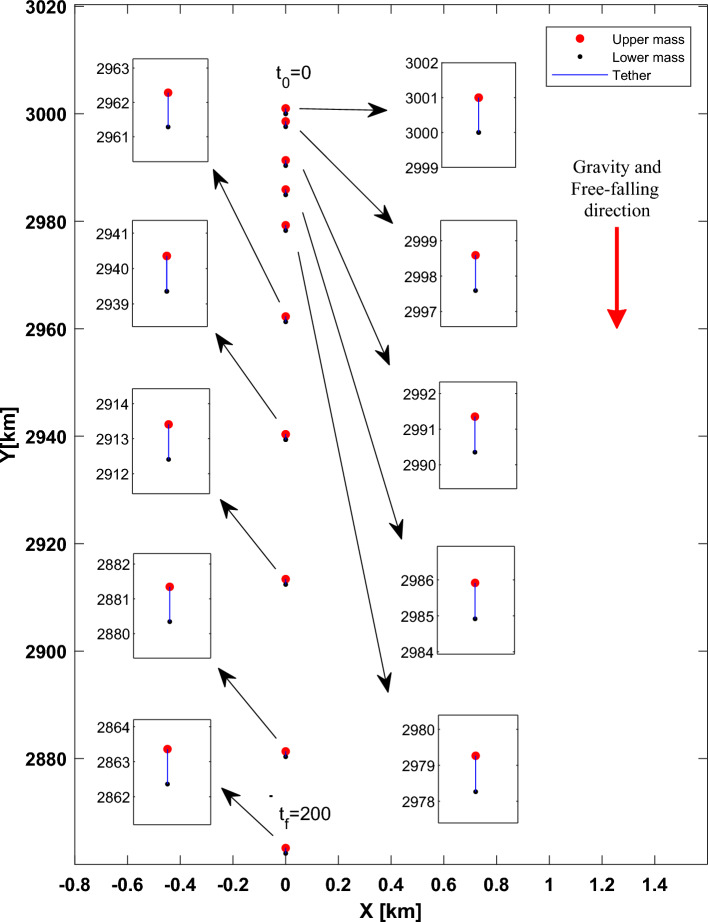
Global attitude of the free-falling tethered satellite system under gravity.

The global attitude of the tethered satellite system is depicted in Fig. 28. In this figure, the upper big circle corresponds to the mother satellite, and the lower small circle represents the robot. As expected, the tether length remains almost constant, and the system drops under the acceleration of gravity.

The simulation findings for the systems presented in Figs. 20, 21, 22, 23, 24, 25, 26, 27 and 28 are exactly what we would expect from a conservative mass–spring system.

### Main mission scenario

**Fig. 29:**
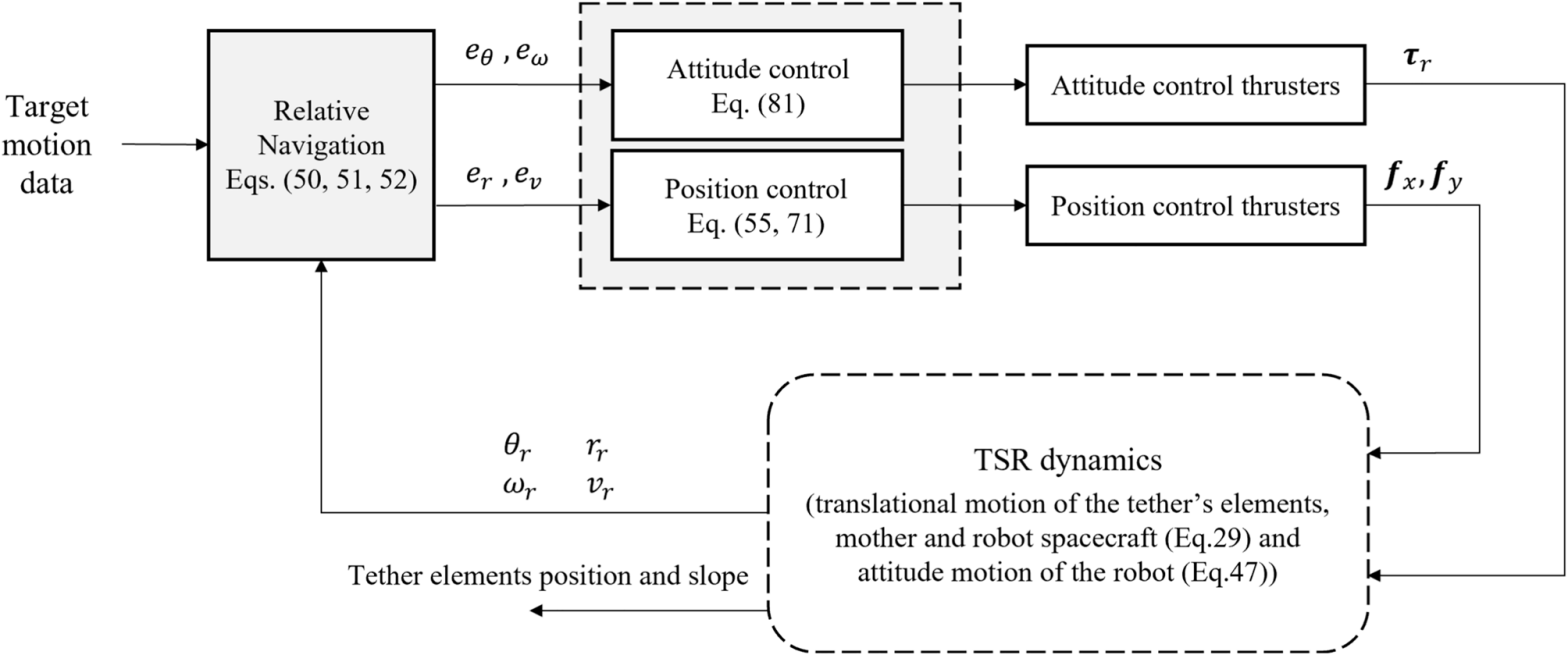
Block diagram of the closed-loop system.

The major concern of this paper is control of the TSR to synch and then dock with the target spacecraft. The main idea implemented in this study is to control the robot based on the coupled relative equations of motion to track the target’s motion, while the mutual effects of the tether on its translational and rotational dynamics are considered. Figure 29 shows the block diagram of the TSR’s overall position and attitude control.

It is noteworthy that, even if the system has Hundreds of degrees of freedom (considering numerous elements), control design concerns are minimized, as it is not necessary to control all DoF (position and velocity of all nodes on the tether). Instead, it suffices to only control the degrees of freedom of the robot (end node). In this paper, where the problem is solved in the orbital plane, the robot has three DoF (${{\boldsymbol{X}}}_{{\boldsymbol{r}}}$, ${{\boldsymbol{Y}}}_{{\boldsymbol{r}}}$, and ${\theta}_{r}$) that must be controlled, while the total system has 9 DoF (see Appendix E).

**Fig. 30:**
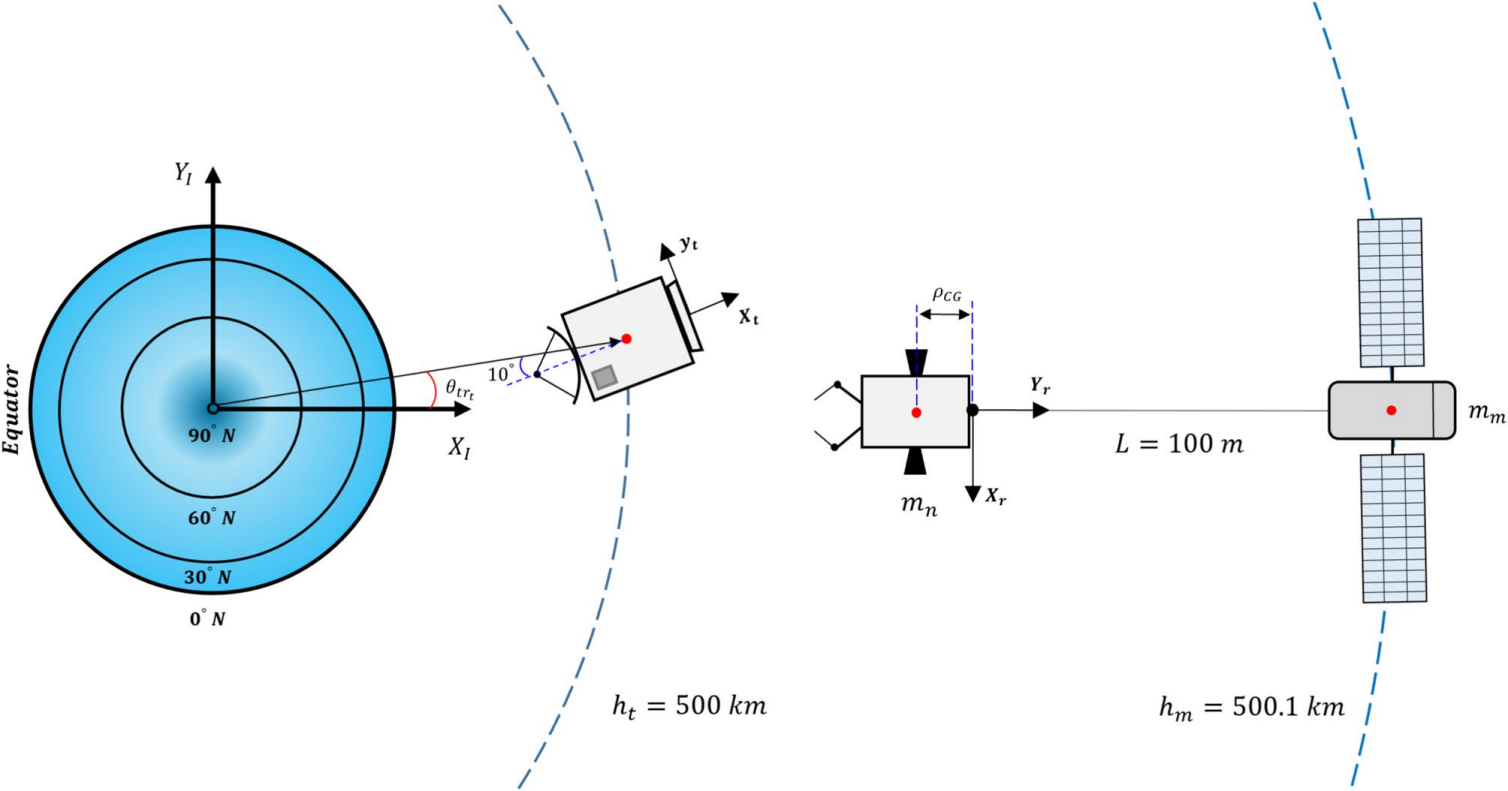
Schematic of the TSR and the target spacecraft under initial conditions.

The initial conditions of the system are shown in Fig. 30, and the information for the TSR, controller, and target spacecraft are presented in Tables 1, 2, 3 and 4.

**Table 1:** TSR characteristics.

Parameter	Symbol	Value	Unit
**Mother spacecraft**			
Mass	${m}_{m}$	500	[$\mathrm{k}\mathrm{g}$]
Dimension	${d}_{m}$	$0.8* 0.8*1.2$	[$\mathrm{m}$]
Moment of inertia	${I}_{m}$	26	[$\mathrm{k}\mathrm{g}\cdot {\mathrm{m}}^{2}$]
**Robot spacecraft**			
Mass	${m}_{r}$	40	[$\mathrm{k}\mathrm{g}$]
Dimension	${d}_{r}$	$0.2*0.2*0.42$	[$\mathrm{m}$]
Moment of inertia	${I}_{r}$	4	[$\mathrm{k}\mathrm{g}\cdot {\mathrm{m}}^{2}$]
**Tether**			
Length	$L$	100	[$\mathrm{m}$]
Mass density	$\rho$	$3.2\times {10}^{-3}$	$\left[\mathrm{k}\mathrm{g}/\mathrm{m}\right]$
Section area	$A$	$2.01\times {10}^{-6}$	[${\mathrm{m}}^{2}$]
Young’s modulus	$E$	270	$[\mathrm{G}\mathrm{P}\mathrm{a}]$
Damping coefficient	$\alpha$	0.01	$\left[\mathrm{s}\right]$
Element numbers	$N$	3	–
Distance between robot’s CG and tether’s connection point	$\left|{{\boldsymbol{\rho}}}_{{\boldsymbol{C}}{\boldsymbol{G}}}\right|$	0.1	[$\mathrm{m}$]

**Table 2:** Controller parameters.

Parameter	Value	Unit
${k}_{{p}_{x}} , {k}_{{d}_{x}}$	500, 20	–
${k}_{{p}_{y}}, {k}_{{d}_{y}}$	500, 20	–
${k}_{pp}, {k}_{dd}$	10, 20	–

**Table 3:** Initial condition.

Parameter	Symbol	Value	Unit
**Tether**			
Tether status		Completely stretched	
**Robot**			
Position vector	${{\boldsymbol{r}}}_{{\boldsymbol{r}}}$	$6878\mathrm{i}+0\mathrm{j}$	[k $\mathrm{m}$]
Attitude	${\theta}_{r}$	− 90	[$\mathrm{d}\mathrm{e}\mathrm{g}$]
Angular velocity	${\omega}_{r}$	0	$[\mathrm{d}\mathrm{e}\mathrm{g}/\mathrm{s}]$
Translational velocity	${{\boldsymbol{v}}}_{{\boldsymbol{r}}}$	$0\mathrm{i}+7.6127\mathrm{j}$	$[\mathrm{k}\mathrm{m}/\mathrm{s}]$
True anomaly	${\theta}_{{tr}_{r}}$	0	[$\mathrm{d}\mathrm{e}\mathrm{g}$]
**Target**			
Translational velocity	${{\boldsymbol{v}}}_{{\boldsymbol{t}}}$	$-0.0133\mathrm{i}+7.6127\mathrm{j}$	$[\mathrm{k}\mathrm{m}/\mathrm{s}]$
True anomaly	${\theta}_{{tr}_{t}}$	$1.25\times {10}^{-4}$	[$\mathrm{d}\mathrm{e}\mathrm{g}$]

**Table 4:** Target spacecraft characteristics in initial condition.

Parameter	Symbol	Value	Unit
Mass	${m}_{t}$	250	[$\mathrm{k}\mathrm{g}$]
Dimension	${d}_{t}$	$0.64*0.64*1.1$	[$\mathrm{m}$]
Moment of inertia	${I}_{t}$	10	[$\mathrm{k}\mathrm{g}\cdot {\mathrm{m}}^{2}$]
Position vector	${{\boldsymbol{r}}}_{{\boldsymbol{t}}}$	$6878\mathrm{cos}\left({\theta}_{{tr}_{t}}\right)i+6878\mathrm{sin}\left({\theta}_{{tr}_{t}}\right)j$	$[\mathrm{k}\mathrm{m}]$
Attitude	${\theta}_{t}$	10	$[\mathrm{d}\mathrm{e}\mathrm{g}]$
Body angular velocity	${\omega}_{t}$	Always 0	$\left[\mathrm{d}\mathrm{e}\mathrm{g}/\mathrm{s}\right]$

We assumed that the target is orbiting in a circular equatorial orbit and actively controls its attitude in ${10}^{^\circ }$ off-nadir. Also, the TSR is located along the ${X}_{I}$ axis, and the tether is completely stretched in an initial condition. The robot is expected to reach its position to the target while it has rotated $+10$ degrees around $Z$ axis.

**Fig. 31:**
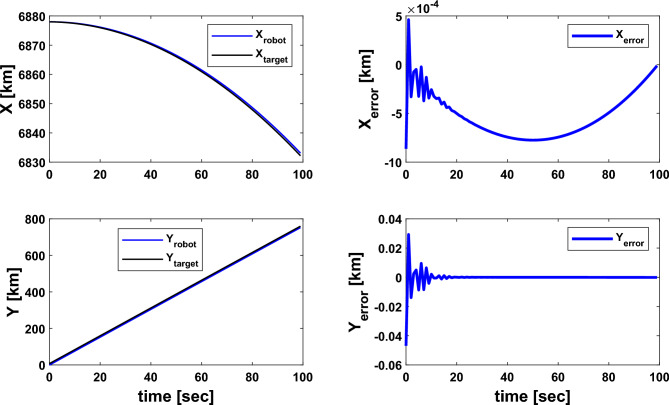
The target and chaser spacecraft positions and relative position error.

The plots of the translational motion of both the target and robot spacecraft, along with their relative position errors, are shown in Fig. 31. The plots show that the robot has successfully tracked the target’s trajectory and docking can be achieved after 100 s, which is a reasonable time for the proximity and docking phase.

**Fig. 32:**
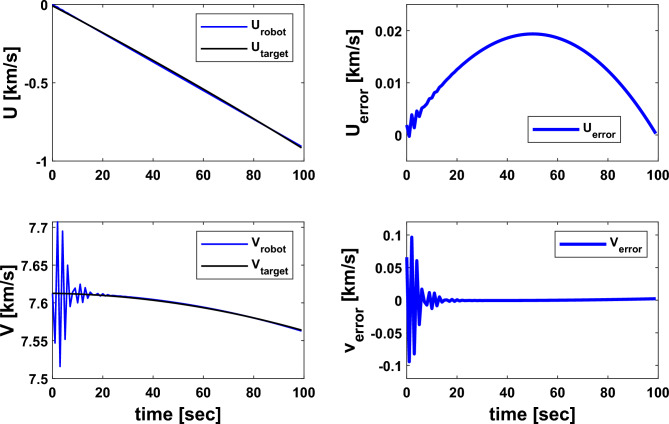
The target and chaser spacecrafts’ absolute velocities and relative velocity errors.

Similarly, Fig. 32 shows the velocity of both spacecraft (in both the horizontal and vertical directions) and their relative velocity errors. The observed pattern in the relative velocity of the two spacecraft indicates that, at one point, both the position and velocity of the two satellites are almost the same. This condition is highly conducive to safe and soft docking.

**Fig. 33:**
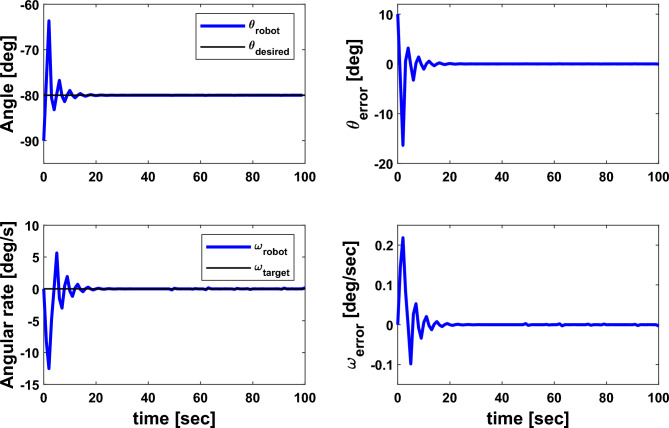
The target and chaser spacecraft attitudes and corresponding errors.

The attitude and angular velocities of the robot and target spacecraft, along with their relative orientational motion errors, are depicted in Fig. 33. According to this Figure, the relative attitude error of the two spacecraft will converge to zero in a stable manner.

An analysis of Figs. 32 and 33 reveals that the attitude and translational synchronization of the chaser and target was successfully executed, and the docking requirements have been met.

**Fig. 34:**
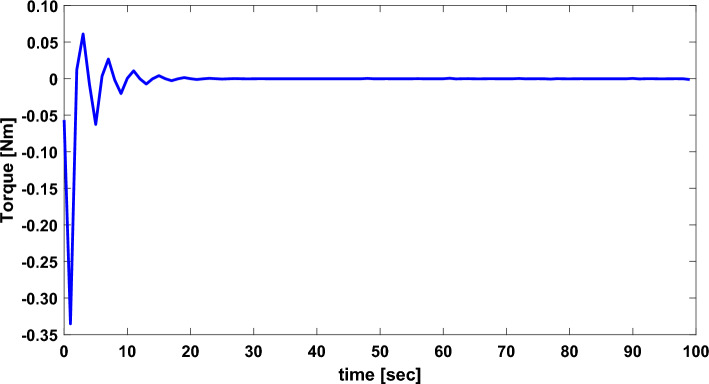
Chaser torque (orientational motion’s control effort).

**Fig. 35:**
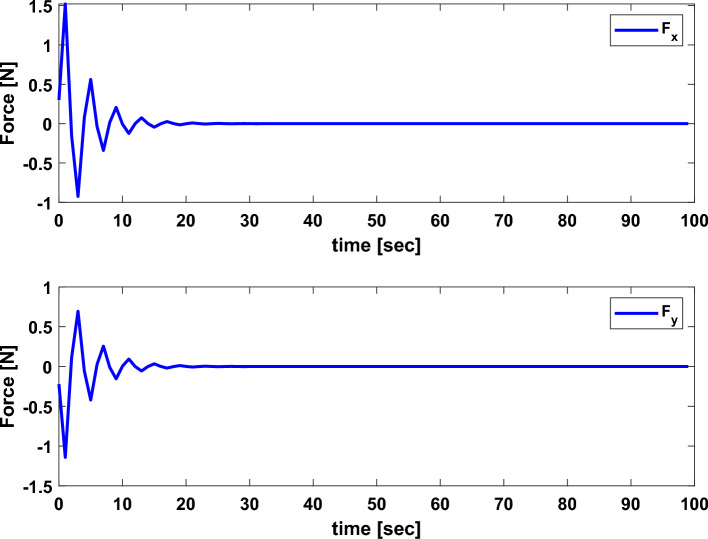
Chaser force (translational motion’s control effort).

The robot’s control torque and forces during the proximity and docking operations are shown in Figs. 34 and 35, respectively.

**Fig. 36:**
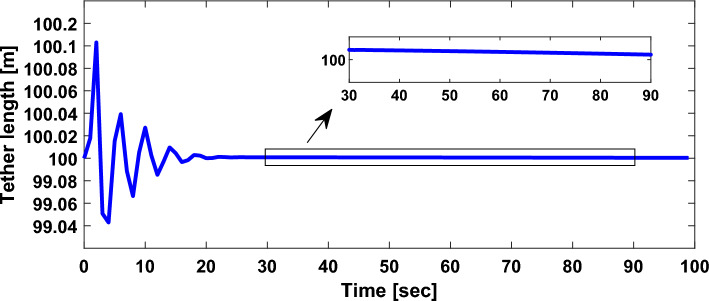
Variation in tether length.

The variation in tether length is illustrated in Fig. 36. Based on this, it can be concluded that the maximum elongation in the tether is about 10 cm.

**Fig. 37:**
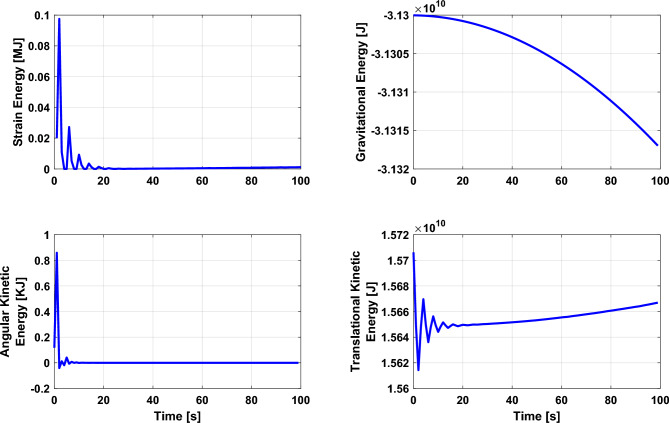
Energies of the tethered satellite system versus time.

The energy change plot is shown in Fig. 37. According to what is expected from the physics of the problem, the dominant energies are translational kinetic energy (due to orbital velocity) and gravitational potential energy (due to orbital height). The decrease in orbital altitude due to gravitational acceleration and acceleration of the robot caused by the propulsion system result in a gradual increase in both kinetic energy and gravitational potential. As expected from Figs. 33 and 36, logically, the elastic potential energy and rotational kinetic energy tend to zero after some time, because the changes in the tether and the robot’s translational motion have reached zero.

**Fig. 38:**
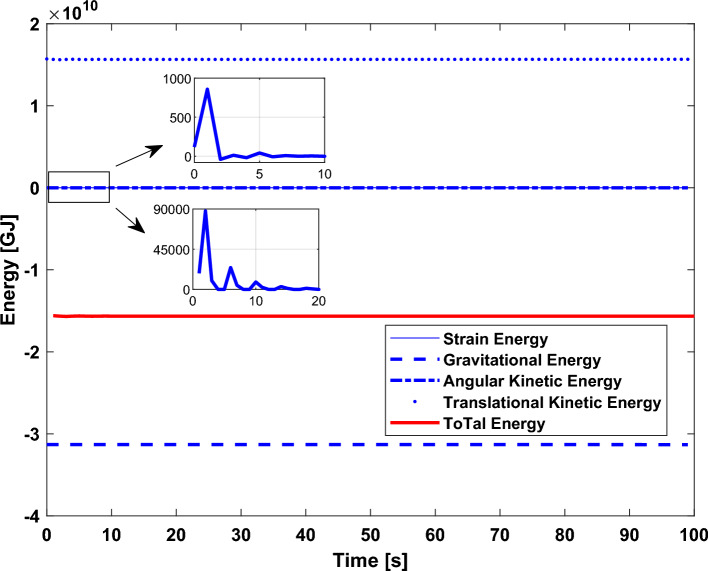
Energies of the tethered satellite system.

All the energies of the system (all four energies are drawn in Fig. 37) are drawn together in the Fig. 38, and, as can be seen, the total inner energy of the system (the solid red line) is negative, which means that the orbit of the TSR is a closed orbit (not parabolic or hyperbolic), and gradually its height decreases.

**Fig. 39:**
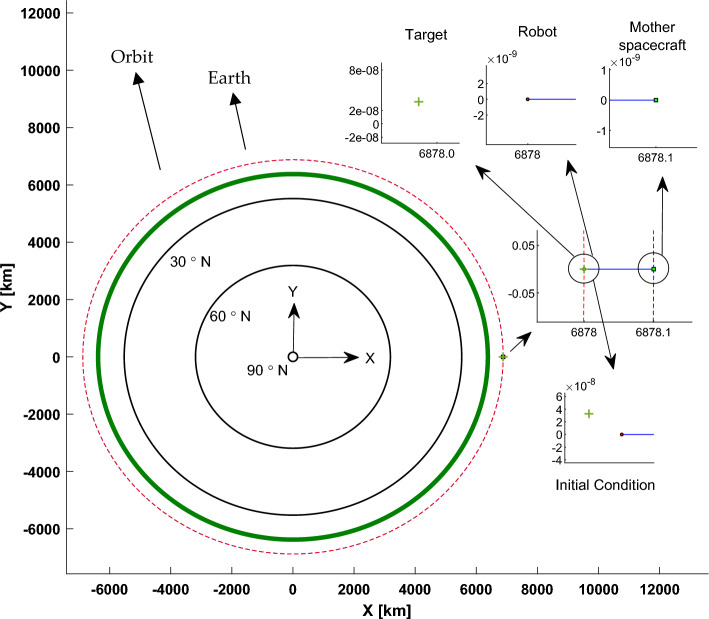
TSR and the target spacecraft position under initial conditions.

Figure 39 shows the positions of the TSR and the target spacecraft in an initial condition. As shown in Fig. 30, the scenario begins with the TSR positioned along the x-axis at an altitude of approximately 500 km, and the tether is completely straight and taut. In the initial conditions, the robot and the target are separated, and the true anomaly of the robot is zero. In Fig. 39, the green solid circle represents the Earth’s surface, and the blue dashed line and the red dashed line represent the orbits of the mother spacecraft and the target, respectively. Note that the Earth is shown from the top view, and both the target and TSR are orbiting in equatorial orbit.

**Fig. 40:**
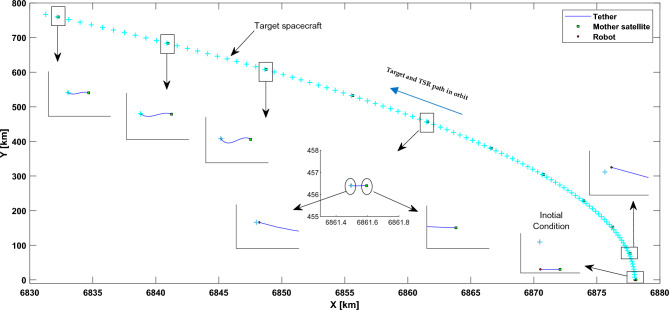
TSR and the target spacecraft position during mission.

The motion of the TSR is shown in Fig. 39 by snapshots during the docking phase. In this figure, the position of the target, which is displayed with a plus sign, is drawn every 1 s, but the position of the TSR is drawn every 10 s. In fact, Fig. 40 is a different representation of Fig. 39, where the Earth has been removed, and the motion of the robot and the target satellite in the orbit is depicted over a period of 200 s. As can be seen, the robot has moved from its initial condition (the first plot in the bottom right of Fig. 40) to a position very close to the target for docking operations (the left side plot in Fig. 40). The robot moves from the initial conditions (in which its position only has the x-component, as shown in Figs. 30 and 39) towards the target and reduces its distance to the target based on the relative navigation and produced control forces.

## Conclusions

In order to increase the lifespan of valuable satellites, an active on-orbit servicing system based on a TSR is proposed in this study for refueling missions. This paper presented the system components, mission sequence, mathematical modeling, and controller design.

For the first time, the robot attached to the end of the tether is considered as an active controllable spacecraft in this work. We model the dynamic of the tether using the ANCF method, which is very accurate for flexible tethers with large deformations. The robot has been considered as a rigid body that controls its position and attitude. The complexity of the challenge arises from the fact that the tether’s connection point does not coincide with the robot’s center of gravity, which means that the movement of each of them affects the other. The interaction effects of the robot and tether movements on each other have been taken into account, which, of course, has led to coupled and highly nonlinear dynamic and kinematic equations. The relative translational and orientational motion equations have been used to find the desired path for docking. Sliding mode control law have been designed to meet the requirements of the close-range rendezvous phase (omitting the relative distance and attitude while the linear and angular velocities are synched). Comprehensive numerical simulations were conducted based on a sample scenario to show that the TSR is a feasible and practical option for on-orbit refueling purposes.

## Suggestions for future work

Addressing the stage of tether deployment (stages 2 and 3 of Fig. 4) in the presence of an active robot is one of the aspects that requires further research, and it is expected to yield interesting results. To this end, the “Absolute Nodal Coordinate Formulation method in a framework of Arbitrary Lagrange–Euler (ANCF-ALE)” method should be used instead of the conventional “ANCF” method, as, with the tether deployment, the time-varying mass matrix will result in a varying number of equations (element numbers of the tether) to be solved at each step. In this case, the mass of the mother satellite will also be time-varying and should be calculated based on the deployment rate of the tether and its mass density.

Investigating the impact of parameters such as lateral stretch, torsion, and their associated inertia on the behavior and curvature of the tether can also be a valuable research topic that brings our knowledge and perception of space tether systems closer to reality.

## Figures and Tables

**Fig. 41:**
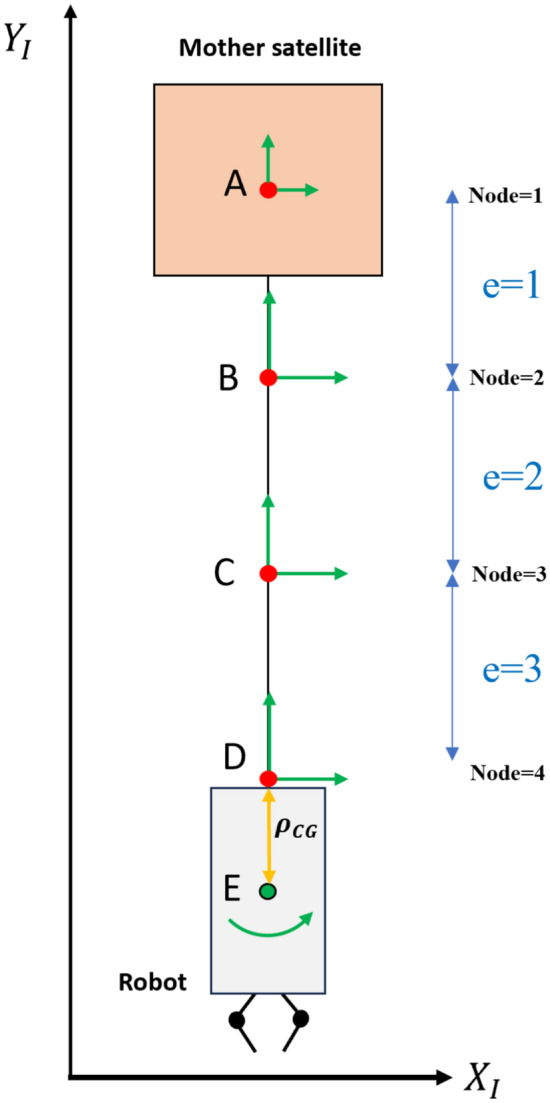
System’s degrees of freedom.

## Data Availability

The authors confirm that all the data supporting the findings of this study are available within the article.

## Appendix A: Calculation of ${\mathbf{N}}^{\mathbf{T}}\mathbf{N}$

$${\mathbf{N}}^{\mathrm{T}}\mathbf{N}=\left[\begin{array}{c}{\mathrm{N}}_{1}^{2}\\ 0\\ {\mathrm{N}}_{1}{\mathrm{N}}_{2}\\ 0\\ {\mathrm{N}}_{1}{\mathrm{N}}_{3}\\ 0\\ {\mathrm{N}}_{1}{\mathrm{N}}_{4}\\ 0\end{array} \begin{array}{c}0\\ {\mathrm{N}}_{1}^{2}\\ 0\\ {\mathrm{N}}_{1}{\mathrm{N}}_{2}\\ 0\\ {\mathrm{N}}_{1}{\mathrm{N}}_{3}\\ 0\\ {\mathrm{N}}_{1}{\mathrm{N}}_{4}\end{array} \begin{array}{c}{\mathrm{N}}_{1}{\mathrm{N}}_{2}\\ 0\\ {\mathrm{N}}_{2}^{2}\\ 0\\ {\mathrm{N}}_{2}{\mathrm{N}}_{3}\\ 0\\ {\mathrm{N}}_{2}{\mathrm{N}}_{4}\\ 0\end{array} \begin{array}{c}0\\ {\mathrm{N}}_{1}{\mathrm{N}}_{2}\\ 0\\ {\mathrm{N}}_{2}^{2}\\ 0\\ {\mathrm{N}}_{2}{\mathrm{N}}_{3}\\ 0\\ {\mathrm{N}}_{2}{\mathrm{N}}_{4}\end{array} \begin{array}{c}{\mathrm{N}}_{1}{\mathrm{N}}_{3}\\ 0\\ {\mathrm{N}}_{2}{\mathrm{N}}_{3}\\ 0\\ {\mathrm{N}}_{3}^{2}\\ 0\\ {\mathrm{N}}_{3}{\mathrm{N}}_{4}\\ 0\end{array} \begin{array}{c}0\\ {\mathrm{N}}_{1}{\mathrm{N}}_{3}\\ 0\\ {\mathrm{N}}_{2}{\mathrm{N}}_{3}\\ 0\\ {\mathrm{N}}_{3}^{2}\\ 0\\ {\mathrm{N}}_{3}{\mathrm{N}}_{4}\end{array} \begin{array}{c}{\mathrm{N}}_{1}{\mathrm{N}}_{4}\\ 0\\ {\mathrm{N}}_{2}{\mathrm{N}}_{4}\\ 0\\ {\mathrm{N}}_{3}{\mathrm{N}}_{4}\\ 0\\ {\mathrm{N}}_{4}^{2}\\ 0\end{array} \begin{array}{c}0\\ {\mathrm{N}}_{1}{\mathrm{N}}_{4}\\ 0\\ {\mathrm{N}}_{2}{\mathrm{N}}_{4}\\ 0\\ {\mathrm{N}}_{3}{\mathrm{N}}_{4}\\ 0\\ {\mathrm{N}}_{4}^{2}\end{array}\right]$$

## Appendix B: Mass matrix

$${{\boldsymbol{M}}}_{1}={{\boldsymbol{m}}}_{m}+{{\boldsymbol{m}}}_{tether}=\frac{\rho l}{420}\left[\begin{array}{c}156I\\ 22lI\\ 54I\\ -13lI\end{array} \begin{array}{c}\\ 2{l}^{2}I\\ 13lI\\ -3{l}^{2}I\end{array} \begin{array}{c}\\ \\ 156I\\ -22lI\end{array} \begin{array}{c}\\ \\ \\ 4{l}^{2}I\end{array}\right]+\left[\begin{array}{c}{m}_{m}I\\ 0I\\ 0I\\ 0I\end{array} \begin{array}{c}0I\\ 0I\\ 0I\\ 0I\end{array} \begin{array}{c}0I\\ 0I\\ 0I\\ 0I\end{array} \begin{array}{c}0I\\ 0I\\ 0I\\ 0I\end{array}\right]\,for\, e=1$$

$${{\boldsymbol{M}}}_{e}={{\boldsymbol{m}}}_{tether}=\sum_{2}^{N-1}\frac{l}{2}{\int}_{-1}^{1}\rho {{\boldsymbol{N}}}^{T}{\boldsymbol{N}}d\xi =\sum_{2}^{N-1}\frac{\rho l}{420}\left[\begin{array}{c}156I\\ 22lI\\ 54I\\ -13lI\end{array} \begin{array}{c}\\ 2{l}^{2}I\\ 13lI\\ -3{l}^{2}I\end{array} \begin{array}{c}\\ \\ 156I\\ -22lI\end{array} \begin{array}{c}\\ \\ \\ 4{l}^{2}I\end{array}\right]\, for\, e=2:N-1$$

$${{\boldsymbol{M}}}_{last}={{\boldsymbol{m}}}_{tether}+{{\boldsymbol{m}}}_{r}=\frac{\rho l}{420}\left[\begin{array}{c}156I\\ 22lI\\ 54I\\ -13lI\end{array} \begin{array}{c}\\ 2{l}^{2}I\\ 13lI\\ -3{l}^{2}I\end{array} \begin{array}{c}\\ \\ 156I\\ -22lI\end{array} \begin{array}{c}\\ \\ \\ 4{l}^{2}I\end{array}\right]+\left[\begin{array}{c}0I\\ 0I\\ 0I\\ 0I\end{array} \begin{array}{c}0I\\ 0I\\ 0I\\ 0I\end{array} \begin{array}{c}0I\\ 0I\\ {m}_{r}I\\ 0I\end{array} \begin{array}{c}0I\\ 0I\\ 0I\\ 0I\end{array}\right]\, for\, e=N$$

The total mass matrix is equal to the summation of the mass matrix of the all elements (${{\boldsymbol{M}}}_{1} to {{\boldsymbol{M}}}_{last}$).

Note that this mass matrix is only related to the translational motion of the system, and its size is $\left[8\mathrm{N} \times 8\mathrm{N}\right]$.

It is obvious that the system’s differential equations, represented by Eqs. (29) and (47), are second-order. In order to employ numerical techniques like the Runge–Kutta method in MATLAB for their solution, it is imperative to convert these equations into a set of first-order equations. Hence, the comprehensive mass matrix that incorporates the equations governing rotational and translational motion can be expressed as described below.

It should be noted that the matrix provided in this context pertains to a tether consisting of a single element, which has two nodes. This simplification has been employed to enhance clarity and ease of understanding.

The style presented here is analogous to the common and widely recognized “$m\ddot{x}+C\dot{x}+Kx=F$” format, which is extensively employed in the modeling of robots and dynamic systems.

where ${a}_{ij}$ and ${d}_{ij}$ are defined as:

$$\begin{aligned} a_{22} & = \rho l\frac{156}{{420}}\quad a_{65} = \rho l^{3} \frac{4}{420}\quad a_{69} = \rho l^{2} \frac{13}{{420}}\quad a_{210} = \rho l\frac{54}{{420}} \\ a_{214} & = - \rho l^{2} \frac{13}{{420}}\quad a_{613} = \rho l^{3} \frac{ - 3}{{420}}\quad d_{22} = \frac{{\delta_{eN} m_{r} }}{{I_{r} }}R_{r}^{g} \left( {1,1} \right)\quad d_{24} = \frac{{\delta_{eN} m_{r} }}{{I_{r} }}R_{r}^{g} \left( {2,2} \right) \\ \end{aligned}$$

${d}_{22}$ and ${d}_{24}$ define the interaction of robot rotational motion and the last node translational motion.

Hence, the dimension of the mass matrix is $\left[18\times 18\right]$, with 16 lines pertaining to translational motion of the system and the remaining two lines pertaining to attitude motion of the robot.

It is crucial to remember that this matrix is offered for tethers with a single element. As with all finite element approaches, the matrices must be assembled for a tether with more elements.

## Appendix C: Force and toque vector

The vector ${{\boldsymbol{F}}}_{ext}+{{\boldsymbol{\tau}}}_{r}$ can alternatively be expressed in the following format: (according to the underlying assumption presented in this article, it is evident that no external force is directly exerted on the mother spacecraft):

## Appendix D: Strain calculation

According to^103^ we can develop $\upvarepsilon$ as follows:

$$\upvarepsilon =\left(\sqrt{{r}^{\prime T}{r}^{{\prime}}}-1\right)$$

$${r}^{{\prime}}=\frac{\partial r}{\partial s}=\frac{\partial N{X}_{e}}{\partial s}=\frac{\partial N}{\partial s}{X}_{e}={N}^{{\prime}}{X}_{e}$$

$$\mathrm{f}\mathrm{r}\mathrm{o}\mathrm{m}\, \mathrm{E}\mathrm{q}\mathrm{u}\mathrm{a}\mathrm{t}\mathrm{i}\mathrm{o}\mathrm{n} \left(2\right) \mathrm{w}\mathrm{e} \mathrm{h}\mathrm{a}\mathrm{v}\mathrm{e}: \upvarepsilon =\sqrt{{{x\left(s,t\right)}^{\prime2}}+{{y\left(s,t\right)}^{{\prime2}}}}-1$$

$$\upvarepsilon =\frac{2}{l}\left(\sqrt{{\left({N}_{\xi }\left(1,:\right){X}_{e}\right)}^{2}+{\left({N}_{\xi }\left(2,:\right){X}_{e}\right)}^{2}}\right)-1$$

where ${N}_{\xi }\left(1,:\right)$ and ${N}_{\xi }\left(2,:\right)$ are the first and second rows of matrix ${N}_{\xi }$.

$$\frac{\partial \varepsilon }{\partial {X}_{e}}=\left({N}^{{{\prime T}}}{N}^{{\prime}}\right){X}_{e}=\frac{2}{l}\left(\frac{{{N}_{\xi }\left(1,:\right)}^{2}+{{N}_{\xi }\left(2,:\right)}^{2}}{\sqrt{{\left({N}_{\xi }\left(1,:\right){X}_{e}\right)}^{2}+{\left({N}_{\xi }\left(2,:\right){X}_{e}\right)}^{2}}}\right){X}_{e}$$

where $${N}_{\xi }=\left[\begin{array}{c}{N}_{{\xi}_{1}}\\ 0\end{array} \begin{array}{c}0\\ {N}_{{\xi}_{1}}\end{array} \begin{array}{c}{N}_{{\xi}_{2}}\\ 0\end{array} \begin{array}{c}0\\ {N}_{{\xi}_{2}}\end{array} \begin{array}{c}{N}_{{\xi}_{3}}\\ 0\end{array} \begin{array}{c}0\\ {N}_{{\xi}_{3}}\end{array} \begin{array}{c}{N}_{{\xi}_{4}}\\ 0\end{array} \begin{array}{c}0\\ {N}_{{\xi}_{4}}\end{array}\right]$$ and ${N}_{{\xi}_{i}}$, $i=\mathrm{1,2},\mathrm{3,4}$ are defined as follows:

$$\begin{aligned} N_{{\xi_{1} }} & = \frac{{\partial N_{1} }}{\partial \xi } = \frac{1}{4}\left( {3\xi^{2} - 3} \right)\quad N_{{\xi_{2} }} = \frac{{\partial N_{2} }}{\partial \xi } = \frac{l}{8}\left( {3\xi^{2} - 2\xi - 1} \right) \\ N_{{\xi_{3} }} & = \frac{{\partial N_{3} }}{\partial \xi } = \frac{1}{4}\left( { - 3\xi^{2} + 3} \right)\quad N_{{\xi_{4} }} = \frac{{\partial N_{4} }}{\partial \xi } = \frac{l}{8}\left( {3\xi^{2} + 2\xi - 1} \right) \\ \end{aligned}$$

Now, the calculation of “$\dot{\upvarepsilon }$” has become more straightforward.

$$\dot{\upvarepsilon }=\left(\frac{2}{l}\left(\sqrt{{\left({N}_{\xi }\left(1,:\right)\right)}^{2}+{\left({N}_{\xi }\left(2,:\right)\right)}^{2}}\right)\right){\dot{X}}_{e}$$

For more detail about ${\dot{X}}_{e}$ please see^114^.

## Appendix E: Degree of freedom

If the number of elements in the TSR—as illustrated in the figure below—is equal to 3, then a total of 9 degrees of freedom must be considered for the system.

1. The mother satellite (point A) moves in a circular Keplerian orbit, thus possessing 2 degrees of freedom (in the global inertial coordinate system).
2. The two midpoint points located on the tether (points B and C) each have 2 degrees of freedom (only translational motion).
3. Point D, which is the terminal node of the tether and is connected to the robot, also has 2 degrees of freedom (translational motion).
4. The robot, due to its rotational motion, will have 1 degree of freedom.

It is important to note that since the robot is a rigid body, the positions of points D and E in the Fig. 41 are not independent of each other. They are related through ${\rho}_{CG}$ (the distance between the tether connection point and the center of mass of the robot) and the robot’s attitude, represented by the angle ${\theta}_{r}$ in Fig. 15. In other words, the translational degrees of freedom (linear motion in the x and y directions on the plane) of points D and E are not independent; therefore, it is inappropriate to consider separate degrees of freedom for both point D and point E.

Please note that the entire mother satellite is treated as a point mass because it is assumed that its position and attitude will not change during the robot’s operation (which is also brief).
